# Rapid assessment of insect pollination services to inform decision‐making

**DOI:** 10.1111/cobi.13886

**Published:** 2022-03-08

**Authors:** Fabrizia Ratto, Tom D. Breeze, Lorna J. Cole, Michael P. D. Garratt, David Kleijn, Bill Kunin, Denis Michez, Rory O'Connor, Jeff Ollerton, Robert J. Paxton, Guy M. Poppy, Simon G. Potts, Deepa Senapathi, Rosalind Shaw, Lynn V. Dicks, Kelvin S.‐H. Peh

**Affiliations:** ^1^ School of Biological Sciences University of Southampton Southampton UK; ^2^ School of Biology, Faculty of Biological Sciences University of Leeds Leeds UK; ^3^ Centre for Agri‐Environmental Research, School of Agriculture, Policy and Development University of Reading Reading UK; ^4^ Integrated Land Management, Environment & Society SRUC Ayr UK; ^5^ Resource Ecology Group Wageningen University and Research Wageningen The Netherlands; ^6^ Laboratoire de Zoologie Université de Mons Mons Belgium; ^7^ Faculty of Arts, Science and Technology University of Northampton Northampton UK; ^8^ General Zoology Martin Luther University Halle‐Wittenberg, Halle, Germany; ^9^ German Centre for Integrative Biodiversity Research (iDiv) Halle‐Jena‐Leipzig Leipzig Germany; ^10^ Environment and Sustainability Institute University of Exeter Penryn UK; ^11^ Conservation Science Group, Department of Zoology University of Cambridge Cambridge UK; ^12^ School of Biological Sciences University of East Anglia Norwich UK

**Keywords:** dependency ratio, ecosystem services, exclusion experiment, field beans, insect pollinators, oilseed rape, TESSA, visitation frequency, colza, experimento de exclusión, frecuencia de visita, haba común, índice de dependencia, insectos polinizadores, servicios ambientales, TESSA, 依赖率, 生态系统服务, 排除性实验, 蚕豆, 昆虫授粉者, 油菜, 生态系统服务站点评估工具包(TESSA), 访花频率

## Abstract

Pollinator declines have prompted efforts to assess how land‐use change affects insect pollinators and pollination services in agricultural landscapes. Yet many tools to measure insect pollination services require substantial landscape‐scale data and technical expertise. In expert workshops, 3 straightforward methods (desk‐based method, field survey, and empirical manipulation with exclusion experiments) for rapid insect pollination assessment at site scale were developed to provide an adaptable framework that is accessible to nonspecialist with limited resources. These methods were designed for TESSA (Toolkit for Ecosystem Service Site‐Based Assessment) and allow comparative assessment of pollination services at a site of conservation interest and in its most plausible alternative state (e.g., converted to agricultural land). We applied the methods at a nature reserve in the United Kingdom to estimate the value of insect pollination services provided by the reserve. The economic value of pollination services provided by the reserve ranged from US$6163 to US$11,546/year. The conversion of the reserve to arable land would provide no insect pollination services and a net annual benefit from insect‐pollinated crop production of approximately $1542/year (US$24∙ha^–1^∙year^–1^). The methods had wide applicability and were readily adapted to different insect‐pollinated crops: rape (*Brassica napus*) and beans (*Vicia faba*) crops. All methods were rapidly employed under a low budget. The relatively less robust methods that required fewer resources yielded higher estimates of annual insect pollination benefit.

## Abstract 2

*Article impact statement*: Insect pollination services can be assessed rapidly under varying budgets with desk‐based methods, field surveys, and exclusion experiments.

## Abstract (es)

Diversidad y Conservación de Gasterópodos Subterráneos de Agua Dulce en los Estados Unidos y en México

**Resumen:**

Las declinaciones de los polinizadores han impulsado los esfuerzos por evaluar cómo el cambio del uso de suelo afecta a los insectos polinizadores y los servicios de polinización en los paisajes agrícolas. Aun así, muchas de las herramientas para medir los servicios de los insectos polinizadores requieren datos sustanciales a escala de paisaje y el conocimiento de expertos. Desarrollamos tres métodos sencillos (método de gabinete, censo de campo y manipulación empírica con experimentos de exclusión) durante algunos talleres de expertos para la evaluación rápida de la polinización por insectos a escala de sitio con el objetivo de proporcionar un marco de trabajo adaptable y accesible para quienes no son especialistas y cuentan con recursos limitados. Estos métodos fueron diseñados para TESSA (Toolkit for Ecosystem Service Site‐Based Assessment, en inglés) y permiten la evaluación comparativa de los servicios de polinización en los sitios de interés para la conservación y su estado alternativo más plausible (p. ej.: convertido a suelo agrícola). Aplicamos los métodos en una reserva natural del Reino Unido para estimar el valor de los servicios de polinización por insectos que proporciona la reserva. El valor económico de los servicios de polinización que proporciona la reserva varió desde US$6,163 a US$11,546 al año^‐1^. La conversión de la reserva a suelo arable no proporcionaría servicios de polinización por insectos, pero sí un beneficio anual neto a partir de la producción de cultivos polinizados por insectos de aproximadamente $1,542 al año^‐1^ (US$24 ha^‐1^ año^‐1^). Los métodos tuvieron una aplicabilidad generalizada y estaban ya adaptados a los diferentes cultivos polinizados por insectos: cultivos de colza (*Brassica napus*) y habas (*Vicia faba*). Todos los métodos pudieron usarse con bajo presupuesto. Los métodos relativamente menos robustos que requirieron menos recursos produjeron estimados más elevados del beneficio anual de la polinización por insectos.

## 【摘要】

昆虫授粉服务的快速评估为决策提供依据

授粉者数量下降推动了评估土地利用变化如何影响农业景观中昆虫授粉者和授粉服务的研究工作。然而, 许多评估昆虫授粉服务的工具都需要大量景观尺度的数据和专业技术。我们通过专家研讨会开发了3种简单的方法(案头研究方法、野外调查和利用排除性实验的经验操作), 可用于快速评估研究站点的昆虫授粉情况, 为资源有限的非专业人员提供了一个适应性框架。我们的方法是为生态系统服务站点评估工具包(TESSA)设计的, 可以在具有保护意义的站点和其最合理的替代状态下(如转换为农业用地)对授粉服务进行比较评估。我们在英国一个自然保护区应用这些方法估计了该保护区提供的昆虫授粉服务的价值。结果显示, 保护区提供的授粉服务的经济价值从6,163美元/年到11,546美元/年不等。保护区如果转换为耕地, 则不能提供昆虫授粉服务, 而昆虫授粉作物的生产则可以提供的约1,542美元/年(24美元/公顷/年)的年净收益。我们的方法具有广泛的适用性, 可用于不同的昆虫授粉作物, 如油菜(*Brassica napus*)和蚕豆(*Vicia faba*)。以上方法都可以在低预算的情况下快速使用。需要资源较少但相对较不稳健的方法会得到更高的昆虫授粉年效益估计值。**【翻译:胡怡思;审校:聂永刚】**

## INTRODUCTION

The information resulting from ecosystem service assessments is useful to a wide range of stakeholders, decision makers, and nongovernmental organizations to highlight the importance of ecosystem services for humans and biodiversity (Neugarten et al., 2018). Biotic pollination plays a key role in enhancing yield and quality in three‐quarters of major food crops globally (Klein et al., 2007) and contributes an annual market value of US$235–577 billion worldwide (Potts et al., 2016). Pollination by at least 350,000 species of animals is responsible for maintaining reproduction in over 300,000 flowering plants (Ollerton, 2017).

The majority of pollinators are insects that require foraging and nesting resources in natural, seminatural, and managed areas across agricultural landscapes (Kennedy et al., 2013). Insect pollinator richness and visitation to crop flowers declines as isolation from natural areas increases (Garibaldi et al., 2011). Some management decisions, such as conversion of natural areas to agricultural uses, result in reduced pollination services in agricultural fields (e.g., Ricketts & Lonsdorf, 2013), adversely affecting crop production (Dainese et al., 2019).

Assessing ecosystem services can support advocacy for site conservation or restoration. Guidance on how to incorporate pollination services assessment in ecosystem services tools, such as ARIES and InVEST, often requires detailed land‐cover information and substantial technical expertise (Neugarten et al., 2018). For example, ARIES models use a collaborative software in which artificial intelligence pairs spatial data with ecosystem services models (Neugarten et al., 2018). Simple methods that quantify pollination services and their economic value at a local scale would elucidate the consequences of land‐use changes to pollination service provision based on locally relevant data (Peh et al., 2013). This would enable conservation practitioners to assess a wide set of counterfactuals and provide simple instructions to staff and volunteers on how to collect or collate data needed to measure services at individual sites. Low‐budget methods can provide service estimates that are robust enough for effective advocacy, without expending considerable resources or technical knowledge. To ensure accessibility to practitioners in low‐income countries, such methods should be freely available and adaptable to a range of financial and technical resources.

## METHODS

We developed practical methods for assessing insect pollination services for the Toolkit for Ecosystem Service Site‐Based Assessment version 2.0 (TESSA; Peh et al., 2017) (background on TESSA project in Appendix S1). These methods were designed to include key TESSA features (Peh et al., 2013). Hence, they had to be straightforward and low cost; usable by nonexperts lacking technical knowledge; enable cost–benefit analyses between the focal site and a counterfactual (i.e., the most plausible alternative state); and generate data to inform local decisions on land use.

We examined 3 different methods for the assessment of insect pollination services: use of existing data sets (desk‐based approach) and 2 methods that also include local field data. Including locally relevant field data, if resources permit, is important because it allows for consideration of local insect pollinators with different levels of sensitivity to land‐use change at a site and accounts for their foraging range. To our knowledge, these methods have not been used in rapid ecosystem service assessments. We applied the 3 methods, separately, to value pollination services provided by a nature reserve. We examined their usability at the reserve and compared estimates among methods.

### Expert workshop

An expert elicitation process (2‐day workshop) was used to develop practical methods of valuing insect pollination services provided by natural or seminatural areas (e.g., a nature reserve) for TESSA (Peh et al., 2013). Twelve insect pollination scientists based in the United Kingdom or continental Europe participated (Appendix S2).

### TESSA methods for insect pollination service assessment

**FIGURE 1:**
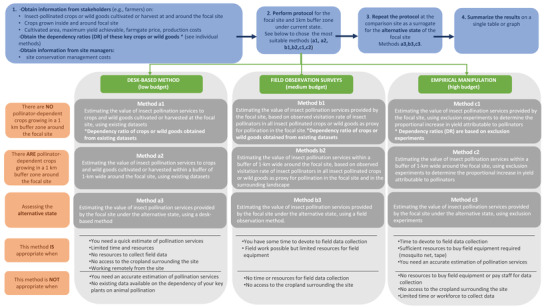
Flowchart detailing the steps to follow to perform the insect pollination service protocol and guiding the selection the most appropriate method. Dependency ratio is defined as the proportional increase in yield directly attributable to pollinators. The buffer is the area within 1‐km radius from the focal site

Following the TESSA framework (Appendix S1) (Peh et al., 2013), the experts proposed 3 site‐based protocols—suitable for nonspecialists with varying degrees of financial and time constraints—to estimate the economic value of insect pollination services contributed by a seminatural site of conservation interest: desk‐based methods for users with low budget (method a1, a2, a3); field surveys for users with medium budget (method b1, b2, b3); and empirical manipulation with exclusion experiments for users with high budget (method c1, c2, c3) (Figure 1).

These methods allow users to determine the economic effects of losing insect pollination services due to a change in land use (with varying degrees of accuracy and reliability depending on the resource availability) on economically important crops and harvested wild goods (e.g., food, energy). Economic values can be calculated for pollinator‐dependent crops or harvested wild goods at a site under current conditions and within 1 km of the site for up to 5 crops. The methods also allow a comparative assessment between a site in its current state and the same area an alternative state (e.g., converted to agricultural land). Guidance on how to determine the alternative state of a site is in Appendix S1.

An insect pollination service assessment, regardless of the method used, broadly follows the same steps (Figure 1). The flow diagram in Figure 1 is a guide for choosing the appropriate method based on availability of resources (budget, workforce, and time). Costs and time requirements are in Table 1. Details for each method are in Appendix S3 and guidance on identification and field observation of insect pollinators and common dependency‐ratio estimates is in Appendix S4.

**TABLE 1:** Comparison of estimated time and costs among 3 practical methods for assessing insect pollination services

	Desk‐based (low budget)	Field‐observation surveys (medium budget)	Empirical manipulations (high budget)
Estimated time required (based on 1 person doing the work)^a^	In the site and in the buffer^b^: Gather the data: 1 person‐day Carry out the desk‐based analysis: 1 person‐day Total (per crop): 2 person‐days Maximum time (assuming 5 crops): 10 person‐days	In the site: Visitation frequency per crop: 2–3 person‐days In the buffer: Visitation frequency per crop: 6 person‐days (2 days at each distance) Total (per crop): 8–9 person‐days Maximum time (assuming 5 crops): 45 person‐days	In the site: Make exclusion bags: 2 person‐days Bagging plants per crop: 1 person‐day Unbag and collect yield data: 2 person‐days In the buffer: Bagging plants per crop: 3 person‐days Remove bags: 1 person‐day Checking and adjusting bags in site and buffer: 2–3 person‐days Collect yield data: 2 person‐days Total (per crop): 13–20 person‐days Maximum time (assuming 5 crops): 60 person‐days
Estimated costs (per crop)	Materials: £0 Total estimate: £0	Materials: Pen, Paper: <£10 Total estimate: <£10	Materials: Mosquito net/gauze: up to £25–50 Plant labels and thread: £5 Sewing material: £10 Freezer bags: £10–20 (to store seeds/pods) Total estimate: ca £150
Notes	Although this is a desk‐based method, you may want to allow a day for a “ground truth” site visit.	Knowledge of crops and flowering time	‐ Costs will vary depending on the size of the plants and if you are bagging the whole plant or only 1 stem/branch ‐ Some crop types (e.g., perennial plants with branches) will require a more durable material (e.g., netting material that needs sealing), which will increase costs. ‐ Prices will also vary across countries. The time required for the experiment varies considerably depending on the crop type, flowering system, and habitat. We strongly recommend that you assess the specific logistical requirements of your crop before choosing this method, for example, working with tall trees might require help of professional tree climbers, and/or require more time between bagging and yield assessment.

^a^ Estimated time required for a task is based on the number of days 1 person needs to work on 1 type of crop (person‐day).

^b^ Area within 1‐km radius of the focal site.

### Low‐budget desk‐based method

The low‐budget method is the simplest. Time‐consuming and resource‐intensive field work is not needed (Figure 1 & Table 1). This desk‐based approach uses dependency ratios (proportion of yield due to animal pollination [Klein et al., 2007]) from databases and the estimated decay rate of pollinator visitation from peer‐reviewed scientific studies. It is therefore rapid and inexpensive. When there are no pollinator‐dependent crops growing within 1 km of the site, the value of pollination for the site itself is calculated using Equation (1) (Table 2) (method a1).

**TABLE 2:** Equations used to carry out practical methods for assessing insect pollination services provided by a site of conservation interest

Method	Equations	Worked example for oilseed rape at Noar Hill
**Desk‐based methods**		
Method a1 Value of pollination services at the focal site	Equation (1) $V_{\mathrm{site}}=\sum_{1}^{j}\left(Y\mathrm{ma}x_{j}\times DR_{j}\times P_{j}\times A_{j}\right)$ *Y*max*_i_* = maximum yield (ha^–1^∙year^–1^) for crop or wild good *i* DR*_i_* = dependency ratio of crop or wild good *i* *P_i_* = farmgate price of crop or wild good *i* (US$^−t^) *A_i_* = total area (ha) of crop or wild good *i* harvested at the site	No crops cultivated or wild goods harvested at reserve
Method a2 Value of pollination services within 1 km of the reserve (i.e., buffer)	Equation (2) $\mathrm{vf}\left(d\right)=a\times e^{\mu_{\beta }d}$ *a* = visitation frequency parameter at *d* = 0 m (i.e., at the perimeter of the focal site) where maximum visitation (*a* = 1) occurs *e* = inverse function of the natural logarithm (ln) *μ_β_* = decay rate = −0.00104, as specified in Appendix S3. The decay rate at −0.00118 or −0.00053 should be used instead if the site is in tropical or temperate climate domain, respectively.	a = 1 *d* = 59 m (innermost) *d* = 439 m (medium) *d* = 1000 m (outermost) $\;e^{-0.00104\;\times \;59}$ = 0.94 (innermost) $e^{-0.00104\;\times \;439}$ = 0.63 (medium) $e^{-0.00104\;\times \;1000}$ = 0.35 (outermost)
	Equation (3) $V_{\mathrm{buffer}\;}\;=\;\sum_{1}^{n}\;\left(\frac{\left(Y\mathrm{ma}x_{i}\times \;P_{i}\;\times \;DR_{i}\right)\;\times \;\text{vf}\left(d\right)}{a}\;-\;\frac{\left(Y\mathrm{ma}x_{i}\;\times \;P_{i}\;\times \;DR_{i}\right)\;\times \;\text{vf}\left(d=3000\right)}{a}\;\right)\;\times \;A_{i,\;n}$ *Y*max_i_ = maximum yield (t∙ha^–1^∙year^–1^) for crop *i* in buffer zone *n* vf(*d*) = visitation frequency parameter at distance, *d*, from the focal site *P_i_* = farmgate price of crop *i* ($^−t^) *a* = visitation frequency parameter at the focal site (*d* = 0 m) where visitation is at maximum level (*a* = 1) *A_i,n_* = total area (ha) of crop *i* within buffer zone *n* This equation includes the deduction of the estimated pollination value at 3000 m from the focal site in order to exclude the baseline pollination services provided by insect pollinators that persist in the agricultural matrix.	$Y\mathrm{ma}x_{j}=3.75$ $DR_{j}=0.25$ $P_{j}=\$423$ $A_{j}=43.1$ vf(d)= 0.94 (close) vf(d)= 0.63 (medium) vf(d)= 0.35 (far) $V_{\text{buffer}\;\;(\mathrm{inner})}$ = (3.75 × 423 × 0.25 × 0.94) − 83 = $289∙ha^–1^∙year^–1^ $V_{\text{buffer}\;\;(\mathrm{medium})}$= (3.75 × 423 × 0.25 × 0.63) − 83 = $166∙ha^–1^∙year^–1^ $V_{\text{buffer}\;\;(\mathrm{outer})}$ = (3.75 × 423 × 0.25 × 0.35) − 83 = $55∙ha^–1^∙year^–1^ $V_{\mathrm{buffer}\;(\mathrm{average})}$ = (289 + 166 + 55/3) = $170∙ha^–1^∙year^–1^ $\;V_{\mathrm{buffer}\;(\mathrm{total})}$ = (170 × 43.1) = $ 7327/year
Method a3 Value of pollination services in the alternative state	Equation (4) $V_{\mathrm{alternative}}\;=\;\sum_{1}^{i}\left(Y\mathrm{ma}x_{i}\;\times \;DR_{i}\;\times \;P_{i}\;\times \;A_{i}\right)\;\times \;e^{3000\mu \beta }$ *Y*max*_i_* = maximum yield (ha^–1^∙year^–1^) for crop *i* DR*_i_* = dependency ratio obtained from existing database for crop *i* *P_i_* = farmgate price of crop *i* ($^−t^) *A_i_* = total area (ha) of crop *i* harvested at the site under the alternative state *μ_β_* = overall decay rate for pollinator visitation (=−0.00104; see Appendix S3, but use value for tropics or temperate climate domain if appropriate)	$Y\mathrm{ma}x_{j}=3.75$ $DR_{j}=0.25$ $P_{j}=\$423$ $A_{j}=9.5$ $e^{1500\;\times \;-0.00104}$ = 0.21 (we used 1500 m instead of 3000 m for consistency with other methods) $V_{\mathrm{alternative}}$ = (3.75 × 0.25 × 423 × 0.21) = $83∙ha^–1^∙year^–1^ $V_{\text{alternative}\;\text{total}}$ = (83 × 9.5) = $788/year
**Field observation**		
Method b1 Value of pollination services at the focal site	Equation (5) $V_{\mathrm{site}}\;=\;\sum_{1}^{i}\left(Y\mathrm{ma}x_{i}\;\times \;DR_{i}\;\times \;P_{i}\;\times \;A_{i}\right)\;\times \;\text{vf}\left(d=0\right)\;$ vf(*d* = 0) = visitation frequency parameter at the focal site where *d* is the distance from the focal site = 0 (set at the default maximum value = 1) The rest of the parameters are the same as those in Equation (1). Fundamentally, Equation (5) provides the same estimate as Equation (1) from the desk‐based method.	No crops cultivated or wild goods harvested at reserve Maximum visitation set at same visitation frequency as innermost, which was recorder at boundaries of the site vf(d=0) = 0.0058
Method b2 Value of pollination services in a 1‐km buffer zone	Equation (6) $V_{\mathrm{buffer}\;}\;=\;\sum_{1}^{n}\;\left(\frac{\left(Y\mathrm{ma}x_{i}\times \;P_{i}\;\times \;DR_{i}\right)\;\times \;vf_{\mathrm{obs}}\left(d\right)}{vf_{\mathrm{obs}}\left(d=0\right)}\;-\;\frac{\left(\mathrm{Yma}x_{i}\;\times \;P_{i}\;\times \;DR_{i}\right)\;\times \;vf_{\mathrm{obs}}\left(d>1000\right)}{vf_{\mathrm{obs}}\left(d=0\right)}\;\right)\;\times \;A_{i,n}$ *Y*max*_j_* = maximum yield (t∙ha^–1^∙year^–1^) for crop *i* in buffer zone *n* vf_obs_(*d*) = observed visitation frequency at distance, *d*, from the focal site *P_j_* = farmgate price of crop *i* ($^−t^) *A_i,n_* = total area (ha) of crop, *i* in buffer zone *n* This equation—similar to Equation (3)—excludes baseline pollination services provided by those pollinators that persist in the agricultural matrix. This baseline pollination is estimated by using observed visitation frequency, vf_obs_(*d* > 1000) at a distance over 1000 m from the focal site.	$vf_{\mathrm{obs}}\left(d\right)$ = 0.0058 (innermost) $vf_{\mathrm{obs}}\left(d\right)$ = 0.0017 (medium) $vf_{\mathrm{obs}}\left(d\right)$ = 0.0016 (outermost) $V_{\text{buffer}\;\;(\mathrm{inner})}$= [(3.75 × 423 × 0.25 × 0.0058)/0.0058] − 82 = $314∙ha^–1^∙year^–1^ $V_{\mathrm{buffer}\;(\mathrm{medium})\;}$= [(3.75 × 423 × 0.25 × 0.0017)/0.0058] − 82 = $34∙ha^–1^∙year^–1^ $V_{\text{buffer}\;\;(\mathrm{outer})}$ = [(3.75 × 423 × 0.25 × 0.0016)/0.0058] − 82 = $27∙ha^–1^∙year^–1^ $V_{\mathrm{buffer}\;(\mathrm{average})}$ = (314 + 34 + 27/314) = $125∙ha^–1^∙year^–1^ $V_{\mathrm{buffer}(\mathrm{total})}$ = (125 × 43.1) = $5387/year
Method b3 Value of pollination services in the alternative state	Equation (7) $V_{\mathrm{alternative}}=\;\sum_{1}^{i}\;\left(\frac{\left(Y\mathrm{ma}x_{i}\times \;P_{i}\;\times \;DR_{i}\right)\;\times \;vf_{\mathrm{obs}}\left(d>1,000\right)}{vf_{\mathrm{obs}}\left(d=0\right)}\right)$ *Y*max*_i_* = maximum yield (t∙ha^–1^∙year^–1^) for crop *i* vf_obs_(*d*) = observed visitation frequency at distance, *d* (expressed in m), from the focal site *P_i_* = farmgate price of crop, *i* ($^−t^), *A_jn_* = total area (ha) of crop *i* in buffer zone *n*	$Y\mathrm{ma}x_{j}=3.75$ $DR_{j}=0.25$ $P_{j}=\$423$ $A_{j}=9.5$ $vf_{\mathrm{obs}}\left(d>1000\right)=0.0012$ (2000 m) $vf_{\mathrm{obs}}\left(d=0\right)=1$ $V_{\mathrm{alternative}}=\left(3.75\;\times \;423\;\times \;0.25\;\times \;0.0012/0.0058\right)=\;$$82∙ha^–1^∙year^–1^ $V_{\text{alternative}\;\text{total}}$ = (82 × 9.5) = $ 779/year
**Empirical manipulation**		
Method c1 Value of pollination services at the focal site	Equation (8) $V_{\mathrm{site}}\;=\;\sum_{1}^{i}\left(Y\mathrm{ma}x_{i}\;\times \;DR_{\mathrm{EE}\;i}\;\times \;P_{i}\;\times \;A_{i}\right)\;$ DR_EE_ *_i_* = dependency ratio obtained from the exclusion experiments for crop or wild good *i*. Parameters are the same as in Equation (1).	No crops cultivated or wild goods harvested at reserve
Method c2 Value of pollination services in a 1‐km buffer zone	Equation (9) $\begin{array}{c} V_{\mathrm{buffer}}\;=\;\sum_{1}^{n}\left(Y\mathrm{ma}x_{i}\;\times \;DR_{\mathrm{EE}\;i,\;n}\;\times \;P_{i}\;\times \;A_{i}\right)-\sum_{1}^{i}\\ (Y\mathrm{ma}x_{i}\;\times \;DR_{\mathrm{EE}\;i,\;d\;>1000}\;\times \;P_{i}\;\times \;A_{i})\; \end{array}$ DR_EE_ *_i_*_,_ *_n_* = dependency ratio obtained from the exclusion experiments for crop or wild good *i* in zone *n* DR_EE_ *_i, d_*_>1000_ = dependency ratio obtained from the exclusion experiments for crop or wild good *i* at a distance, *d*, over 1000 m from the focal site. The rest of the parameters are the same as in Equation (1). This equation—similar to Equations (3) and (6)—excludes baseline pollination services provided by those pollinators that persist in the agricultural matrix. This baseline pollination is estimated by using dependency ratio (DR_EE,_ *_i, d_*_>1000_) measured from a distance over 1000 m from the focal site (see method c2).	$Y\mathrm{ma}x_{j}=3.75$ $DR_{\mathrm{EE}\;i,\;n}=0.19$ $DR_{\mathrm{EE}\;i,\;d\;>1000}=0.10$ $P_{j}=\$423$ $A_{j}=43.1$ $\begin{array}{c} V_{\mathrm{buffer}}=\left(3.75\;\times \;0.19\;\times \;423\right)\\ -\;(3.75\;\times \;0.10\;\times \;423)=\; \end{array}$$143∙ha^–1^∙year^–1^ $V_{\text{buffer}\;\text{total}}=\left(142\;\times \;43.1\right)=\;$$6163/ha
Method c3 Value of pollination services in the alternative state	Equation (10) $V_{\mathrm{alternative}}\;=\;\sum_{1}^{i}\left(Y\mathrm{ma}x_{i}\;\times \;DR_{\mathrm{EE}\;i,\;d>1000}\;\times \;P_{i}\;\times \;A_{i}\right)\;$ DR_EE,_ *_i_*_,_ *_d_*_>1000_ = dependency ratio from the distance, *d*, over 1000 m from the focal site. The rest of the parameters are similar to those in Equation (1). For other types of alternative states, see guidance in method a3.	$Y\mathrm{ma}x_{j}=3.75$ $DR_{j}=0.10$ $P_{j}=\$423$ $A_{j}=43.1$ $V_{\mathrm{alternative}}=\left(3.75\;\times \;0.10\;\times \;423\right)=\;$$158∙ha^–1∙^year^–1^ $V_{\text{alternative}\;\text{total}}=\left(158\;\times \;9.5\right)=\;$$1501/ha

The rate many pollinators visit crop flowers decays with distance from seminatural habitat, giving rise to an estimated decay curve (Ricketts et al., 2008). To assess the value of pollination services to crops within 1 km of the site (buffer zone), one must establish what crops are growing in in this area and the distance of the crops from the site. The buffer is therefore divided into 3 distinct concentric zones, each approximately 300 m wide. The innermost zone is adjacent to the focal site and the outermost zone ≤1 km from the perimeter of the site. Visitation frequencies in each zone are calculated at the distance at which the crop occurs with Equation (2) (Table 2), which incorporates decay rate in pollinator visitation to crop flowers from Ricketts et al. (2008).

Visitation frequencies obtained from Equation (2) for each buffer zone are converted to a monetary value (U.S. dollars) with Equation (3) (Table 2). This includes the deduction of the estimated pollination value at 3 km, which is done to exclude the baseline pollination services provided by those pollinators that persist in the agricultural matrix independent of the pollination services provided by a natural or seminatural area (e.g., nature reserves) (method a2).

If the alternative state is agriculture that involves pollinator‐dependent crops, the pollination value for the focal site under the alternative state is calculated with Equation (4) (Table 2). Visitation frequency of insect pollinators for each important pollinator‐dependent crops or wild good is calculated at >1 km from the site as a measure of background pollination services attributed to the agricultural matrix (i.e., the alternative state). At this distance, one assumes that the site does not provide significant additional pollination services beyond those delivered by the agricultural landscape. If the focal site is degraded under the alternative state but retains its basic structure (e.g., logged forest), its total pollination value is the same as that of its current state (method a3).

### Medium‐budget field‐observation survey

The medium‐budget method is based on the existing data used in the desk‐based approach, but considers data obtained in field surveys. Such locally relevant and real‐time data on insect‐pollinator visitation frequency to crop and wild‐good flowers are used as a proxy for insect pollination services provided by the focal site.

When calculating the pollination services of crops growing inside the focal site, it is assumed pollination services are optimized and crops reach their highest yield. Thus, it is not necessary to collect visitation frequency data in the focal site. The value of pollination is calculated with Equation (5) (Table 2) (method b1).

When pollinator‐dependent crops are grown in the buffer zone, pollinator surveys should be carried out in the focal site (to establish a baseline visitation rate for optimal pollination services) and buffer zones to enable estimation of service decay (method b2).

In the focal site, for each important pollinator‐dependent crop or harvested wild goods, 9 (where possible) evenly distributed sampling locations are identified in the site, preferably at least 500 m apart, to increase the chances of independence of sampled flower visitors. At each sampling location, three 1 × 1 m plots are established (9 sampling locations and 27 plots for each crop type). The plot size is adapted to the target crop. All insects visiting crop flowers inside each plot are recorded for 15 min. Guidance on carrying out surveys, flower morphology, pollen vectors, and flower visitors is in the toolkit (Appendix S4). The number of open crop flowers in each plot is counted to determine visitation frequencies (number of visits per flower per minute). For each crop, average pollinator visitation frequency is calculated across the 9 plots in each buffer zone.

In the buffer, to determine the actual decay rate from the focal site, the area around that site is divided into 3 distinct zones as described in the desk‐based method. For each important pollinator‐dependent crop or wild good, 3 sampling locations within each buffer zone are randomly chosen. Where possible, these sampling locations avoid proximity to other natural or seminatural areas to minimize their influence. At each sampling location, three 1 × 1 m random plots are established (9 sampling locations and 27 plots across the distance gradient for each crop). The mean observed visitation frequency, vf (*d*), for each zone is obtained and converted to a monetary value with Equation (6) (Table 2, method b2).

To estimate the value of insect pollination services provided by the focal site under the alternative state, visitation frequency of insect pollinators for each pollinator‐dependent crop or wild good is collected >1 km from the site. If possible, data are collected 3 km from the focal site, which exceeds the average foraging range for the majority of bee species (Greenleaf et al., 2007). The pollination value for the site under the alternative state is calculated with Equation (7) (method b3).

### High‐budget empirical manipulation with exclusion experiments

We consider pollinator exclusion techniques the most robust means of estimating pollination services. Using this method, nonspecialists can directly derive the actual dependency ratios of the crops and wild goods at a site in its current and alternative states and those in the buffer.

For each pollinator‐dependent crop and wild good at the site, 15 pairs of plants at similar preflowering stage are randomly selected at the site for the exclusion experiment to estimate yield and pollination dependency ratio. Each pair is randomly assigned to floral units manipulated by being enclosed in mesh bags to prevent access by insect pollinators or unmanipulated floral units where flowers are accessible to wind and insect pollination (control). If resources do not permit use of whole plants, on each of 15 plants, 2 floral units (flower or inflorescence) at similar preflowering stage are selected and assigned to either bagged or control treatments.

At harvest, the yield of seeds or fruit is quantified for both treatments. The yield of bagged flowers is divided by the yield of unbagged flowers—the resulting ratio is the estimate of the proportion of yield due to wind and autopollination. The dependency ratio (DR), 1 – proportion of yield due to wind and autopollination, is calculated for each plant. These values are averaged to obtain a pollination contribution value for each crop in the site.

When there are no pollinator‐dependent crops growing in the buffer, the average dependency ratio (obtained from the 15 pairs of plants) is used to estimate the value of pollination services provided by the site with Equation (8) (method c1, Table 2).

When there are pollinator‐dependent crops growing in the buffer, exclusion experiments are repeated in each zone with 5 pairs of randomly chosen preflowering plants of each pollinator‐dependent crop or wild good. The average DR from all plants across the buffer is used to estimate the value of pollination services to crops and wild goods in the buffer with Equation (9) (Table 2, method c2).

For the alternative state, exclusion experiments are conducted outside the buffer (methods a3, b3) to determine the DR of each crop and wild good. The DR is used to estimate the pollination value of the site under the alternative state with Equation (10) (Table 2, method c3).

### Application

We applied the 3 methods to a 63‐ha nature reserve in the United Kingdom, Noar Hill. We used the methods to quantify the economic value of insect pollination services the reserve provides to the adjacent agricultural crops. Noar Hill (hereafter reserve) (Appendix S5) is primarily calcareous grassland (19.5 ha) and broadleaf woodland (43.5 ha). It is a site of special scientific interest. There are no crops cultivated or wild goods harvested at the reserve.

The hypothetical alternative state of the reserve is agricultural land. This alternative state reflects the same proportion of crop types occurring in the wider landscape, which in the focal site results in 18.9 ha of cereal (30%), 9.5 ha oilseed rape (15%), 9.5 ha field beans (15%), and 6.3 ha uncultivated land (10%).

In the agricultural land adjacent to the reserve, there were 2 insect‐pollinated crops: oilseed rape (*Brassica napus*) and field beans (*Vicia faba*). Both are grown widely as part of arable rotations in the region (Garratt, Breeze, et al., 2014). We interviewed (Appendix S6) with the local farmers to obtain information on the highest locally achievable yield in the area and cultivation locations. We used these data to estimate total area of each crop in the buffer; farmgate prices (market value minus selling costs); and annual production costs (costs attributable to crop production). Annual management costs for the reserve were obtained from the reserve manager at Hampshire and Isle of White Wildlife Trust.

For the desk‐based method, we derived the DR of each crop from the literature (Appendix S4) and estimated the pollinator visitation frequency parameter (Equation 2) along a distance gradient where both crops were cultivated (oilseed rape: 60, 440, 850, and 1500 m; field beans: 66, 388, 896, and 1500 m) based on published decay rate for pollinator visitation over distance (Table 2). Together with the information gathered from farmers, we estimated value of pollination services provided by the reserve in its current and alternative states (Equations 3 and 4, respectively). We converted monetary values from British pounds to U.S. dollars based on a 2017 exchange rate (£0.78 = US$1.00).

For the field surveys, we used methods b2 and b3 during peak flowering for both crops (17–21 April 2017 for oilseed rape and 19–23 June 2017 for field beans). We collected data on the visitation frequency of insect pollinators from 12 sampling locations and 36 plots (three 1 × 1 m plots at each sampling location) across the distance gradient from the reserve (i.e., 3 concentric buffer zones each 300 m wide and >1 km from the reserve as surrogate for the alternative state) for each crop. In each plot, all visits to flowers where visitors contacted the plant's reproductive parts were counted and all flower visitors were recorded for 15 min. We also counted the number of open flowers in each plot. We assumed crops in the buffer nearest to the reserve received the maximum visitation rate. Together with the DRs of the crops (oilseed rape, 0.25; field beans, 0.25) (Klein et al., 2007), we used observed visitation frequency data to estimate the value of insect pollination services to each crop provided by the reserve in its current and alternative states by applying Equations (6) and (7) (Table 2), respectively.

We set up the exclusion experiments for oilseed rape and field beans following methods c2 and c3. Sampling locations were approximately the same as for the field‐observation survey. Surveys were conducted from 26 March to 8 June 2017. For oilseed rape, 45 plants were placed across 3 concentric buffers (15 in each) and 45 plants were placed >1 km from the reserve (surrogate for alternative state). Each experimental plant had 1 bagged flower unit (raceme) and 1 unbagged raceme as control. For field beans, 90 plants were placed across 3 concentric buffer zones (15 bagged and 15 control in each zone) and 30 plants (15 bagged and 15 control plants) were placed >1 km from the reserve.

At harvest from 13 July to 5 August 2017, we counted the number of pods per stem and number of seeds per pod on oilseed rape‐treated racemes. We counted the number of pods per plant, number of beans per pod, and number of seeds per plant on field beans treated plants. For each plant of both crops, we measured dry seed weight and divided the yield of bagged flowers by the yield of unbagged flowers to estimate the proportion of yield from self‐pollination. The remaining proportion of yield (i.e., DR) was therefore attributed to insect pollination. For each crop, we obtained the mean DR for each buffer zone by averaging the values from all plants in each zone. We averaged the mean values from the zones to obtain a final DR for the entire 1‐km buffer. Likewise, we calculated mean DR of each crop outside the buffer. We used these mean DR values and Equations (9) and (10), respectively, to estimate the value of pollination services provided by the reserve in its current and alternative states (Table 2).

## RESULTS

The total area of oilseed rape and field beans growing in the 1‐km buffer around the reserve was 43.1 and 48.5 ha, respectively. Maximum locally achievable yields at the study site were 3.75 t∙ha^–1∙^year^–1^ for oilseed rape and 3.88 t∙ha^–1∙^year^–1^ for field beans. Farmgate prices for oilseed rape and field beans were $423 and $199/t, respectively. Farming cost was estimated at 1098∙ha^–1∙^year^–1^ for both crops and the management cost of the reserve was estimated at $6566/year.

### Desk‐based method

**FIGURE 2:**
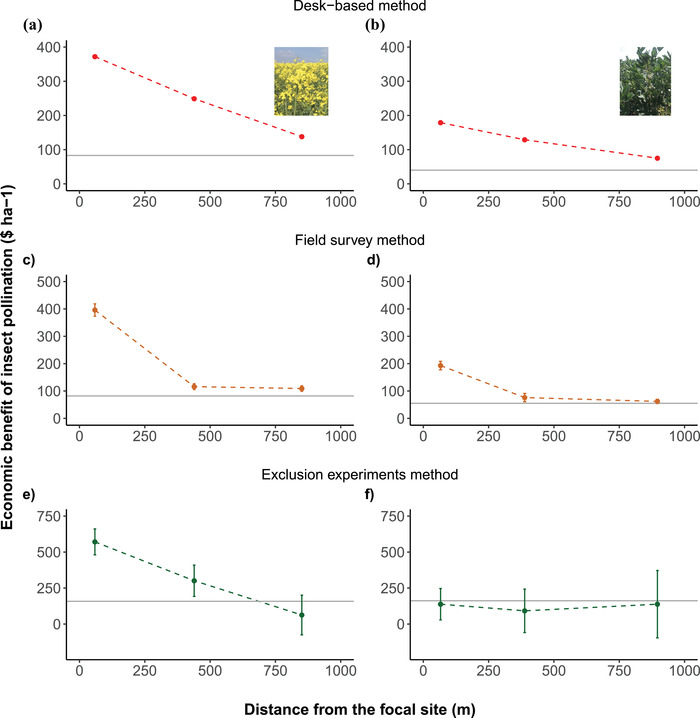
Economic values of pollination services (means SE) to (a, c, e) oilseed rape and (b, d, f) field beans at increasing distance from the reserve under the current state estimated with desk‐based method, field survey method, and exclusion experiments method (points, economic value of pollination services in the 3 areas described in Appendix S6 prior to deducting the baseline value [>1 km]; horizontal lines, value of pollination provided by pollinators that inhabit agricultural matrix [i.e., baseline pollination])

The exponential decay curve, based on the desk‐based assessment, showed a decrease in the value of insect pollination services to oilseed rape from the innermost buffer (nearest to the reserve) to the outermost buffer (Figure 2a). The value of baseline insect pollination for oilseed rape (derived at 1.5 km from the reserve for consistency with the other methods)—which equates to the value of insect pollination services to this crop provided by the reserve under its alternative state—was estimated at $83∙ha^–1^∙year^–1^ ($788/year) (Figure 2a & Table 2). After deducting this baseline value, the additional value of insect pollination services attributed to the reserve ranged from $289∙ha^–1^∙year^–1^ in the innermost buffer to $55∙ha^–1^∙year^–1^ in the outermost buffer, with an estimated average value of $170∙ha^–1^∙year^–1^.

Similarly, insect pollination services for field beans declined along the distance gradient from the reserve (Figure 2b). After deducting the baseline value estimated of $40∙ha^–1^∙year^–1^ ($380/year) (equal to the alternative state), the additional value of insect pollination services to field beans provided by the reserve ranged from $139∙ha^–1^∙year^–1^ in the innermost buffer to $35∙ha^–1^∙year^–1^ in the outermost buffer, for an average value of $87∙ha^–1^∙year^–1^ (Figure 2b). Total value of insect pollination services to both crops provided by the reserve under the current and alternative state was estimated at $257 and $123∙ha^–1^∙year^–1^, respectively (Table 3).

**TABLE 3:** Estimated value of insect pollination services provided to oilseed rape and field beans by the reserve under the current and alternative states

	Current state						Alternative state					
	Desk based		Method field survey		Exclusion experiment		Desk based		Method field survey		Exclusion experiment	
	Value ($∙ha^–1^∙year^–1^)	Overall value ($/year)	Value ($∙ha^–1^∙year^–1^)	Overall value ($/year)	Value ($∙ha^–1^∙year^–1^)	Overall value ($/year)	Value ($∙ha^–1^∙year^–1^)	Overall value ($/year)	Value ($∙ha^–1^∙year^–1^)	Overall value ($/year)	Value ($∙ha^–1^∙year^–1^)	Overall value ($/year)
Oilseed rape	170	7327	125	5387	143	6163	83	788	82	779	158	1501
Field beans	87	4219	56	2716	0	0	40	380	55	522	162	1539
Total annual benefit	257	11,546	181	8103	143	6163	123	1168	137	1301	320	3040
Total annual benefit of conservation		10,378		6802		3123						

*Note*: For the current state, the overall values of insect pollination services provided by the reserve to each insect‐dependent crop was obtained by multiplying the values of pollination services per hectare by the total area of the crop (oilseed rape: 43.1 ha; and field beans: 48.5 ha) in the 1‐km wide buffer around the reserve. The alternative state of the reserve reflects the same proportion of crop types occurring in the wider landscape (approximately cereal, 30% [18.9 ha]; oilseed rape, 15% [9.5 ha]; field beans, 15% [9.5 ha]; and uncultivated land, 10% [6.3 ha]). To derive the overall values of insect pollination services of each insect‐dependent crop under the alternative state, the values of pollination services per hectare were multiplied by 9.5 ha. Annual pollination benefit due to the protection status is the difference in the total values between the 2 states of the reserve.

### Field survey

The observed visitation frequency of insect pollinators to flowers of oilseed rape declined sharply along the buffer (nearest to the reserve 0.0058 visits∙flower^–1^∙min^–1^: outermost buffer 0.0016 visits∙flower^–1^∙min^–1^). Based on the observed visitation frequency and dependency ratio of oilseed rape (0.25; Klein et al., 2007), and after deducting the baseline value of insect pollination (estimated at $82∙ha^–1^∙year^–1^, $779/year also equal to value of the alternative state of the reserve), the additional insect pollination value provided by the reserve ranged from $314∙ha^–1^∙year^–1^ in the buffer nearest to the reserve to $27∙ha^–1^∙year^–1^ in the outermost buffer (Figure 2c). The average estimated value estimated was $125∙ha^–1^∙year^–1^.

The observed visitation frequency of insect pollinator to field bean flowers declined from 0.0028 visits∙flower^–1^∙min^–1^ in the buffer nearest to the reserve to 0.0009 visits∙flower^–1^∙min^–1^ in the outermost buffer, dropping by half at approximately 500 m from the reserve (Figure 2d). After deducting the baseline value (estimated at $55∙ha^–1^∙year^–1^, $525/year, which also equated to the value of insect pollination services provided by the alternative state), the value of additional pollination services provided by the reserve ranged from $138∙ha^–1^∙year^–1^ in the innermost buffer to $7∙ha^–1^∙year^–1^ in the outermost buffer, giving an average estimate of $56∙ha^–1^∙year^–1^. Total value of insect pollination services to both crops provided by the reserve under the current and alternative state was estimated at $181 and $137∙ha^–1^∙year^–1^, respectively (Table 3).

### Empirical manipulation with exclusion experiment

The relative contribution of insects to pollination of oilseed rape (DR) decreased from 0.36 in the buffer nearest to the reserve to 0.04 in the buffer furthest from the reserve (Figure 2e). Hence, the mean DR of oilseed rape was 0.19. After deducting the baseline value, the value of additional pollination services for oilseed rape production within the 1‐km buffer from the reserve was estimated at $143∙ha^–1^∙year^–1^. Beyond 1 km from the reserve, the mean DR of oilseed rape was 0.10. The value of pollination services under the alternative state is the baseline value of $158∙ha^–1^∙year^–1^.

The mean DR of field beans ranged between 0.12 and 0.18 within the 1‐km buffer from the reserve with no clear decay curve with distance from the site (Figure 2f). Beyond 1 km from the reserve, the mean DR for field beans was 0.21, with an estimated pollination value of $162∙ha^–1^∙year^–1^. This indicated that there was no pollination service for field beans provided by the reserve (Table 3). Total value of insect pollination services to both crops provided by the reserve under the current and alternative state was estimated at $143 and $320∙ha^–1^∙year^–1^, respectively (Table 3).

Overall, we estimated that the economic value of pollination services provided by the reserve (to the crops outside the reserve) ranged from $6163 to $11,546/year, depending on the method adopted (Table 3). Our results showed that the conversion of the reserve to arable land would provide no insect pollination services to the adjacent cropland. However, this alternative state of the reserve would have a net annual benefit from insect‐pollinated crop production estimated at $1542/year ($24∙ha^–1^∙year^–1^) (Table 4).

**TABLE 4:** The insect pollination service values and management costs from the reserve (63 ha) and of the same land if the reserve were converted into arable land (63 ha)

	Nature reserve (63 ha)	Arable land (63 ha)
Service (flow) ($/year)		
Insect‐pollinated crop production	0	22,404
Insect pollination	6120^a^	3040^b^
Management cost ($/year)	6566	20,862
Net annual benefit ($/year)	–446	1542
Net annual benefit ($∙ha^–1^∙year^–1^)	–7	24

^a^ Value of insect pollination services to the crops cultivated in a 1‐km area around the reserve.

^b^ Value of pollination services to crops cultivated in the area of the reserve under the alternative state. This value is a composite value of crop production and therefore not included in the net annual benefit.

## DISCUSSION

The methods we tested aim to enable nonspecialists with limited expertise and resources to estimate the value of insect pollination services provided by a site. The 3 methods were practical and effective and provided a comparison between the estimate of the insect pollination services provided by a site of conservation interest and that provided by the same area under an alternative state. For oilseed rape, the estimates of the insect pollination value to the crops outside the reserve based on the most robust exclusion experiment method were lower than the estimate obtained from the desk‐based method by 16% and greater than the estimate obtained from field survey methods by 12%. The estimate of the insect pollination service value provided by the agricultural matrix in the exclusion experiment method was almost double that of the other 2 methods. For field beans, the exclusion experiments showed that the reserve did not provide any insect pollination services. Hence, the desk‐based and field survey methods overestimated the insect pollination services to the crop outside the reserve by 87% and 56%, respectively. The value of insect pollination provided by the agricultural matrix was again underestimated by the desk‐based and field survey methods by 75% and 66%, respectively. The 3 methods varied in degree of accuracy (Table 2), showing that there is a trade‐off between simplicity (associated with speed and low cost) and accuracy.

A number of assumptions underpinning each method present limitations. The simpler methods had more associated assumptions, which may present limitations. The desk‐based method, which uses the best available published estimate of distance decay rate (Ricketts et al., 2008), inevitably generalized the relationship between pollinator visitation frequency and distance from natural habitat, providing a less accurate estimate of the value of pollination to yield of a focal crop. In fact, the field survey methods in our application detected a steeper distance decay rate for both crops than that found in Ricketts (2008). The effect of distance on crop flower visitation frequency depends on the crop's key pollinators and their foraging ranges, which vary among taxonomic groups and body sizes (Greenleaf et al., 2007). The estimate would have a higher level of confidence if dependency ratios (from existing databases) were derived from similar habitat near the focal site. The dependency ratios we used could be derived from areas that do not provide a good surrogate of the Noar Hill site or from moderately different crops and wild goods (i.e., different varieties). Users could increase accuracy by using local pollinator visitation data where available and adjusting the buffer radius based on information on the mobility of known crop visitors. Furthermore, variety‐specific values of DR should be used if available to reduce the varietal differences observed for some crops (Bishop et al., 2020; Stanley et al., 2013). If the varieties planted in the area vary between years, it may still be appropriate to use an average for that particular crop species.

The field survey method in field beans produced an overestimation of the pollination service value compared with the exclusion experiment method. The effectiveness of visitation rate as a proxy for pollination services is dependent on the crop, taxa of visiting insects (Andrikopoulos & Cane, 2018) and their behavior (Monzón et al., 2004), and the frequency of visits with, in some circumstances, very high visitation rates even leading to a reduction in crop yield (Sáez et al., 2014). The overestimation by this method might be due to robbing bumblebee species, which are the predominant flower visitors of field beans (Garratt, Coston, et al., 2014), being counted as legitimate visitors. Also, there could be considerable variation in pollinator visitation rate between days, seasons, and years, which could potentially affect the results of field surveys with 1‐day observations (Fijen & Kleijn, 2017). To improve accuracy, the sampling effort could be increased (e.g., repeating observation across 3 or more days), visitation time could be reduced, or sampling points increased to account for variation in visitation within the crop to provide a more robust estimate of visitation rate. Furthermore, using visitation rate as a proxy assumes equal effectiveness of all pollinators; thus, users may consider using only the visitation rate of the most effective pollinators for a given crop, if this information is known.

The exclusion experiment method provides the most accurate measure of the contribution of insects to crop pollination. Nonscientists can be trained to use this method (Garratt et al., 2019). However, the time required to carry out exclusion experiments varies considerably among crop types. A skilled researcher in this study who implemented this method used 20 and 13 person‐days for oilseed rape and field beans, respectively. This resource requirement may challenge the rapid assessment framework, especially when there are several insect‐pollinated crops grown at the site of interest and in its adjacent buffer. Furthermore, the time delay between bagging and the actual measurement of pollination service contribution may be a limiting factor in adopting this method. Nevertheless, where resources are limited, users could adapt this method to their circumstances, for example, by focusing on the few crops that are most relevant to the local economy or crops with the highest dependency on insect pollination.

The results of our field application showed that the conversion to arable land would be economically more profitable than maintaining the site in its current state due to the maintenance costs exceeding the economic benefits of pollination services. Indeed, an economic valuation of pollination to crop alone does not capture the intrinsic and aesthetic values attached to pollinators existence. Furthermore, less‐dominant pollinator species that do not contribute substantially to crop pollination provide a stabilizing effect on the services over time or space and resilience in the face of environmental change. This highlights the importance of applying an integrated ecosystem service approach when assessing the value of a protected site to provide a more holistic estimate of its value and more robust argument for site conservation. Furthermore, the benefits and cost calculated are not equally distributed among stakeholder because some may benefit from conversion to agriculture (e.g., farmers) and others from site conservation (e.g., recreational users). Our methods can potentially reveal the synergies and trade‐offs that may provide insight into ensuring an equitable distribution of benefits and costs while conserving biodiversity.

Our practical methods necessarily simplify some facets of pollination service provision. Estimating whether the yield of a crop or wild good is under‐ versus overpollination is beyond the scope of our approach (Garibaldi et al., 2020) and, in most modified landscapes, underpollination is likely the norm (Reilly et al., 2020). Nevertheless, our methods could be a useful addition to a range of existing pollination service assessment and monitoring tools. Other computer‐based approaches (e.g., InVEST pollination model) can be unsuitable in many developing parts of the world, where there is a lack of locally relevant data and technical expertise. These are also the poorer areas where there is a heavy reliance on locally grown produce and yet insect pollinators are threatened due to habitat loss or degradation (Ashworth et al., 2009). We found that our methods can be implemented readily by nonexperts; enable low‐cost comparative assessment of a protected area to illustrate the economic consequences of loss of insect pollination services provided by the area; and yield straightforward results that can be easily interpreted to inform decision‐making or management.

## Supporting information

Supplementary material

## References

[cobi13886-bib-0001] Andrikopoulos, C. , & Cane, J. (2018). Comparative pollination efficacies of five bee species on raspberry. Journal of Economic Entomology, 111(6), 2513–2519. 30137356 10.1093/jee/toy226

[cobi13886-bib-0002] Ashworth, L. , Quesada, M. , Casas, A. , Aguilar, R. , & Oyama, K. (2009). Pollinator‐dependent food production in Mexico. Biological Conservation, 142, 1050–1057.

[cobi13886-bib-0003] Bishop, J. , Garratt, M. P. D. , & Breeze, T. D. (2020). Yield benefits of additional pollination to faba bean vary with cultivar, scale, yield parameter and experimental method. Scientific Reports, 10, 1–11 32034193 10.1038/s41598-020-58518-1 PMC7005869

[cobi13886-bib-0004] Dainese, M. , Martin, E. A. , Aizen, M. A. , Albrecht, M. , & Bartomeus, I. (2019). A global synthesis reveals biodiversity‐mediated benefits for crop production. Science Advances, 5, eaax0121. 31663019 10.1126/sciadv.aax0121 PMC6795509

[cobi13886-bib-0005] Fijen, T. P. M. , & Kleijn, D. (2017). How to efficiently obtain accurate estimates of flower visitation rates by pollinators. Basic and Applied Ecology, 19, 11–18.

[cobi13886-bib-0006] Garibaldi, L. A. , Steffan‐Dewenter, I. , Kremen, C. , Morales, J. M. , Bommarco, R. , Cunningham, S. A. , Carvalheiro, L. G. , Chacoff, N. P. , Dudenhöffer, J. H. , Greenleaf, S. S. , Holzschuh, A. , Isaacs, R. , Krewenka, K. , Mandelik, Y. , Mayfield, M. M. , Morandin, L. A. , Potts, S. G. , Ricketts, T. H. , Szentgyörgyi, H. , … Winfree, R . (2011). Stability of pollination services decreases with isolation from natural areas despite honey bee visits. Ecology Letters, 14, 1062–1072. 21806746 10.1111/j.1461-0248.2011.01669.x

[cobi13886-bib-0007] Garibaldi, L. A. , Sáez, A. , Aizen, M. A. , Fijen, T. , & Bartomeus, I. (2020). Crop pollination management needs flower visitor monitoring and target values. Journal of Applied Ecology, 57, 664–670.

[cobi13886-bib-0008] Garratt, M. P. D. , Breeze, T. D. , Jenner, N. , Polce, C. , Biesmeijer, J. C. , & Potts, S. G. (2014). Avoiding a bad apple: Insect pollination enhances fruit quality and economic value. Agriculture Ecosystems & Environment, 184, 34–40. 10.1016/j.agee.2013.10.032 PMC3990452 24748698

[cobi13886-bib-0009] Garratt, M. P. D. , Coston, D. J. , Truslove, C. L. , Lappage, M. G. , Polce, C. , Dean, R. , Biesmeijer, J. C. , & Potts, S. G. (2014). The identity of crop pollinators helps target conservation for improved ecosystem services. Biological Conservation, 169, 128–135. 24696525 10.1016/j.biocon.2013.11.001 PMC3969722

[cobi13886-bib-0010] Garratt, M. P. D. , Potts, S. G. , Banks, G. , Hawes, C. , Breeze, T. D. , O'Connor, R. S. , & Carvell, C. (2019). Capacity and willingness of farmers and citizen scientists to monitor crop pollinators and pollination services. Global Ecology and Conservation, 20, e00781.

[cobi13886-bib-0011] Greenleaf, S. S. , Williams, N. M. , Winfree, R. , & Kremen, C. (2007). Bee foraging ranges and their relationship to body size. Oecologia, 153, 589–596. 17483965 10.1007/s00442-007-0752-9

[cobi13886-bib-0012] Kennedy, C. M. , Lonsdorf, E. , Neel, M. C. , Williams, N. M. , Ricketts, T. H. , Winfree, R. , Bommarco, R. , Brittain, C. , Burley, A. L. , Cariveau, D. , Carvalheiro, L. G. , Chacoff, N. P. , Cunningham, S. A. , Danforth, B. N. , Dudenhöffer, J. H. , Elle, E. , Gaines, H. R. , Garibaldi, L. A. , Gratton, C. , & Holzschuh, A . (2013). A global quantitative synthesis of local and landscape effects on wild bee pollinators in agroecosystems. Ecology Letters, 16, 584–599. 23489285 10.1111/ele.12082

[cobi13886-bib-0013] Klein, A.‐M. , Vaissière, B. E. , Cane, J. H. , Steffan‐Dewenter, I. , Cunningham, S. A. , Kremen, C. , & Tscharntke, T. (2007). Importance of pollinators in changing landscapes for world crops. Proceedings of the Royal Society B: Biological Sciences, 274, 66, 95–96, 191. 10.1098/rspb.2006.3721 PMC1702377 17164193

[cobi13886-bib-0014] Monzón, V. H. , Bosch, J. , & Retana, J. (2004). Foraging behavior and pollinating effectiveness of *Osmia cornuta* (Hymenoptera: Megachilidae) and *Apis mellifera* (Hymenoptera: Apidae) on “Comice” pear. Apidologie, 35, 575–585.

[cobi13886-bib-0015] Neugarten, R. A. , Langhammer, P. F. , Osipova, E. , Bagstad, K. J. , Bhagabati, N. , Butchart, S. H. M. , Dudley, N. , Elliott, V. , Gerber, L. R. , Gutierrez Arrellano, C. , Ivanić, K.‐Z. , Kettunen, M. , Mandle, L. , Merriman, J. C. , Mulligan, M. , Peh, K. S.‐H. , Raudsepp‐Hearne, C. , Semmens, D. J. , Stolton, S. , … Groves, C. (2018). Tools for measuring, modelling, and valuing ecosystem services provided by Key Biodiversity Areas, natural World Heritage sites, and protected areas. IUCN.

[cobi13886-bib-0016] Ollerton, J. (2017). Pollinator diversity: Distribution, ecological function, and conservation. Annual Review of Ecology, Evolution, and Systematics, 48, 353–376.

[cobi13886-bib-0017] Peh, K. S.‐H. , Balmford, A. , Bradbury, R. B. , Brown, C. , Butchart, S. H. M. , Hughes, F. M. R. , Stattersfield, A. , Thomas, D. H. L. , Walpole, M. , Bayliss, J. , Gowing, D. , Jones, J. P. G. , Lewis, S. L. , Mulligan, M. , Pandeya, B. , Stratford, C. , Thompson, J. R. , Turner, K. , Vira, B. , … Birch, J. C. (2013). TESSA: A toolkit for rapid assessment of ecosystem services at sites of biodiversity conservation importance. Ecosystem Services, 5, 51–57.

[cobi13886-bib-0018] Peh, K. S.‐H. , Balmford, A. P. , Bradbury, R. B. , Brown, C. , Butchart, S. H. M. , Hughes, F. M. R. , MacDonald, M. A. , Stattersfield, A. J. , Thomas, D. H. L. , Trevelyan, R. J. , Walpole, M. , & Merriman, J. C. (2017). Toolkit for Ecosystem Service Site‐based Assessment (TESSA) . Version 2.0.

[cobi13886-bib-0019] Potts, S. G. , Imperatriz‐Fonseca, V. , Ngo, H. T. , Aizen, M. A. , Biesmeijer, J. C. , Breeze, T. D. , Dicks, L. V. , Garibaldi, L. A. , Hill, R. , Settele, J. , & Vanbergen, A. J . (2016). The assessment report of the intergovernmental science‐policy platform on biodiversity and ecosystem services on pollinators, pollination and food production. Nature, 540, 220–229. 27894123 10.1038/nature20588

[cobi13886-bib-0020] Reilly, J. R. , Artz, D. R. , Biddinger, D. , Bobiwash, K. , Boyle, N. K. , Brittain, C. , Brokaw, J. , Campbell, J. W. , Daniels, J. , Elle, E. , Ellis, J. D. , Fleischer, S. J. , Gibbs, J. , Gillespie, R. L. , Gundersen, K. B. , Gut, L. , Hoffman, G. , Joshi, N. , Lundin, O. , Mason, K. , & McGrady, C. M . (2020). Crop production in the USA is frequently limited by a lack of pollinators. Proceedings of the Royal Society B: Biological Sciences, 287, 20200922. 10.1098/rspb.2020.0922 PMC7423660 33043867

[cobi13886-bib-0021] Ricketts, T. H. , Regetz, J. , Steffan‐Dewenter, I. , Cunningham, S. A. , Kremen, C. , Bogdanski, A. , Gemmill‐Herren, B. , Greenleaf, S. S. , Klein, A. M. , Mayfield, M. M. , Morandin, L. A. , Ochieng', A. , Potts, S. G. , & Viana, B. F . (2008). Landscape effects on crop pollination services: Are there general patterns? Ecology Letters, 11, 499–515. 18294214 10.1111/j.1461-0248.2008.01157.x

[cobi13886-bib-0022] Ricketts, T. H. , & Lonsdorf, E. (2013). Mapping the margin: Comparing marginal values of tropical forest remnants for pollination services. Ecological Applications, 23, 1113–1123. 23967579 10.1890/12-1600.1

[cobi13886-bib-0023] Sáez, A. , Morales, C. L. , Ramos, L. Y. , & Aizen, M. A. (2014). Extremely frequent bee visits increase pollen deposition but reduce drupelet set in raspberry. Journal of Applied Ecology, 51, 1603–1612.

[cobi13886-bib-0024] Stanley, D. A. , Gunning, D. , & Stout, J. C. (2013). Pollinators and pollination of oilseed rape crops (*Brassica napus* L.) in Ireland: Ecological and economic incentives for pollinator conservation. Journal of Insect Conservation, 17, 1181–1189.

