# Supplementary material for: Rapid assessment of insect pollination services to inform decision‐making
Source: Conserv Biol. 2022 Mar 8;36(4):e13886. doi: 10.1111/cobi.13886 (PMC9542742; doi:10.1111/cobi.13886)
Supplement: Supplementary file 1 — Supplementary material [file COBI-36-0-s001.docx]

## Practical methods for assessing insect pollination services provided by a site of conservation interest

- 1. Background to the Toolkit for Ecosystem Service Site-based Assessment (TESSA) project

Assessing ecosystem services can support advocacy for the conservation of individual sites (e.g., when they are under threat of some form of conversion or development) or for their restoration (e.g., rehabilitating logged forest or polluted or drained wetlands). The objective of the Toolkit for Ecosystem Service Site-based Assessment (TESSA) project is to develop a suite of rapid ecosystem service assessment tools for understanding how far conserving sites for their biodiversity importance also helps to provide different ecosystem services, relative to a converted state (Peh et al. 2013).

TESSA aims to provide practical guidance on how to identify which ecosystem services may be important at a site, and the methods for rapidly measuring some of these for the current state of the site compared to its most plausible alternative (converted) state. The toolkit is aimed at conservation professionals, project or site managers, technical field officers and students. In using this toolkit, it is expected that these people would then provide simple and focused instructions to staff and volunteers on how to collect or collate the data needed to measure the service(s) at individual sites. The aspiration is that the toolkit can provide approximate service estimates that are robust enough for effective advocacy, without necessitating investment of considerable resources or requiring specialist technical knowledge.

For TESSA Version 2.0, we have developed a module to guide users step-by-step through a practical assessment of insect pollination services. In this study, we tested the methods of this module at Noar Hill, UK. Besides pollination services, the toolkit also contains a wide range of other ecosystem service modules:

- global climate regulation (including carbon storage and greenhouse gas fluxes)
- water-related services (flood protection, water provision, water-quality improvement)
- nature-based recreation and tourism
- wild goods (bush meat, etc.)
- cultivated goods (crops, livestock, etc.)
- coastal protection services
- cultural ecosystem services

These modules provide accessible protocols on how to collect data to measure the ecosystem services. The toolkit also emphasises the importance of conducting the same assessment for the most likely alternative state of the site, e.g., after conversion, so that the net consequences of land use change in terms of costs and benefits can be calculated. For guidance on how to determine the alternative state of the site of interest with stakeholders, refer to TESSA (Peh et al. 2017) which can be download at: <http://tessa.tools/>.

**REFERENCE**

Peh KS-H et al. 2013. TESSA: A toolkit for rapid assessment of ecosystem services at sites of biodiversity conservation importance. Ecosystem Services **5**:51–57.

Peh KS-H et al. 2017. Toolkit for Ecosystem Service Site-based Assessment (TESSA), 2nd edition. Cambridge, UK. Available from file:///Files/38/385b03a4-643c-42ee-8c6b-2f2f01aa16e1.pdf.

- 1. Expert workshop for developing practical methods to assess insect pollination services from a site of conservation interest

The development of the practical methods of valuing insect pollination services for the Toolkit for Ecosystem Services Site-based Assessment (TESSA; for information about TESSA project, see http://tessa.tools/) was conducted during a two-day workshop held at the UNEP-World Conservation Monitoring Centre, Cambridge on 19^th^ and 20^th^ September 2016. The participants were:

- Dr Tom Breeze, University of Reading (Post-doctoral researcher)
- Dr Lorna Cole, SRUC, Scotland Rural College (Lecturer)
- Dr Lynn V. Dicks, University of East Anglia (Lecturer)
- Dr Mike Garratt, University of Reading (Lecturer)
- Professor Bill Kunin, University of Leeds (Professor)
- Dr Denis Michez, Université De Mons, Belgium (Associate Professor)
- Dr Rory O’Connor, University of Reading (Post-doctoral researcher)
- Professor Jeff Ollerton, University of Northampton (Professor)
- Professor Robert J. Paxton, Martin Luther Universität, Germany (Professor)
- Professor Simon G. Potts, University of Reading (Professor)
- Dr Deepa Senapathi, University of Reading (Post-doctoral researcher)
- Dr Rosalind Shaw, University of Exeter (Post-doctoral researcher)

And the facilitators were:

- Dr Kelvin S.-H. Peh, University of Southampton (Lecturer)
- Fabrizia Ratto, University of Southampton (Doctoral researcher).

The workshop could not invite pollination experts outside Europe due to the budget constraints. Nevertheless, many participants have research experience in the tropics and other geographical regions. To improve the overall quality of expert judgment, the workshop included a mix of highly established experts (i.e., professors) and early career researchers (post-doctoral researchers and lecturers). The workshop was conducted as a facilitator-lead, interactive process consisting of alternating plenary and breakout sessions (TableS2-1). Participants were organised into three working groups, with diverse expertise included in each group.

**Table S2-1**: Pollination service assessment workshop programme.

| Workshop activities | Details |
| --- | --- |
| DAY 1 |  |
| Plenary session 1 | Presentation by Kelvin Peh - “An introduction to the project”. |
| Plenary session 2 | Presentation by Fabrizia Ratto – “Setting the context”. |
| Open Forum | Open discussion to answers the following questions:   - What do we measure? - Are the aims of the project clear? - How feasible is the project? - Any missing points? |
| Breakout session 1 | Catalogue all the possible metrics and approaches:   - Biometrics - Habitat and landscape metrics - Economic   The criteria for suitability:   - Local relevance - Requirements for expertise - Prior data - Time and costs   Potential alternative state |
| Breakout session 2 | Structure the up-front decision tree   - Note this has to be accessible, fit for purpose for valuing, monitoring, etc. - More than one method? The decision tree may lead to different protocols   Discuss approaches and key issues   - Use existing toolkit as template   Resources needed   - Any look-up tables, existing databases |
| DAY 2 |  |
| Plenary Session 3 | Structuring the initial decision trees |
| Breakout session 3 | Structuring the three step-by-step methods |
| Plenary Session 4 | Group discussion on the next steps:   - Potential sites for piloting the pollination protocol in 2017 - Measurement of synergies across services in the toolkit - Potential double counting - Uncertainties - Next step: publications in which journal? |

- 1. Practical methods for assessing insect pollination services provided by a site of conservation interest

A detailed description of three practical methods for assessing pollination services provided by a site of conservation interest:

a) **Desk-based method** (low budget)

Method a1: Estimating the value of insect pollination services to crops and wild goods cultivated or harvested at the focal site, using existing datasets

Method a2: Estimating the value of insect pollination services to crops and wild goods cultivated or harvested within a buffer of 1-km wide around the focal, using existing datasets

Method a3: Estimating the value of insect pollination services to crops and wild goods cultivated or harvested at the focal site under the alternative state, using a desk-based method

b) **Field observation survey** (medium budget)

Method b1: Estimating the value of insect pollination services provided by the focal site, based on observed visitation rate of insect pollinators as a proxy for pollination

Method b2: Estimating the value of insect pollination services provided by the focal site under the alternative state, based on observed visitation rate of insect pollinators as a proxy for pollination

c) **Empirical manipulation using exclusion experiment** (high budget)

Method c1: Estimating the value of insect pollination services provided by the focal site, using exclusion experiments

Method c2: Estimating the value of insect pollination services provided by the focal site under the alternative state, using exclusion experiments

**Practical methods for assessing pollination services provided by a site of conservation interest**

Recognition of the value of the natural systems and the extent to which we are all vitally dependent on the benefits provided by them has led to a greater effort to protect the natural environment. Demonstrating the importance of pollinators by identifying the benefits that they provide to people can strengthen the argument for the conservation of their habitats. This document contains three methods – of different levels of complexity – and guidance on how to identify the most important insect-pollinated crops and wild goods, and how to measure the pollination services to these goods provided by a site of conservation interest. Data obtained from these methods may help us to understand the economic consequences of losing pollination services provided by the current state of individual sites and can have implications in planning and management decisions to support both pollinator conservation and the delivery of pollination services.

The methods presented here are designed for assessing the difference in pollination services between two alternative states of the site of interest: the current state and the counterfactual where a decision has resulted in an alternative land use at the site (i.e., the most plausible alternative state). For example, pollination services could be estimated for a natural site under current conservation protection compared with a future, theoretical scenario in which that same site is without protection and where expansion of agriculture has occurred. To make effective decisions, it is key to know the difference between the magnitude of pollination services – along with other ecosystem services – provided by a site in its current state compared to a plausible alternative one where the habitat is converted, or in which resources are unsustainably exploited. This gives the net benefit of the conservation state of the site rather than just its gross values (Peh et al., 2013). It is recommended that users of this guide refer to the Toolkit for Ecosystem Service Site-based Assessment (TESSA; Peh et al., 2017) on how to determine the alternative state of their site of interest and how to find a comparative site to represent the alternative state of the site.

The first step of a pollination service assessment is to identify the important crops and harvested wild goods found at the focal (i.e., assessment) site, as well as those found within a 1-km buffer around the site. Such information can be obtained by interviewing the stakeholders, particularly the producers, farmers and site managers. Information to gather from the stakeholders includes:

- A list of up to five key insect-pollinated crops/wild goods cultivated or harvested at the assessment site. These should be the most important goods for local livelihoods and should be determined at a stakeholder meeting.
- Total area (expressed in terms of ha) of each key insect-pollinated crop/wild good cultivated or harvested at the focal site. This information may also be available from land-use maps (e.g. Google Earth).
- Maximum yield (expressed in terms of tonnes ha^-1^ yr^-1^) achievable in the region for each key insect-pollinated crop/wild good.
- Dependency Ratio (DR) of each key insect-pollinated plants (for the estimates, see Appendix S4, Table S4-1). The methods using exclusion experiments (Methods c1 and c2) do not require DR estimates.
- Farmgate price (expressed in $ tonne^-1^) of each key insect-pollinated crop and commercial price for each key wild good. Farmgate price is defined as the market value of the product, minus the selling costs such as transport and marketing costs.

**a) Desk-based methods (low budget)**

**Method a1**: Estimating the value of insect pollination services to crops and wild goods cultivated or harvested at the focal site, using a desk-based method

This method is applicable when important insect-dependent crops and wild goods are cultivated or harvested only at the site of conservation interest and are not found around that site. The estimate of pollination services value should be calculated for up to five important insect-pollinated crops or wild goods found at the site under the current state. The total value of insect pollination services, *V*_Site_ (expressed in terms of $ yr^-1^) provided by the focal site to key crops and wild goods cultivated or harvested at that site can be estimated using Eq. (1):

$V_{S\mathrm{ite}} = \sum_{1}^{i} \left( {Y\max}_{i} \times\mathrm{DR}_{i} \times P_{i} \times A_{i} \right)$ (1)

where *Ymax_i_* is the maximum yield (tonnes ha^-1^ yr^-1^) for crop or wild good *i*, DR*_i_* is the dependency ratio of crop or wild good *i*, *P_i_* is the farmgate price of crop or wild good *i* ($ tonne^-1^) and *A_i_* is the total area (ha) of crop or wild good *i* harvested at the site. This method assumes that the insect pollination service is at its highest potential, as expressed in dependency ratios.

**Method a2**: Estimating the value of insect pollination services to crops and wild goods cultivated or harvested within an adjacent buffer of 1-km wide from the focal site, using a desk-based method

This method is applicable when important insect-dependent crops and wild goods are cultivated or harvested within the 1-km buffer around the focal site. The rate of pollinator visitation to the crop flowers decays with distance from the site, giving rise to an estimated decay curve (Ricketts et al. 2008). To estimate pollination service value for up to five types of key crop/wild goods cultivated or harvested within the buffer around the focal site under its current state, it is first necessary to establish what crops/wild goods are found within the buffer and their distances from the focal site. The buffer is divided into three distinct concentric zones with a width of approximately 300 m each, so that the innermost zone is adjacent to the focal site and the outermost zone is no more than 1 km from the perimeter of the site.

Visitation frequency parameter, vf(d) of insect pollinators to the crop at a distance, *d* from the focal site is then calculated for each zone using Eq. (2), which incorporates the decay rate in pollinator visitation from Ricketts et al. (2008), and assumes that visitation frequency is at a maximum within the focal site:

$\mathrm{vf}\left( d \right)=a \times e^{{} d}$ (2)

where *a* is the visitation frequency parameter at *d* = 0 m (i.e., at the perimeter of the focal site) where maximum visitation (*a* = 1) occurs, *e* is the inverse function of the natural logarithm (ln), and *μ_β_* is the decay rate = -0.00104, as specified in Table S3-1. The decay rate at -0.00118 or -0.00053 should be used instead if the site is within tropical, or temperate climate domain, respectively.

**Table S3-1:** Estimates of overall decay rate for pollinator visitation rate based on hierarchical Bayesian models (extracted from Ricketts et al., 2008). * denotes posterior probability that overall decay rate (*μ_β_*) is less than zero. ** denotes distance at which variable is 50% of the maximum value at distance = 0, along with 90% credible interval.

| Variable | Sample size | *μ_β_* | Pr (*μ_β_* <0)* | Point of 50% decay (m)** |
| --- | --- | --- | --- | --- |
| Visitation rate | 22 | -0.00104 | >0.996 | 668 (395 – 1,727) |
| Temperate | 11 | -0.00053 | >0.971 | 1,308 (437 – 13,849) |
| Tropical | 11 | -0.00118 | >0.959 | 589 (296 – 8,186) |

Visitation frequency parameter, vf(d) that represents each buffer zone obtained from Eq. (2) can then be used to estimate the total value of pollination services, *V*_Buffer_ (expressed in terms of $ yr^-1^) provided by the focal site to key crops and wild goods cultivated or harvested within the buffer around the site, by applying Eq. (3):

$V_{\mathrm{Buffer}} = \sum_{1}^{n} \left( \frac{\left( {Y\max}_{i}\times P_{i} {\times\mathrm{DR}}_{i} \right) \times\mathrm{vf}\left( d \right)}{a} - \frac{\left( {Y\max}_{i} \times P_{i} {\times\mathrm{DR}}_{i} \right) \times\mathrm{vf}\left( d=3000 \right)}{a} \right) \times A_{i, n}$ (3)

where *Y*max*_i_* is the maximum yield (tonnes ha^-1^ yr^-1^) for crop *i* in buffer zone *n*, vf(*d*) is the visitation frequency parameter at distance *d* from the focal site*, P_i_* is the farmgate price of crop *i* ($ tonne^-1^), *a* is the visitation frequency parameter at the focal site (*d* = 0 m) where visitation is at maximum level (*a* = 1), and *A_i,n_* is the total area (ha) of crop *i* within buffer zone *n*. This equation includes the deduction of the estimated pollination value at 3,000 m from the focal site, in order to exclude the baseline pollination services provided by insect pollinators that persist in the agricultural matrix (Fig. S3-1).

**
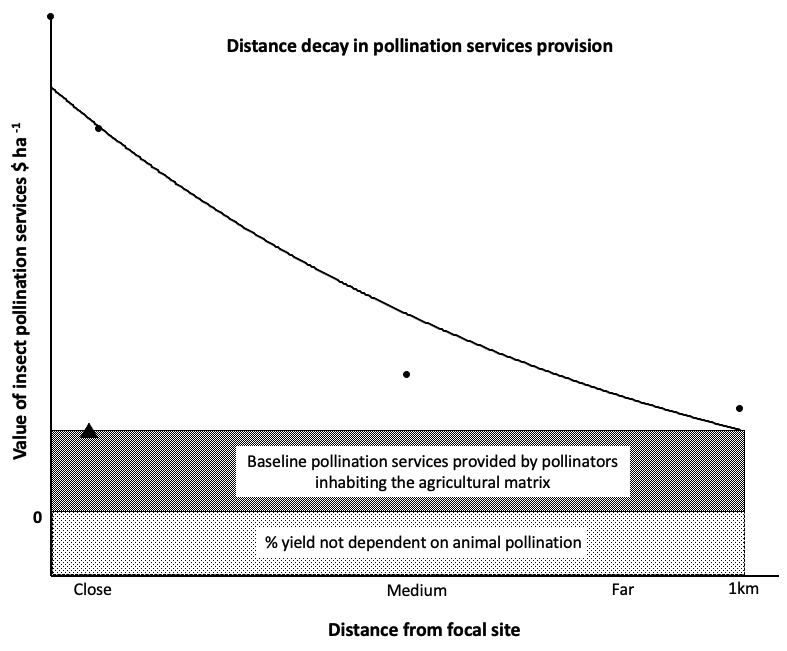
**

**Figure S3-1.** Graphical representation of a distance decay in pollination services provided by the focal site up to a distance of 1 km from the site. The black triangle represents the position of the alterative state of the focal site whereby the natural/semi-natural habitat is converted to insect-pollinated cropland.

Both methods 1a and 1b should be used when important insect-dependent crops and wild goods are cultivated or harvested at the focal site and also within the 1-km buffer around the site. The total pollination value from the focal site is the sum of the estimates derived from both methods.

**Method a3:** Estimating the value of insect pollination services provided by the focal site under the alternative state, using a desk-based method

If the total area of natural/semi-natural habitat at the focal site is to be converted into agricultural land that cultivates insect-dependent crops, the pollination value for the focal site under this alternative state, *V*_Alternative_ (expressed in terms of $ yr^-1^) for up to five important insect pollinator-dependent crops can be calculated using Eq. (4):

$V_{A\mathrm{lternative}} = \sum_{1}^{i} \left( {Y\max}_{i} \times\mathrm{DR}_{i} \times P_{i} \times A_{i} \right) \times e^{3000}$ (4)

where *Y*max*_i_* is the maximum yield (tonnes ha^-1^ yr^-1^) for crop *i*, DR*_i_* is the dependency ratio obtained from existing database for crop *i*, *P_i_* is the farmgate price of crop *i* ($ tonne^-1^), *A_i_* is the total area (ha) of crop *i* harvested at the site under the alternative state, μ_β_ is the overall decay rate for pollinator visitation (= -0.00104; see Table S3-1, but use value for tropics or temperate climate domain if appropriate).

Eq. (4) incorporates the decay rate in pollinator visitation to crop flowers (Fig. S3-1) and assumes that pollinator visitation rate in agricultural land (i.e., the alternative state) is equivalent to the decay rate in pollinator visitation to crop flowers observed at 3,000 m from the natural/semi-natural focal site. The decay at a distance of 3,000 m from the focal site is not the full spatial extent of decay in visitation rate away from natural/semi-natural habitat (see Ricketts et al., 2008). However, it exceeds the average foraging range for the majority of bee species (Greenleaf et al. 2007).

If the focal site is expected to be degraded, but will still retain its basic structure (e.g., logged forest), we assume that its pollination services will remain intact. Therefore, the value the site’s pollination services under this alternative state would be the same as that for focal site in its current state.

It is also possible to have a counterfactual whereby the focal site is likely to have only partial loss of its natural/semi-natural habitats. If the natural/semi-natural area at the altered focal site remain in greater proportion than its surrounding agricultural land, the pollination value of the site under the alternative state should be the fraction of the pollination value of the site in its current state, in relation to the proportion of the semi-natural/natural habitat retained after the land-use conversion. For example, we assume that a loss of 50% of the natural/semi-natural habitat at the focal site would proportionally reduce the value of pollination services provided by the site under the alternative state by 50%.

Lastly, we assume that there would be no pollination services provided under the alternative state whereby the total area of natural/semi-natural habitats at the focal site is converted into non-agricultural land (e.g., built-up area, water body, etc.) or into agricultural land that contains no insect-pollinator-dependent crops. Therefore, the value of pollination services for the focal site under that alternative state should be zero.

**b) Field observation surveys (medium budget)**

**Method b1**: Estimating the value of insect pollination services provided by the focal site, based on the observed visitation rate of insect pollinators as a proxy for pollination

This method uses the same existing data for the desk-based approach (i.e., the extent, maximum yield, DR and farmgate price of each of the key crop/wild goods [up to five types]), but also consider ecological information of insect pollinators by including field data obtained from direct observation surveys. It involves field data collection that requires the map of the site of conservation interest, stopwatch (or equivalent device for measuring exact timing), field data sheets, tape measure (for establishing observation sampling plots) and a tally counter (for counting insect pollinators). This method is applicable only when there are pollinator-dependent crops/wild goods cultivated or harvested at the focal site, **as well as** within the buffer of 1 km around the focal site. It provides the protocol for obtaining data on the frequency of insect-pollinator visitation (as a proxy for pollination services; Winfree et al. 2015) to the flowers of the crops/wild goods at the focal site for comparison with those within the three concentric buffer zones along a distance gradient radiating from the site, in order to estimate the decay rate for pollination services as a function of distance.

However, for situations where pollinator-dependent crops/wild goods are cultivated or harvested only at the focal site, but not within the buffer of 1 km around the site, the value of pollination services provided by the focal site, *V*_Site_ (expressed in terms of $ yr^-1^) should be estimated using the more straightforward desk-based approach, Eq. (5) instead. Hence, it is not necessary to collect field data on visitation frequency of insect pollinators at the focal site because we assume that the crops/wild goods cultivated or harvested at the site would have benefited from optimal level of pollination services (i.e., visitation frequency at the maximum level) and have the highest locally achievable yield.

$V_{S\mathrm{ite}} = \sum_{1}^{i} \left( {Y\max}_{i} \times\mathrm{DR}_{i} \times P_{i} \times A_{i} \right) \times\mathrm{vf}\left( d=0 \right)$ (5)

where vf(*d*=0) is the visitation frequency parameter at the focal site where d, distance from the focal site = 0 (set at the default maximum value = 1) and the rest of the parameters are the same as those in Eq. (1). Fundamentally, Eq. (5) provides the same estimate as Eqn. (1) from the desk-based method.

*Sampling at the focal site*

For each important pollinator-dependent crops/wild goods (up to five types), we recommend nine sampling locations to be randomly chosen at the focal site as evenly distributed across the site as possible, preferably at least 500 m apart to increase the chances of independence of sampled flower visitors. At each sampling location, three 1 m x 1 m plots are randomly placed (i.e. total of nine sampling locations and 27 plots for each crop/wild good type). The plot size should be adapted to the target crop/wild good. All insects that visited the flowers of the crop/wild good inside each plot are recorded for 15 minutes; only visits of insects that are large enough to contact the reproductive parts of the plants should be recorded. Visitation of multiple flowers by the same insect should be counted as a new visit for each flower. Ideally, the observation should be carried out by multiple recorders simultaneously or by one recorder on separate days at the same time of the day. The number of open flowers of the crop/wild good within each plot is counted to determine the observed visitation frequency, vf_obs_ expressed as number of visits per flower per minute (number of visits flower^-1^ min^-1^) (for further guidance on field observation technique, flower morphology, pollen vectors and insect pollinators, see Appendix S4). The observed visitation frequency, vf_obs_(*d* = 0) of insect pollinators at the focal site is the averaged number across the nine sampling locations in the focal site.

**Method b2**: Estimating the value of insect pollination services provided by the focal site, based on the observed visitation rate of insect pollinators as a proxy for pollination

*Sampling within the buffer around the focal site*

To determine the actual decay rate for visitation rate from the focal site, the area around that site is divided into three distinct concentric zones with a width of approximately 300 m each, so that the innermost zone is adjacent to the site and the outermost zone is no more than 1 km from the perimeter of the site. For each important pollinator-dependent crop/wild good, we recommend three sampling locations within each buffer zone to be randomly chosen. Where possible, these sampling locations should avoid close proximity to other natural/semi-natural habitats in the landscape to minimise their influence. At each sampling location, three 1 m x 1 m random plots are established (i.e., total of nine sampling locations and 27 plots across the distance gradient for each crop type). The mean observed visitation frequency, vf (d) for each buffer zone is obtained and converted to a monetary value ($ yr^-1^) using Eq. (6):

$V_{\mathrm{Buffer}} = \sum_{1}^{n} \left( \frac{\left( {Y\max}_{i}\times P_{i} {\times\mathrm{DR}}_{i} \right) \times\mathrm{vf}_{\mathrm{obs}}\left( d \right)}{\mathrm{vf}_{\mathrm{obs}}\left( d=0 \right)} - \frac{\left( {Y\max}_{i} \times P_{i} {\times\mathrm{DR}}_{i} \right) \times\mathrm{vf}_{\mathrm{obs}}\left( d>1000 \right)}{\mathrm{vf}_{\mathrm{obs}}\left( d=0 \right)} \right) \times A_{i,n}$ (6)

*where Y*max*_j_* is the maximum yield (tonnes ha^-1^ yr^-1^) for crop *i* in buffer zone *n*, vf_obs_(*d*) is the observed visitation frequency at distance, d from the focal site*, P_j_* is the farmgate price of crop *i* ($ tonne^-1^), and *A_i,n_* is the total area (ha) of crop, *i* within buffer zone *n*. This equation – similar to Eq. (3) – excludes the baseline pollination services provided by those pollinators that persist in the agricultural matrix. This baseline pollination is estimated by using observed visitation frequency, vf_obs_(*d* >1000) at the distance more than 1,000 m from the focal site (see Method b2).

Total value of pollination services provided by the focal site is the summation of *V*_Site_ and *V*_Buffer_, derived from Eq. (5) and Eq. (6), respectively.

**Method b2:** Estimating the value of insect pollination services provided by the focal site under the alternative state, based on the observed visitation rate of insect pollinators as a proxy for pollination

To estimate the value of insect pollination services provided by the focal site under alternative state whereby its total area of natural/semi-natural habitat is to be converted into agricultural land that cultivates insect-dependent crops, field observation data (on visitation frequency of insect pollinators) for each important pollinator-dependent crops/wild goods (up to five types) should be collected at the distance >1 km from the site, as a measure of background pollination services attributed to the agricultural matrix (that represent the alternative state). At this distance, we assume that the site does not provide significant additional pollination services beyond those delivered by the agricultural landscape. If possible, data should be collected at a distance of 3 km from the focal site, which exceeds the average foraging range for the majority of bee species (Greenleaf et al., 2007). The pollination value for the site under the alternative state, *V*_Alternative_ (expressed in $ yr^-1^) is calculated using Eq. (7) (for other type of alternative states, see guidance in Method a3):

$V_{\mathrm{Alternative}} = \sum_{1}^{i} \left( \frac{\left( {Y\max}_{i}\times P_{i} {\times\mathrm{DR}}_{i} \right) \times\mathrm{vf}_{\mathrm{obs}}\left( d>1,000 \right)}{\mathrm{vf}_{\mathrm{obs}}\left( d=0 \right)} \right)$ (7)

where *Y*max*_i_* is the maximum yield (tonnes ha^-1^ yr^-1^) for crop *i*, vf_obs_(*d*) is the observed visitation frequency at distance, *d* (expressed in m) from the focal site, *P_i_* is the farmgate price of crop, *i* ($ tonne^-1^), and *A_jn_* is the total area (ha) of crop *i* within buffer zone *n*.

**c) Empirical manipulation using exclusion experiment (high budget)**

**Method c1**: Estimating the value of pollination services provided by the focal site, using exclusion experiment

This method uses the pollinator exclusion techniques to directly derive the actual dependency ratios of the important insect-dependent crops/wild goods from the focal site and the buffer around the site. It involves setting up field exclusion experiments that requires the map of the site, 1-mm mesh bags (mosquito netting is ideal; size of mesh bags will be dependent on predicted size of the flower heads and of the pollinators), thread of two different colors, and garden tags. Guidance on setting up exclusion experiment is available at <https://bit.ly/2EGegwa>. To enhance the accuracy of exclusion experiments and to prevent the effect of bagging on seed/fruit development, bags were placed on the plants for as short a time as possible and removed at the end of the flowering period.

*Exclusion experiment at the focal site*

For each important pollinator-dependent crop/wild good at the focal site (up to five types), 15 plants at pre-flowering stage are randomly selected at the site for the exclusion experiment to estimate yield and pollination dependency ratio. On each plant, two floral units (flower or inflorescence) at similar pre-flowering stage are selected and randomly assigned to two treatments: (1) floral units are manipulated by being enclosed in mesh bags to prevent access by insect pollinators; and (2) unmanipulated floral units so that the flowers are accessible to wind and insect pollinators (control). If resources permit, 15 pairs of plants could be used instead, with individuals of each pair assigned to the two treatments (bagged and control).

At harvest, the yield of seeds or fruit is quantified for both treatment groups (i.e., bagged and control) for each plant. To estimate the proportion of yield due to wind and auto-pollination, the yield of bagged flowers is divided by the yield of unbagged flowers. The remaining proportion (i.e., 1 – proportion of yield due to wind and auto-pollination) can then be attributed to the yield due to insect pollination (dependency ratio, DR). The average dependency ratio (obtained from the 15 plants) is then used to estimate the value of pollination services (*V*_site_) provided by the focal site using Eq. (8).

$V_{S\mathrm{ite}} = \sum_{1}^{i} \left( {Y\max}_{i} \times\mathrm{DR}_{\mathrm{EE} i} \times P_{i} \times A_{i} \right)$ (8)

where DR_EE_ *_i_* is the dependence ratio obtained from the exclusion experiments for crop/wild good *i*, and the rest of the parameters are the same of those in Eq. (1).

*Exclusion experiment within the buffer around the site*

If there are important insect-dependent crops/wild goods in the area around the focal site, a buffer around the site should then be established with three distinct concentric zones with a width of approximately 300 m each, so that the innermost zone is adjacent to the site and the outermost zone is no more than 1 km from the perimeter of the site. Exclusion experiments as described above are then repeated within each zone, using five randomly-chosen pre-flowering plants of each important pollinator-dependent crop/wild good (up to five types). To improve the estimate of dependency ratio, five pairs of plants could be used instead within each zone, if resources permit, with individuals of each pair assigned to the two treatments (bagged and control). The average dependency ratio (obtained from all plants across the buffer) is then used to estimate the value of pollination services (*V_Buffer_*) to the crops/wild goods within the buffer around the focal site, using Eq. (9):

$V_{B\mathrm{uffer}} = \sum_{1}^{n} \left( {Y\max}_{i} \times\mathrm{DR}_{\mathrm{EE} i, n} \times P_{i} \times A_{i} \right)-\sum_{1}^{i} \left( {Y\max}_{i} \times\mathrm{DR}_{\mathrm{EE} i, d >1000} \times P_{i} \times A_{i} \right)$ (9)

where DR_EE_ *_I, n_* is the dependency ratio obtained from the exclusion experiments for crop/wild good *i* in zone *n*, DR_EE_ *_I,_*_d>1000_ is the dependency ratio obtained from the exclusion experiments for crop/wild good *i* at a distance *d* more than 1,000 m from the focal site, and the rest of the parameters are the same of those in Eq. (1). This equation – similar to Eq. (3) and Eq. (6) – excludes the baseline pollination services provided by those pollinators that persist in the agricultural matrix. This baseline pollination is estimated by using dependency ratio (DR_EE,_ *_i_*_, d>1000_) measured from the distance more than 1,000 m from the focal site (see Method c2).

Total value of pollination services provided by the focal site is therefore the summation of *V*_Site_ and *V*_Buffer_, both derived from Eq. (8) and Eq. (9), respectively.

**Method c2**: Estimating the value of pollination services provided by the focal site under the alternative state, using exclusion experiment

For the alternative state whereby the natural/semi-natural habitat at the focal site is being converted in agricultural land where the important insect-dependent crops/wild goods are cultivated or harvest, area outside the buffer (i.e., at the distance >1 km from the focal site) could be used as surrogate for the alternative state. Hence, exclusion experiments are carried out there to determine the dependency ratio of each crops/wild goods (up to five types), where we assume the focal site does not provide significant additional pollination services beyond those delivered by the agricultural matrix. Where possible, dependency ratio should be measured at least 3 km from the site, as such distance exceeds the average foraging range for the majority of bee species (Greenleaf et al., 2007). The actual dependency ratio is then used to estimate the pollination value of the site under the alternative state ($V_{Alternative}$), using Eq. (10).

$V_{A\mathrm{lternative}} = \sum_{1}^{i} \left( {Y\max}_{i} \times\mathrm{DR}_{\mathrm{EE} i, d>1000} \times P_{i} \times A_{i} \right)$ (10)

where DR_EE_*_, i,_* _d>1000_ is the dependency ratio from the distance, *d* more than 1,000 m from the focal site, and the rest of the parameters are similar to those in Eq. (1). For other type of alternative states, see guidance in Method a3.

**REFERENCES**

Greenleaf SS, Williams NM, Winfree R, Kremen C. 2007. Bee foraging ranges and their relationship to body size. Oecologia **153**:589–596.

Peh KS-H et al. 2013. TESSA: A toolkit for rapid assessment of ecosystem services at sites of biodiversity conservation importance. Ecosystem Services **5**:51–57.

Peh KS-H et al. 2017. Toolkit for Ecosystem Service Site-based Assessment (TESSA), 2nd edition. Cambridge, UK. Available from file:///Files/38/385b03a4-643c-42ee-8c6b-2f2f01aa16e1.pdf.

Ricketts TH et al. 2008. Landscape effects on crop pollination services: Are there general patterns? Ecology Letters **11**:499–515.

Winfree R, Fox JW, Williams NM, Reilly JR, Cariveau DP. 2015. Abundance of common species, not species richness, drives delivery of a real-world ecosystem service. Ecology Letters 18:626– 635.

- 1. Guidance on field data collection and dependency ratios

**This appendix includes:**

**1. Guidance on field data collection**

1.1. Flower reproductive parts

1.2. Pollen vectors

1.3. Flower visitors and pollinators

1.4. Sampling plots and observation of flower visitors

1.5. Sampling in highly heterogeneous landscapes

**2. Dependency ratios**

**1. Guidance on field data collection**

**1.1. Flower reproductive parts**

Flowers can be divided into three different types according to their reproductive parts (Fig. S4-1):

1. Flowers with **both** female and male reproductive parts (hermaphrodite)
2. Flowers with **male** reproductive parts **only**
3. Flowers with **female** parts **only**

It is important to identify unisexual flowers (about 10% of plant species) as you will only have to bag flowers with female reproductive parts (stigma, style and ovaries) in this case.

Some plants have both male and female flowers on the same individual plant (**monoecious**). Monoecious plants can be:

1. Monoecious hermaphrodite (with single-sex and hermaphrodite flowers)
2. Monoecious with pure male and pure female flowers

Some plants have male and female flowers on different plant individual (**dioecious**). See this [link](http://fruitandnuteducation.ucdavis.edu/generaltopics/AnatomyPollination/Flower_Anatomy/) for more information.

**
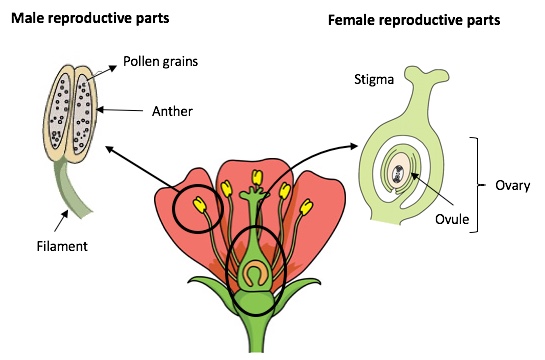
**

Fig. S4-1. Flower reproductive parts: male reproductive parts, left (stamen modified from <http://leavingbio.net/thestructureandfunctionsofflowers%5B1%5D.htm>); and female reproductive parts, right (carpel modified from <http://cronodon.com/BioTech/Plant_Bodies_Flowers.html>).

- 1. **Pollen vectors**

It is critical to know if the plant of interest is pollinated by animals. Table S4-1 provides general guidance on the identification of animal-pollinated flowers. However, it is only a rough guide and the flowers of an animal-pollinated plant may not exhibit all the associated characteristics.

Table S4-1. General characteristics of wind-pollinated flower and animal pollinated flowers.

|  | 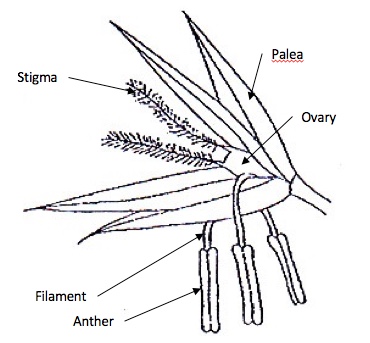 | *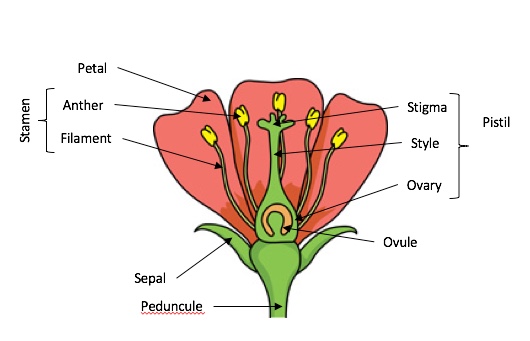* |
| --- | --- | --- |
|  | ***Wind-pollinated flowers*** | ***Animal-pollinated flowers*** |
| ***Flowers*** | Small flowers, often without petals | Large, colored or vivid white flowers. If small-sized, they might form a dense inflorescence |
| ***Scent*** | Lack scent | Scented |
| ***Nectar*** | Have no nectar | Can have nectar as a reward for pollinators |
| ***Anthers***  ***and pollen*** | Have exposed anthers with dry, powdery pollen that is released in clouds when the flowers are shaken | No release of pollen when you shake them; pollen in low quantity, often quite sticky |
| ***Stigma*** | Exposed, often feathery | Small and compact, not as exposed |
| ***Other features*** | Sometimes appear before leaves | Most importantly, insects and/or vertebrates visit the flowers and contact the sexual parts |

- 1. **Flower visitors and pollinators**

A pollinator can be defined as an animal that is a regular and non-destructive flower visitor, that transfers pollen between plants and that successfully pollinates the flower during visits, which culminates in the production of seeds (Carthew & Goldingay 1997). Therefore, a flower visitor is a pollinator if it makes contact with the reproductive parts of the plant, either picking up pollen grains or depositing pollen grains on the stigma (or both). In order to do the above, flower visitors need to be above a certain size. As effective pollination depends on many factors (pollen load, pollen deposited, etc.), it is hard to judge whether the visit was effective only through observations. Therefore, for the purpose of this protocol, any landing on the flower by an animal big enough to act as a pollinator is counted as a visit.

In addition to bees, other invertebrates and vertebrates visit flowers and may act as pollinators:

- Butterflies have four feathery wings, the colour of which varies with the species
- Beetles have a hard pair of wings that form a case (elyteron) enclosing the second pair of wings
- Birds also pollinate many species of plants

Some flowers, for instance cactus flowers, open at night (nocturnal anthesis); consequently, nocturnal animals such as bats can act as pollinators for these plants, especially in tropical regions. However, valuation of pollination services provided by vertebrate pollinators is beyond the scope of this project.

Table S4-2 provides guidance on how to distinguish different insect pollinators. It will be helpful to consult field guides to local pollinators before starting data collection to identify the main pollinators of the crops of interest. It may also be useful to identify (and be familiarised with) their common pests to avoid including them in the survey.

Table S4-2. General features of insect pollinators for field identification.

|  | **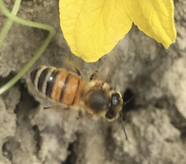** | **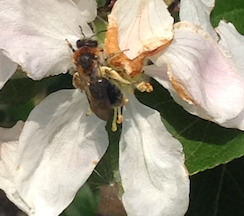** | **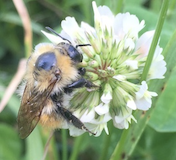** | **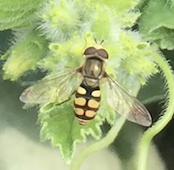** | **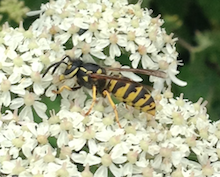** |
| --- | --- | --- | --- | --- | --- |
|  | **Honeybee** | **Solitary bee** | **Bumblebee** | **Hoverfly** | **Wasp** |
| **Wings** | Four wings | Four wings | Four wings | Two wings | Four wings |
|  | Folded into their body when at rest | Folded into their body when at rest | Folded into their body when at rest | Resting at an angle | Folded into their body when at rest |
| **Eyes** | Oval, more on the side of the head | Oval, more on the side of the head | Oval, more on the side of the head | Large, more to the front of the head | Oval, more on the side of the head |
| **Pollen** | Female carry pollen on pollen baskets (on back legs) | Female carry pollen on pollen baskets (on back legs) or under the body (Megachilidae) | Female carry pollen on pollen baskets (on back legs) | Carry pollen that sticks to their body, no pollen basket | Carry pollen that sticks to their body, no pollen basket |
| **Antennae** | Long | Long | Long | Short | Long |
| **Hair** | Smooth body, some hair on the thorax | Smooth body, some hair on the thorax | Furry all over the body | Usually hairless but flies which mimic bumblebees are furry | hairless |
| **Color** | Orange bands on abdomen, brown and black body | Varies across species. From yellow bands, orange to entirely black | Black, white, orange/yellow or red or large bands | Normally colors that mimic bees or wasps (black, yellow, orange) | Black or brown with yellow bands |

Photo credits. Honeybee, bumblebee, and hoverfly: Fabrizia Ratto; solitary bee, wasp: Emma Joslin.

- 1. **Sampling plots and observation of flower visitors**

The techniques for observing flower visitors are as follow:

- The number of floral units to observe should be set at the start of the observation period of 15 minutes. This number should be adjusted according to the density of the flowers so that observation is manageable. For example, the number of flowers within a sampling plot of 1 m^2^ can be counted if the flowers are small and abundant (such as oilseed rape); a wider observation area may be chosen for flowers that are less dense (such as melon). In any case, the number of flowers chosen for observation must be large enough to avoid null values whilst remaining manageable to observe.
- The size and the attractiveness of the floral units must be taken into account to avoid too many null values.
- Flowers that are in full exposure to the sun should be chosen. However, the observer should stand in a position that does not cast a shadow on the plant/flowers. The observer should also stand far enough from the flowers to avoid changing the behaviour of pollinators.
- All visit to flowers in the observation plot must be recorded. Every time a pollinator lands on a new flower, it should be counted as a new visit, even if it is an individual that has already recorded on another flower.
- Pollinators can be recorded in one single group, or classified according to their morphospecies (i.e. honeybees, bumblebees, other bees, and other pollinators) depending on the level of the observer’s identification skills.
- Observations should only be carried out during dry, sunny weather with ambient temperatures ≥ 13°C and only between 10:00 h and 16:00 h.
- If the ambient temperature is between 13 and 17°C, observations may be carried out when cloud cover is less than 40%. Observations may be carried out in any dry weather when the ambient temperature is over 17°C (Pollard & Yates, 1993).
- Wind speed should be below 5 on the Beaufort wind force scale (Table S4-3; Pollard, 1977).

Table S4-3. Beaufort wind scale.

| **Beaufort force** | **Description** | **When you see or feel this effect** | **Wind**  **(km/h)** |
| --- | --- | --- | --- |
| 0 | Calm | Smoke goes straight up | <2 |
| 1 | Light air | Wind direction is shown by smoke drift but not by wind vane | 2–5 |
| 2 | Light breeze | Wind is felt on the face; leaves rustle; wind vanes move | 6–11 |
| 3 | Gentle breeze | Leaves and small twigs move steadily; wind extends small flags straight out | 12–19 |
| 4 | Moderate breeze | Wind raises dust and loose paper; small branches move | 20–29 |
| 5 | Fresh breeze | Small trees sway; waves form on lakes | 30–39 |
| 6 | Strong breeze | Large branches move; wires whistle; umbrellas are difficult to use | 40–50 |
| 7 | Moderate gale | Whole trees are in motion; walking against the wind is difficult | 51–61 |
| 8 | Fresh gale | Twigs break from trees; walking against the wind is difficult | 62–74 |
| 9 | Strong gale | Buildings suffer minimal damage; roof shingles are removed | 75–87 |
| 10 | Whole gale | Trees are uprooted | 88–101 |
| 11 | Violent storm | Widespread damage | 102–116 |
| 12 | Hurricane | Widespread destruction | 117+ |

- 1. **Sampling in highly heterogeneous landscapes**

Our methods are developed to detect the spill-over of pollination services from a natural/semi-natural site into the adjacent cropland, and they do not account for overspill of pollination services from other nearby patches of natural/semi-natural habitat. In highly heterogeneous landscapes, it may not be possible to establish transects far enough from other natural patches. Therefore, our methods could result in an overestimation of the value of pollination services provided by the focal site. The pollination protocol, however, partly controls for the pollination attributed to the agricultural matrix and other natural/semi-natural habitats in the landscape by deducting the pollination service value at 1 km (baseline value) from the focal site. In highly heterogeneous landscapes, defined as those containing >20% natural/semi-natural habitat (Tscharntke et al. 2005), an increase in the number of transects radiating from the focal site to cover the entire buffer area could improve the estimation of the pollination value of that site.

**REFERENCES**

Carthew SM, Goldingay RL. 1997. Non-flying mammals as pollinators. Trends Ecol. Evol. **12**:104–108.

Pollard E. 1977. A method for assessing changes in the abundance of butterflies. Biological Conservation **12**:115–134. Available from http://linkinghub.elsevier.com/retrieve/pii/0006320777900659 (accessed May 22, 2017).

Pollard E, Yates T. 1993. Monitoring Butterflies for Ecology and Conservation. Springer Netherlands.

Tscharntke T, Klein AM, Kruess A, Steffan-Dewenter I, Thies C. 2005. Landscape perspectives on agricultural intensification and biodiversity - Ecosystem service management. Ecology Letters **8**:857–874.

**2. Dependency ratio**

Table S4-5. Dependency ratios (DR) of the common crops (compiled from Klein et al. 2007; the list is not exhaustive). Impact by animal pollination on each crop is classified using Klein's Levels of dependency (Klein et al. 2007): **essential**, pollinators essential for most varieties (production reduction by 90% more, comparing experiments with and without animal pollinators); **high**: animal pollinators are extreme (40 to less than 90% reduction); **modest**: animal pollinators are clearly beneficial (10 to less than 40% reduction); **little**: some evidence suggests that animal pollinators are beneficial (greater than 0 to less than 10% reduction); and **no increase**: no production increase with animal-mediated pollination.

| **Crop species** | **Vernacular name** | | **Impact by animal pollination** | | **(DR)** | | **Pollinators and visitors** | | **Pollination without visitors** | | **Breeding** | | **Reference** |
| --- | --- | --- | --- | --- | --- | --- | --- | --- | --- | --- | --- | --- | --- |
| **Vegetable crops** | | | | | | | | | | | | | |
| *Abelmoschus esculentus* | Okra, Gumbo | | **modest** | | **0.25** | | honey bees (*Apiscerana*), solitary bees (*Halictus spp.)* | | passive self-pollination | | hermaphrodite, self-compatible | | Crane, 1991; Hamon, 1991; Free, 1993 |
| *Cajanus cajan* | Pigeon pea, Cajan pea, Congo bean | | **little** | | **0.05** | | honey bees, solitary bees (*Megachile sp., Xylocopa sp., Chalicodoma sp*.) | | passive self-pollination | | hermaphrodite, self-compatible | | James et al., 1989; Grewal et al., 1990; Free, 1993; Heard, 1999 |
| *Canavalia ensiformis, C. gladiata, C. marittima, C. microcarpa, C. Virosa* |  | **modest** | | **0.25** | | solitary bees (*Xylocopa confusa*) | | passive self-pollination, wind pollination | | hermaphrodite, self-compatible | | Free, 1993; Gross, 1993 for *C. rosea* | |
| *Capsicum annuum, C. frutescens* | Chile pepper, Red pepper, Bell pepper, Green pepper | | **little** | | **0.05** | | honey bees, stinglee bees (*Melipona favosa, M. subnitida*), bumble bees (*Bombus impatiens, B. terrestris*), solitary bees (*Osmia cornifrons, Megachile rotundata*), hover flies (*Eristalis tenax*) | | wind- or insect- mediated shaking necessary for self- pollination, passive self-pollination | | hermaphrodite, self-compatible | | Jarlan et al., 1997a, b; Meisels & Chiasson, 1997; Raw, 2000; Dag & Kammer, 2001; Ercan & Onus, 2003; De Oliveira Cruz et al., 2005; Slaa et al., 2006 (pollinators were deemed important in greenhouses for enhancing fruit weight, but less in open fields) |
| *Chenopodium quinoa* | Quinoa | | **no increase** | | **0** | | flies | | passive self- pollination, wind pollination | | hermaphrodite, andro- monoecious | | Simmonds, 1965; Simmonds, 1971 |
| *Cicer arietinum* | Chickpea, Gram, Garbanzo bean | | **no increase** | | **0** | | honey bees, solitary bees | | passive self-pollination | | hermaphrodite, self-compatible | | Free, 1993; Abbo et al., 2003 |
| *Citrullus lanatus* | watermelon | | **essential** | | **0.95** | | honey bees (*Apis cerana*), bumble bees (*Bombus californicus, B. impatiens, B. vosnesenski*i) solitary bees (*Halictus tripartitus, Peponapis pruinosa*), species effective in pollen deposition are listed in Kremen et al. 2002 | |  | | mostly monoecious, self-compatible | | Free, 1993; Stanghellini et al., 1997; Stanghellini et al., 1998; Delaplane & Mayer, 2000; Kremen et al., 2002; Stanghellini et al., 2002; Kremen et al., 2004; Njoroge et al., 2004 |
| *Cucumis melo* | Cantaloupe, Melon | | **essential** | | **0.95** | | honey bees (*Apis mellifera*), bumble bees *Bombus sp.,* solitary bees (*Ceratina sp*.) | | passive self- pollination possible only in andro- monoecious varieties | | monoecious or andro- monoecious, self-compatible | | Free, 1993; Norden, 1985; Kato & Nogueira-Couto, 2002; Valantin-Morison et al., 2006 |
| *Cucumis sativus* | Cucumber, Gherkin | | **great** | | **0.65** | | honey bees (*Apis mellifera*), bumble bees (*Bombus impatiens*), solitary bees (*Melissodes sp*.) | | passive self- pollination possible only in andro- monoecious varieties | | monoecious or andro- monoecious, self-compatible | | Free, 1993; Stanghellini et al., 1997; Gingras et al., 1999; Stanghellini et al. 2002; Slaa et al., 2006 (parthenocarpic, gynoecious varieties of slicing cucumber grown in greenhouses to prevent insect pollination which could be detrimental to fruit quality) |
| *Cucurbita maxima, C. mixta, C. moschata, C. pepo* | Pumpkin, Squash, Gourd, Marrow, Zucchini | | **essential** | | **0.95** | | honey bees (*Apis cerana, A. mellifera*), stingless bees (*Scaptotrigona depilis*), solitary bees, (*Pithitis smaragdula, Peponapis limitaris, P. pruinosa, Xenoglossa sp., Ceratina sp.*) | |  | | monoecious, self-compatible | | Norden, 1985; Free, 1993; Nepi & Paccini, 1993; Delaplane & Mayer, 2000; Canto-Aguilar & Parra-Tabla, 2000; Ashworth & Galetto, 2001; Cardoso ,2003 (higher germination rate and vigour with natural pollination compared to hand pollination); Fuchs & Müller, 2004 |
| *Cyamopsis tetragonoloba* | Guar bean, Goa bean | | **little** | | **0.05** | | honey bee | | passive self- pollination | | hermaphrodite | | Free, 1993 |
| *Dolichos biflorus, D. lablab* | Hyacinth bean, Horse- gram, Lablab | | **modest** | | **0.25** | | honey bee | | passive self- pollination | | hermaphrodite | | Garcia Neto et al., 1988; Free, 1993 |
| *Fagopyrum esculentum* | Buckwheat | | **great** | | **0.65** | | honey bee | |  | | hermaphrodite, self- incompatible (distylous) | | Free, 1993; Björkman, 1995a,b; Campbell, 1997; Goodman et al., 2001 |
| *Lens esculenta* | Lentils | | **no increase** | | **0** | | bees | | passive self- pollination | | hermaphrodite, self-compatible | | Ladizinsky et al., 1984; Erskine & Muehlbauer, 1991; Free, 1993; Richards, 2001 |
| *Lycopersicon esculentum* | Tomato | | **little** | | **0.05** | | honey bees (*Apis mellifera*), stingless bees (*Melipona quadrifasciata, Nannotrigona perliampoides*), bumble bees (*Bombus hypnorum*, B.*Thoraco- bombus* *pascuorum, B. sonorus, B. terrestris, B. vosnesenskii),* solitary bees (*Amegilla chlorocyanea, A. (Zonamegilla) holmesi, Xylocopa ssp*.) | | wind- or insect- mediated shaking necessary for self-pollination, parthenocarpy | | hermaphrodite, self-compatible, buzz-pollination | | Free, 1993; du Toit, 1994; Asada & Ono, 1996; Delaplane & Mayer, 2000; Hogendoorn et al., 2000; Westerkamp & Gottsberger, 2000; Morandin et al., 2001; Cauich et al., 2004; Higo et al., 2004; Greenleaf, 2005; Bell et al., 2006; Hagendoorn et al., 2006; Greenleaf & Kremen, 2006a; Slaa et al., 2006 |
| *Mucuna pruriens (*syn*. Stizolobium* spp*.)* | Velvet bean | | **little** | | **0.05** | | honey bees (*Apis dorsata, Apis florea*), bumble bees, thrips | | passive self- pollination | | hermaphrodite, self-compatible | | Du Toit, 1990; Crane, 1991; Roubik, 1995; Carrek & Williams, 1998; Ibarra-Perez et al., 1999 |
| *Phaseolus spp. (P. vulgaris, P. lunatus, P. angularis, P. aureus, P. mungo, P. coccineus, P. calcaratus, P. aconitifolius, P. acutifolius* | Kidney bean, Haricot bean, Lima bean, Adzuki bean, Mungo bean, String bean | | **little** | | **0.05** | | honey bees (*Apis dorsata, Apis florea*), bumble bees, thrips | | passive self- pollination | | hermaphrodite, self-compatible | | Du Toit, 1990; Crane, 1991; Roubik, 1995; Carrek & Williams, 1998; Ibarra-Perez et al., 1999 |
| *Pisum sativum, P. arvense* | Garden pea, Field pea | | **no increase** | | **0** | | bumble bees, solitary bees (*Eucera dalmatica, Xylocopa ssp.)* | | passive self- pollination | | hermaphrodite, self-compatible | | Gritton, 1980; Free, 1993, Franklin et al., 2000; Mcphee, 2003 |
| *Psophocarpus tetragonolobus* | Winged bean, Goa bean | | **unknown** | |  | | solitary bees (*Xylocopa confusa*) | | passive self- pollination | | hermaphrodite, dichogamous, self- incompatible | | Free, 1993 |
| *Solanum melongena* | Eggplant, Aubergine | | **modest** | | **0.25** | | wind- or insect- mediated shaking necessary for self- pollination, passive self- pollination | | buzz-pollination | | hermaphrodite, self-compatible, | | Free, 1993 |
| *Vigna unguiculata* | Cowpea, Blackeye pea, Blackeye bean | | **little** | | **0.05** | | honey bees, bumble bees | | passive self- pollination | | hermaphrodite, self-compatible | | Vaz et al., 1998 |
| *Vigna subterranea (*syn*.Voandzeia subterranea)* | Bambara beans, Bambara groundnuts, Earth pea | | **little(indirect)** | |  | | ants (indirect effect on fruit set), bees mentioned in Roubik (1995, no source given) | | passive self- pollination | | hermaphrodite, self-compatible | | Free, 1993, |
| **Fruit crops** | | | | | | | | | | | | | |
| *Actinidia deliciosa* | Kiwifruit | | **essential** | | **0.95** | | honey bees (*Apis mellifera*),bumble bees (*Bombus terrestris*), solitary bees | | wind pollinated but few, small fruits of low quality | | dioecious | | Costa et al., 1993; Free, 1993; Vaissière et al., 1996; Awasthi & Kumar, 1997; Gonzalez et al., 1998; Delaplane & Mayer, 2000; Howpage et al., 2001 |
| *Annona squamosa* | Atemoya,Cherimoya,Custard apple | | **essential** | | **0.95** | | nitidulid beetles (*Carpophilus hemipterus, Carpophilus mutilatus*) | | passive self-pollination, hand pollination | | hermaphrodite | | Galon et al., 1982; Gazit et al., 1982; George et al., 1989; George et al., 1992; Free, 1993; Nadel & Pen,a 1994; Peña et al., 1999; Kill & da Costa, 2003; Blanche & Cunningham, 2005 |
| *Arbutus unedo* | Tree-strawberry | | **modest(indirect)** | | **0.25** | | honey bees (*Apis mellifera*), bumble bees (*Bombus terrestris*) | | wind-mediated shaking can lead to self-pollination | | hermaphrodite, self-compatible | | Sealy, 1949; Hagerup, 1957; Herrera et al., 1984; Rasmont et al., 2005 |
| *Artocarpus altilis* (syns. *A. incisus, A.incircus, A.incisa, A.communis)* | Breadfruit | | **unknown** | |  | | stingless bees | | parthenocarpy in seedless varieties, wind pollination | | monoecious, dichogamous | | Morton, 1987; Hasan & Razak, 1992; Free, 1993; Ragone, 1997; Heard, 1999 |
| *Artocarpus heterophyllus* (syns. *A. integrifolius, A. integrifolia)* | jackfruit | | **unknown** | |  | | stingless bees, flies and moths as flower visitors | | parthenocarpy, wind pollination | | monoecious, dichogamous | | Moncur, 1985; Morton, 1987; Heard, 1999; Sakai & Kato, 2000; Devy & Davidar 2003; insect-assisted wind pollination hypothesized by Brantjes (1981) |
| *Asimina triloba* | Pawpaw, Indiana banana | | **essential** | | **0.95** | | carrion flies and dung flies | |  | | hermaphrodite, self-incompatible | | Willson & Schemske ,1980; Gottsberger, 1999; Pomper et al., 2003 |
| *Averrhoa carambola* | Carambola, Starfruit | | **great** | | **0.65** | | honey bees (*Apis cerana*), stingless bees (*Trigona thoracica*) | | passive self-pollination | | hermaphrodite, distylous, self-incompatible | | Free, 1993; Heard, 1999; Richards, 2001 |
| *Carica papaya* | Papaya | | **little** | | **0.05** | | honey bees, thrips, large sphinx, hummingbirds, moths, butterflies | | passive self-pollination, wind pollination, parthenocarpy | | dioecious, monoecious, hermaphrodite, self-compatible | | Free, 1993; Jindal & Sharma, 1997; Westerkamp & Gottsberger, 2000 |
| *Citrus aurantifolia, C. aurantium, C. bergamia, C. grandis, C. limetta, C. limon, C. maxima, C. medica (var. cedrata), C. myrtifolia, C. paradisi, C. reticulata, C. sinensis, C. unshiu, Fortunella japonica* | Bergamot,Chinot, Citron, Clementine, Grapefruit, Kumquat, Lemmon, Lime, Manderine, Orange, Pomelo, Tangerine | | **little** | | **0.05** | | honey bees (*Apis cerana, A.mellifera*), bumble bees (*Bombus sp*.) | | variable passive self-pollination and parthenocarpy differs greatly among species and varieties | | hermaphrodite (most species); variable level of self-compatibility depending on species and varieties | | Crane. 1991; Free. 1993; Bhatia et al., 1995; Sharma & Jindal. 1997; Wallace & Lee. 1999; Sanford. 2003; Chacoff. 2007; Chacoff & Aizen. 2006 |
| *Chrysophyllum cainito (*syn*. Achras caimito)* | Star apple, Cainito | | **little(indirect)** | | **0.05** | | bats, insects | |  | | hermaphrodite | | Morton. 1987; Degen et al., 2001 |
| *Crataegus azarolus* (syn.*C. ruscionensis*) | Azarole, Azzeruolo | | **little(indirect)** | | **0.05** | | honey bees, midges | | apomixis, but initiation requires pollination | | hermaphrodite, self-compatible | | Phipps, 2003; Dönmez, 2004 |
| *Dimocarpus longan (*syn*. Euphoria longan, Euphoria longana,Nephelium longana)* | Longan, Lungan | | **little** | | **0.05** | | honey bees (*Apis mellifera*), stingless bees (*Trigona spp.*) | | passive self-pollination, wind pollination | | polygamous | | Heard, 1999; Liu & Ma, 2001; Blanche et al., 2006b |
| *Diospyros kaki; D. virginiana* | Persimmon | | **little** | | **0.05** | | honey bees (*Apis cerana, A. mellife*ra), bumble bees, solitary bees | | variable level of parthenocarpy among varieties | | monoecious, dioecious, rarely polygamous | | Miura, 1982; Crane, 1991; Mehta & Kashyap, 1997 |
| *Durio zibethinus* | Durian | | **great** | | **0.65** | | honey bees (*Apis dorsata*), bats (*Eoncyteris spelaea*), birds | |  | | hermaphrodite, monoecious, mainly self-incompatible | | Morton, 1987; Salakpetch et al., 1992; George et al., 1994; Yaacob & Subhadrabandhu, 1995; Husin & Abidin, 1998; Lim & Luders, 1998; Weterkamp & Gottsberger, 2000 |
| *Eriobotrya japonica (*syn*. Mespilus japonicus)* | Loquat, Japanese plum, Japanese medlar | | **great** | | **0.65** | | honey bees (*Apis cerana*), bumble bees | | passive self- pollination | | hermaphrodite, self-incompatible | | Khan et al., 1986, Morton, 1987; Crane, 1991; Free, 1993; Sharma & Jinda, l1997 |
| *Feijoa sellowiana* | Feijoa | | **great** | | **0.65** | | birds (*Turdus merula, Acridotheres tristis*), honey bees, honey bees | | passive self- pollination, wind pollination | | hermaphrodite, varying level of self- incompatibility among varieties | | Schroeder, 1953; Stewart, 1984, 1989; Patterson, 1990; Free, 1993 (DR varies among varieties); Ducroquet & Hickel, 1997; Degenhard et al., 2001 |
| *Ficus carica* | Ficus | | **modest** | | **0.25** | | wasp (*Blastophaga psenes*) | | variable level of parthenocarpy among varieties | | gyno-dioecious, monoecious | | Free, 1993; Westerkamp & Gottsberger, 2000 |
| *Fragaria ssp* | Strawberry | | **modest** | | **0.25** | | honey bees (*Apis mellifera*), stingless bees (*Trigona angusula, T.* (Tetragonula) minangkabau, Nannotrigona testaceicornis), bumble bees, solitary bees (*Osmia cornuta*), hover flies | | passive self- pollination, little wind pollination | | hermaphrodite (most varieties), self-compatible | | Maeta et al., 1992; Chagnon et al., 1993; Free, 1993; Kakutani et al., 1993; Zebrowska, 1998; Delaplane & Mayer, 2000; Malagodi-Braga & Kleinert, 2004 |
| *Litchi chinensis* |  | | **little** | | **0.05** | | honey bees (*Apis sp.),* flies | | little self-pollination, wind pollination | | andro- monoecious, self-compatible | | Free, 1993; Bhatia et al., 1995, Stem & Gazit 1996; in Sharma & Jindal 1997 |
| *Malus domestica* | Apple | | **great** | | **0.65** | | honey bees (*Apis cerana, A. mellifera*), bumble bees (*Bombus sp., solitary bees (Andrena sp., Anthophora*) sp., (*Osmia cornifrons, O. lignaria propinqua, O. rufa*), hover flies (*Eristalis cerealis, E. tenax*) | | passive self- pollination, parthenocarpy in some varieties | | hermaphrodite, mainly self- incompatible | | Crane, 1991; Free, 1993; Sekita & Amada, 1993; Fourez, 1995; Batra, 1998; Delaplane & Mayer, 2000; Westerkamp & Gottsberger, 2000; Vicens & Bosch, 2000; Kron et al., 2001; Sekita, 2001; Stern et al., 2001; Thomson & Goodell, 2001; Wei et al., 2002; Soltész, 2003; Ladurner et al., 2004; Sharma et al., 2004, Garratt et al., 2013 |
| *Mammea americana (*syn. *Mamea americana)* | Mamee | | **modest(indirect)** | | **0.25** | | bees | |  | | andro-dioecious passive self- pollination | | Morton, 1987; Roubik, 1995; Dunthorn, 2004 |
| *Mangifera indica* | Mango | | **great** | | **0.65** | | honey bees (*Apis sp.),* stingless bees (*Trigona sp*.), flies, ants, wasps | | passive self- pollination, wind pollination | | andro- monoecious, variable self- compatibility among varieties | | Free, 1993; du Toit, 1994; Bhatia et al., 1995; Dag et al., 2001 |
| *Manikara zapotilla (*syn. *Manikara zapota, Achras sapota)* | Sapodilla | | **essential** | | **0.95** | |  | |  | | hermaphrodite, largely self- incompatible Thrips (*Thrips hawaiiensis, Haplothrips tenuipennis* | | Piatos & Knight, 1975; Reddi, 1989; Mickelbart, 1996 |
| *Mespilus germanica* | Medlar | | **unknown** | |  | | honey bees (*Apis mellifera*) | | passive self- pollination, parthenocarpy | | hermaphrodite, self-compatible | | Reiter, 1947; Phipps, 2003 |
| *Nephelium lappaceum* | Rambutan | | **little** | | **0.05** | | honey bees (*Apis cerana*), stingless bees, flies | | apomixis in some varieties | | hermaphrodite (functional gynoecious), androecious | | Roubik, 1995; Heard, 1999; Slaa et al., 2006 |
| *Opuntia ficus- indica* | Prickly pear | | **modest(indirect)** | | **0.25** | | bumble bees | | parthenocarpy | | hermaphrodite, mostly self- incompatible | | Grant & Hurt, 1979; Weiss et al., 1993; DeFelice, 2004 |
| *Passiflora edulis* | Passion fruit, Maracuja | | **essential** | | **0.95** | | carpenter bees (=solitary bees) (*Xylocopa frontalis, X. suspecta*) bumble bees, hummingbirds | | passive self- pollination in some varieties (Bruckner et al.1995), hand pollination | | hermaphrodite, most varieties largely self- incompatible | | Corbert & Willmer, 1980; Free, 1993; Brancher, 1999; Camillo, 1996; Da Silva, 1999; Delaplane & Mayer, 2000; Westerkamp & Gottsberger, 2000; Almeida Lima, 2002; Freitas & De Oliveira Filho, 2003 |
| *Persea americana* | Avocado | | **great** | | **0.65** | | honey bees, stingless bees, solitary bees | | passive self- pollination | | hermaphrodite, dichogamous, self- incompatible | | Vithanage, 1990; Free, 1993; Ish-Am & Eisikowitch, 1993, du Toit, 1994; Ish-Am, 1998a, b; Ish-Am, 1999 Heard, 1999; Delaplane & Mayer, 2000; Gazit & Degani, 2002; Can- Alonso et al., 2005 |
| *Pouteria sapota* (syns. *Calocarpum sapota*, *Calocarpum mammosum, Pouteria mammosa*) | Sapote, Mamey colorado | | **unknown** | |  | | honeybees | | unknown | | hermaphrodite, night anthesis | | Morton, 1987; Davenport & O’Neal, 2000 |
| *Prunus domestica, P. spinosa* | Plum, Greengage, Mirabelle, Sloe | | **great** | | **0.65** | | honey bees (*Apis mellifera*), bumble bees, solitary bees (*Osmia lignaria propinqua*), flies | | passive self- pollination | | hermaphrodite varieties self- incompatible or self-compatible | | Free, 1993; Calzoni & Speranza, 1998; Delaplane & Mayer, 2000; Westerkamp & Gottsberger, 2000; Frève et al., 2001; Szábo, 2003 |
| *Prunus persica, Persica laevis* | Peach, Nectarine | | **great** | | **0.65** | | honey bees (*Apis mellifera*), bumble bees, solitary bees (*Osmia cornifrons, O. lignaria propinqua*), flies | | passive self- pollination | | hermaphrodite, self-compatible | | Free, 1993; Delaplane & Mayer, 2000; Westerkamp & Gottsberger, 2000; da Mota & Nogueira-Couto, 2002; Szábo et al., 2003b |
| *Prunus avium* | Sweet cherry | | **great** | | **0.65** | | honey bees (*Apis mellifera*), bumble bees, solitary bees (*Osmia lignaria*), flies | | passive self- pollination | | hermaphrodite, mostly self- incompatible | | Bosch & Kemp, 1999; Delaplane & Mayer, 2000; Nyéki et al., 2003a; Bosch et al., 2006 |
| *Prunus armeniaca* | Apricot | | **great** | | **0.65** | | honey bees (*Apis mellifera*), bumble bees,solitary bees (*Osmia cornifrons, O. lignaria propinqua*), flies | | passive self- pollination | | hermaphrodite (old-world varieties self- compatible, new-world varieties self- incompatible) | | Free, 1993; McLaren et al., 1995; Austin et al., 1996; Delaplane & Mayer, 2000; Westerkamp & Gottsberger, 2000; Szábo et al., 2003a; Benedek et al., 2006; Vaissière et al., 2006 |
| *Prunus cerasus* | Sour cherry | | **great** | | **0.65** | | honey bees (*Apis mellifera*), bumble bees, solitary bees, flies | | passive self- pollination | | hermaphrodite, varying level of self- compatibility | | Free, 1993; Delaplane & Mayer, 2000; Nyéki et al., 2003b |
| *Psidium guajava* | Guava, Guayaba | | **modest** | | **0.25** | | honey bees (*Apis mellifera*), stingless bees (*Trigona cupira*), bumble bees (*Bombus mexicanus*), solitary bees (*Lasioglossum spp.)* | | passive self- pollination | | hermaphrodite, self-compatible | | Hedström, 1988; Sharma & Jindal, 1997; Lakshmi & Mohana Rao, 1998; Heard, 1999 |
| *Punica granatum* | Pomegranate | | **modest** | | **0.25** | | honey bees, beetles (*Cetonia, Trichodes*) | | passive self- pollination | | hermaphrodite, andro- monoecious, partly self- incompatible | | Free, 1993; Knuth, 1908; Rana & Dwivedi, 1997; Melgarejo et al., 2000; Derin & Eti, 2001; Mars & Marrakchi, 2004 |
| *Pyrus communis* | Pear | | **great** | | **0.65** | | honey bees (*Apis mellifera*), bumble bees, solitary bees (*Osmia sp*.), flies (*Eristalis sp.*) | | passive self- pollination | | hermaphrodite, lself- incompatible | | Free, 1993; Delaplane & Mayer, 2000; Westerkamp & Gottsberger, 2000; Maccagnani et al., 2003; Nyéki & Soltész, 2003; Monzón et al., 2004; Stern et al., 2004 |
| *Ribes nigrum, R. rubrum* | Black currant, Red currant | | **modest** | | **0.25** | | honey bees (Apis mellifera), bumble bees (*Bombus sp*.), solitary bees | | passive self- pollination | | hermaphrodite, varying degree of self- incompatibility depending on species and variety | | Free, 1993; Koltowski et al., 1997; Koltowski et al., 1999; Soltész et al., 2003b |
| *Rosa* spp. (*R. canina* and all other spp. in section Caninae) | Rose hips, Dogroses | | **great** | | **0.65** | | honey bees, bumble bees (*Bombus spp.),* carpenter bees (*Xylocopa spp*.), solitary bees, hover flies (*Eristalis spp*.) | | self-pollination, parthenocarpy | | hermaphrodite, varying degree of self- compatibility depending on species and hybrids | | Jicinska, 1976; Stougaard, 1983; Yeboah Gyan & Woodell, 1987; Kevan et al., 1990; Ueda & Akimoto, 2001; Kevan, 2003 |
| *Rubus idaeus,R. fruiticosus,R.chamaemorus,R. flagellaris, R. trivalis* | Raspberry, Blackberry, Cloudberry, Northern Dewberry | | **great** | | **0.65** | | honey bees (*Apis mellifera*), bumble bees (*Bombus spp*.),solitary bees (*Osmia aglaia, O.cornuta*),hover flies (*Eristalis spp*.) | | passive self- pollination yielding inferior fruits | | hermaphrodite, self-compatible | | Yeboah Gyan & Woodell, 1987; Chagnon et al., 1991; Free, 1993; Willmer et al., 1994; Pinzauti et al., 1997; Pelletier et al., 2001; Cane, 2005 |
| *Sambucus nigra* | Elderberry | | **modest** | | **0.25** | | honey bees, flies, longhorn beetles | | passive self- pollination | | hermaphrodite, self-compatible | | Bolli, 1994 |
| *Solanum quitoense* | Naranjillo | | **great** | | **0.65** | | bumble bees | |  | | hermaphrodite, self-compatible, buzz-pollination | | Heiser et al., 1972; Roubik, 1995; Almanza et al., 2006 |
| *Sorbus aucuparia* | Rowanberry | | **essential** | | **0.95** | | honey bees, bumble bees, syrphid flies | | passive self-pollination | | hermaphrodite, self-incompatible | | Campbell et al., 1991; Bixby & Levin, 1996; Sperens, 1996; Raspé, 1998; Pías & Guitián, 2006 |
| *Sorbus domestica* | Service-apple | | **modest(indirect)** | | **0.25** | | bees, flies | |  | | hermaphrodite, self incompatible | | Campbell et al., 1991; Kausch-Blecken von Schmeling, 1992 |
| *Spondias ssp.,mainly S.mombin, S.tuberosa* | Hog plum,Mombin | | **little** | | **0.05** | | honey bees (*Apis mellifera*), stingless bess (*Melipona sp.*) | | wind pollinated | | hermaphrodite,varying degree of selfincompatibility depending on species and varieties | | Dominguez Sanchez et al., 2002 |
| *Tamarindus indica* | Tamarind | | **little** | | **0.05** | | honey bees (*Apis dorsata*) | | passive self-pollination | | hermaphrodite | | Free, 1993 |
| *Vaccinium corymbosum, V.angustifolium, V. ashei, V.myrtillus* | Highbush, blueberry,Lowbush blueberry, Rabbiteye, blueberry, Bilberry | | **great** | | **0.65** | | honey bees (*Apis mellifera*), bumble bees (*Bombus impatiens*), solitary bees (*Anthophora pilipes, Colletes sp., Habropoda laboriosa, Osmia ribifloris, O. lignaria*) | | passive self-pollination | | hermaphrodite, self-compatible, with varying, degree of selfincompatibility, buzz-pollination | | Payne et al., 1989; Cane & Payne, 1990; Free, 1993; Dogterom, 1999; Stubbs & Drummon, 1999, 2001; Dogterom et al. 2000, Sampson & Cane, 2000; Delaplane & Mayer, 2000; Hokanson & Hancock, 2000; Aras et al., 1996; Cane, 1997; Javorek et al., 2002; Dedej & DeDelaplane, 2003; Sampson et al., 2004, Desjardins & De Oliveira 2006 |
| *Vaccinium macrocarpon, V. oxycoccus* | American cranberry, European cranberry | | **great** | | **0.65** | | honey bees (*Apis mellifera*), bumble bees (*Bombus affinis*), solitary bees (*Megachile (Delomegachile*) addenda, (*M. rotundata*) | | passive self-pollination | | hermaphrodite, self-compatible | | Free, 1993; Cane et al., 1996; Delaplane & Mayer, 2000; Cane & Schiffhauer, 2003; Brown & McNeil, 2006; Evans & Spivak, 2006 |
| *Vitis vinifera* | Table grape, Vine grape | | **no increase** | | **0** | | honey bees, solitary bees | | passive self-pollination, wild pollination | | hermaphrodite, self-compatible | | Free, 1993; Rhodes, 2002; but production increase in *V. rotundifolia* (see Sampson et al., 2001) |
| *Zizyphus jujuba* | Jujube | | **modest** | | **0.25** | | honey bees (*Apis mellifera*) flies, beetles, wasps | | passive self-pollination | | hermaphrodite, self-compatible | | Free, 1993; Sharma & Jindal, 1997 |
| **Nut crops** | | | | | | | | | | | | | |
| *Amygdalus communis* | Almond | | **great** | | **0.65** | | honey bees (*Apis mellifera*), bumble bees, solitary bees (*Osmia cornuta*), flies | | passive self-pollination | | hermaphrodite, self-incompatible, but some new varieties self-compatible | | Free, 1993; Bosch, 1994; Bosch & Blas, 1994; Torre Grossa et al., 1994; Delaplane & Mayer, 2000; Westerkamp & Gottsberger, 2000; De Grandi-Hoffman, 2001; Thomson & Goodell, 2001; Soltész et al., 2003a; Lumkin, 2005 |
| *Anacardium occidentale* | Cashew nut, Cashewapple | | **great** | | **0.65** | | honey bees (*Apis dorsata, Apis mellifera*), stingless bees, bumble bees, solitary bees (*Centris analis*), butterflies, flies, hummingbirds | | passive self-pollination | | andro-monoecious | | Heard et al., 1990; Crane, 1991; Free, 1993; Freitas & Paxton, 1998; De Holanda- Neto et al., 2002; Freitas et al., 2002; Bhattacharya, 2004 |
| *Arachis hypogea* | Peanut, Groundnut | | **little** | | **0.05** | | honey bees (*Apis dorsata, Apis florae, Apis mellifera*), bumble bees, solitary bees, hover flies, butterflies | | self-pollination (many varieties cleistogamous) | | hermaphrodite, self-compatible | | Crane, 1991; Free, 1993; DR varies among varieties and no benefit found by Blanche et al. (2006) because of infrequent flower visitation |
| *Bertholletia excelsa* | Brazil nut, Para nut, Cream nut | | **essential** | | **0.95** | | bumble bees, solitary bees (*Euglossini, Xylocopa sp*.) | |  | | hermaphrodite, self- incompatible | | O’Malley et al., 1988; Mori & Prance, 1990; Free, 1993; Mauè,s 2002 |
| *Castanea sativa* | Chestnut | | **modest** | | **0.25** | | honey bees, solitary bees | | wind pollination | | monoecious, largely self- incompatible | | Manino et al., 1991; De Oliveira et al., 2001 |
| *Macadamia ternifolia* | Macadamia | | **essential** | | **0.95** | | honey bees (*Apis mellifera*), stingless bees (*Trigona carbonaria*), solitary bees (*Homalictus sp.),* wasps, butterflies | |  | | hermaphrodite, largely self- incompatible | | Free, 1993; Heard, 1993; Heard, 1994; Wallace et al., 1996; Blanche et al., 2006b |
| **Edible oil and proteinaceous crops** | | | | | | | | | | | | | |
| *Brassica alba, B. hirta, Sinapis alba, B. nigra, Sinapis nigra* | Mustard seeds | | **modest** | | **0.25** | | honey bees (*Apis mellifera*), solitary bees (*Osmia cornifrons, O. lignaria lignaria*) | | passive selfpollination, wind pollination | | hermaphrodite, self-compatible | | Free, 1993; Abel & Wilson, 1999; Abel et al., 2003 |
| *Brassica napus oleifera* | Rapeseed, Oilseed rape | | **modest** | | **0.25** | | honey bees (*Apis mellifera*), bumble bees, solitary bees (*Andrena sp., Osmia cornifrons, Osmia lignaria lignari, Halictus sp., Bombus sp.),* hoverflies | | passive selfpollination, wind pollination | | hermaphrodite, self-compatible | | Free, 1993; Adegas & Noqueira Couto, 1992; Abel & Wilson, 1999; Bürger, 2004; Manning & Boland, 2000; Abel et al., 2003; Morandin & Winston, 2005 |
| *Brassica rapa (formerly B. campestris)* | Turnip rape, Canola | | **great** | | **0.65** | | honey bees (*Apis cerana, A. florea, A. mellifera*), solitary bees (*Andrena ilerda, O. cornifrons, O.lignaria lignari, Halictus spp.*), flies (*Eristalis spp., Trichometallea pollinosa*) | | passive self- pollination, wind pollination | | hermaphrodite, largely self- incompatible | | Crane, 1991; Free, 1993; Schittenhelm et al., 1997; Abel & Wilson, 1999; Delaplane & Mayer, 2000; Westcott & Nelson, 2001; Abel et al., 2003 |
| *Carthamus tinctorius* | safflower | | **little** | | **0.05** | | honey bees (*Apis cerana, A. mellifera)*, solitary bees | | variable passive self-pollination | | hermaphrodite, self-compatible | | Crane, 1991; Free, 1993; Dajue & Mündel, 1996 |
| *Cocos nucifera* | Coconut monoecious, | | **modest** | | **0.25** | | honey bees, stingless bees | | passive self- pollination, little wind pollination | | partially self- compatible | | Free, 1993; Da Conceicao et al., 2004; Meléndez-Ramírez et al., 2004 |
| *Elaeis guineensis* | Oil palm | | **little** | | **0.05** | | weevils (*Elaeidobius sp*.), thrips (*Thrips hawaiiensis*) | | wind pollination | | monoecious | | Free, 1993; Dhileepan, 1994; Westerkamp & Gottsberger, 2000; Tandon et al., 2001; Krantz & Poinar, 2004; Mayfield, 2005 (but no effects of forest distance on pollination) |
| *Glycine max, G. soja* | Soybean | | **modest** | | **0.25** | | honey bees (*Apis mellifera*), bumble bees, solitary bees, (*Megachile rotundata*) | | passive self- pollination (most varieties cleistogamous) | | hermaphrodite, self-compatible | | Koelling et al., 1981; Free 1993; Moreti et al., 1998; Nogueira-Couto et al., 1998 for *G. wightii*; Chiari et al., 2005a, b; DR varies greatly among varieties, no benefit found by Ray et al. (2003) and some studies in Free (1993) |
| *Gossypium hirsutum, G. barbadense, G. arboreum, G. herbaceum* | Seedcotton | | **modest** | | **0.25** | | honey bees (*Apis sp*.), bumble bees (*Bombus sp*.), solitary bees (mainly *Xylocopa sp.),* wasps | | passive self- pollination | | hermaphrodite, self-compatible | | Free, 1993; Rhodes, 2002 |
| *Helianthus annuus* | Sunflower seeds | | **modest** | | **0.25** | | honey bees (*Apis cerana, A. mellifera*), bumble bees, solitary bees, stingless bees (*Trigona iridipennis*) | | passive self- pollination, but very low | | dichogamous, variable level of self-compatibility among varieties | | Bichee & Sharma, 1988; Crane, 1991; Free, 1993; DeGrandi-Hoffman & Martin, 1993; Moreti et al., 1996; Heard, 1999; DeGrandi-Hoffman & Watkins, 2000; Dag et al., 2002; Greenleaf, 2005; Greenleaf & Kremen, 2006b |
| *Linum usitatissimum* | Flaxseed | | **little** | | **0.05** | | honey bees (*Apis sp*.), bumble bees | | passive self- pollination, wind pollination | | hermaphrodite, self-compatible | | Free 1993 |
| *Olea europaea* | Olive | | **no increase** | | **0** | | honey bees visit flowers occasionally | | passive self- pollination, wind pollination | | andro- monoecious, variable level of self-incompatibility among varieties | | Free, 1993; Singh, 1997 (differ greatly among varieties); some authors classified olives to have little benefit according to Griggs et al. (1975) |
| *Sesamum indicum* | Sesame | | **modest** | | **0.25** | | honey bees (*Apis cerana, A. mellifera*), solitary bees, wasps, flies | | passive self- pollination | | hermaphrodite, self-compatible | | Free, 1993; Crane, 1991 |
| *Vicia faba* | Broad bean, Faba bean, Field bean, Horse bean | | **modest** | | **0.25** | | honey bees (*Apis mellifera*), bumble bees (*Bombus lapidarius, B. pascuorum, B. hortorum*), solitary bees (*Anthophora plumipes, Eucera spp., Megachile rotundata*) | | variable level of passive self- pollination among varieties | | hermaphrodite, self-compatible | | Free, 1993; Le Guen et al,. 1993; Suso et al., 1996; Bond & Kirby ,1999; Pierre et al., 1999; Somerville, 1999 |
| *Vitellaria paradoxa (syn. Butyrospermum paradoxum)* | karite nuts, Sheanuts | | **modest** | | **0.25** | | honey bees (*Apis mellifera adansonii*) | |  | | hermaphrodite | | Millogo-Rasolodimby, 1989; Kelly et al., 2004; Sanou et al., 2005; Tchuenguem Fohouo et al., 2005 |
| **Stimulant crops** | | | | | | | | | | | | | |
| *Coffea arabica,C. canephora* | Coffee | | **modest** *C. canephora* classified in **great** | | **0.25** | | honey bees (*Apis dorsata A. mellifera,*), stingless bees (Trigona (*Lepidotrigona) terminata)*, solitary bees (*Creightonella frontalis, Xylocopa (Zonohirsuta dejeanii*) | | passive self-pollination (mainly *C. arabica*), windpollination (mainly *C. canephora*) | | hermaphrodite, variable level of self-compatibility | | Free, 1993; Malerbo-Souza & Nogueira-Couto, 1997; Manrique & Thimann, 2002; Roubik, 2002a, b; Klein et al., 2003a, b, c; De Marco & Coelho, 2004; Ricketts et al., 2004; Ricketts, 2004 |
| *Cola nitida, C. vera, C. acuminata* | Cola nut, Kola nut | | **great (indirect)** | | **0.65** | | flies | |  | | hermaphrodite, andromonoecious, self-incompatible | | McGregor, 1976; Jacob, 1980; Osei, 1995, 1996 |
| *Theobroma cacao* | Cocoa | | **essential** | | **0.95** | | bees, cecidomyiid, midges, ceratopogonid midges | |  | | hermaphrodite, variable level of self-incompatibility (self-compatible in the amelonado varieties) | | Free, 1993; Falque et al., 1995, 1996; Lachenaud, 1994 |
| **Spices and condiments** | | | | | | | | | | | | | |
| *Afromomum melegueta* | Grains of paradise | | **unknown** | |  | | unknown | | unknown | | unknown | | unknown |
| *Carum carvi* | Caraway | | **modest** | | **0.25** | | solitary bees, flies (Ricciardelli D’Albore, 1986) | | wind pollination, little passive selfpollination | | andromonoecious, dichogamous, self-compatible | | Bouwmeester et al., 1995; Bouwmeester & Smid, 1995; Németh et al., 1999, Németh & Székely, 2000; Langenberger & Davis, 2002 |
| *Coriandrum sativum* | Coriander | | **great** | | **0.65** | | honey bees (*Apis cerana, A. dorsata, A. florea, A. mellifera)*, stingless bees, solitary bees | | passive self- pollination | | hermaphrodite, self-compatible | | Crane, 1991; Free, 1993; Koul et al., 1993; Diederichsen, 1996 |
| *Cuminum cyminum* | Cumin | | **great** | | **0.65** | | unknown | | wind pollination, little passive self- pollination | | hermaphrodite, self-compatible | | Free, 1993 |
| *Elettaria cardamomum* | Cardamom | | **great** | | **0.65** | | honey bees (*Apis cerana, Apis dorsata, Apis florea*), solitary bees | | passive self- pollination | | hermaphrodite | | Crane, 1991; Free, 1993; Sasikumar et al., 1999 |
| *Illicium verum* | Star anise | | **unknown** | |  | | unknown | | unknown | | hermaphrodite, self-incompatible | | unknown |
| *Foeniculum vulgare* | Fennel seed | | **great** | | **0.65** | | honey bees (*Apis florea, A. mellifera)* | | wind pollination, little passive self- pollination | | hermaphrodite, few andro- monoecious, dichogamous, self-incompatible | | McGregor, 1976; Free, 1993; Koul et al., 1993; Németh et al., 1999; Falzari et al., 2005 |
| *Myristica fragrans* | Nutmeg | | **great (indirect)** | | **0.65** | | beetles (*Formicomus braminus*) | | wind pollination | | dioecious | | Armstrong & Drummond, 1986 |
| *Pimenta dioica (syn. P. officinalis, P. dioica)* | Allspice, Pimento | | **great (indirect)** | | **0.65** | | honey bees, *Halictus, Exomalopsis, Ceratina* | | unknown | | dioecious | | Free, 1993; Lughadha & Proenca, 1996 |
| *Piper nigrum, P.longum* | Pepper | | **no increase** | | **0** | | bees, hover flies as flower visitors | | passive self-pollination, wind pollination | | hermaphrodite, self-compatible, dichogamous | | Free, 1993; but insect pollination mentioned in Roubik (1995); Sargent & Otto, 2004 |
| *Pimpinella anisum* | Anise | | **unknown** | |  | | honey bees (*Apis mellifera*), solitary bees, flies | | passive self- pollination, wind pollination | | hermaphrodite | | McGregor, 1976; Ricciardelli D’Albore, 1986 |
| *Vanilla planifolia, V. pompona* | Vanilla | | **essential** | | **0.95** | | stingless bees, solitary bees, hummingbirds | | hand pollination | | hermaphrodite, self-incompatible | | little natural pollination (<1%) in Free, 1993 |

**REFERENCES**

Klein A-M, Vaissière BE, Cane JH, Steffan-Dewenter I, Cunningham SA, Kremen C, Tscharntke T. 2007. Importance of pollinators in changing landscapes for world crops. Proceedings of the Royal Society B: Biological Sciences **274**:66, 95–96

Abbo, S., Shtienberg, D., Lichtenzveig, J., Lev-Yadun, S. & Gopher, A. 2003 The chickpea, summer cropping, and a new model for pulse domestication in the ancient Near East. Quart. Rev. Biol. 78, 435-448.

Abel, C. A., Wilson, R. L. & Luhmann, R. L. 2003 Pollinating efficacy of Osmia cornifrons and Osmia lignaria subsp. lignaria (Hymenoptera: Megachilidae) on three Brassicaceae species grown under field cages. J. Kans. Entomol. Soc. 38, 545-552.

Abel, C. A. & Wilson, R. L. 1999 The use of diverse plant species for increasing Osmia cornifrons (Hymenoptera: Megachilidae) in field cages. J. Kansas Entomol. Soc. 71, 23-28.

Abraham, K. & Gopinathan Nair, P. 1990 Floral biology and artificial pollination in Dioscorea alata L. Euphytica 48, 45-51.

Adegas, J. E. B. & Nogueira Couto, R. H. 1992 Entomophilous pollination in rape (Brassica napus L. var. oleifera) in Brazil. Apidologie 23, 203-209.

Akoroda, M. O. 1983 Floral biology in relation to hand pollination of white yam. Euphytica 32, 831-838.

Allan, R. E. 1980 Wheat. In Hybridization of crop plants (ed. W. R. Fehr & H. H. Hadley) pp. 709-720. American Society of Agronomy & Crop Science Society of America, Madison, WI, USA.

Almanza, M. T., Cure, J. R., Rojas, D. & Wittmann, D. 2006 A model to pollinate an exotic fruit (Solanum quitoense) with bumlebees. 53 Jahrestagung der Arbeitsgemeinschaft der Bieneninstitute Hohenheim, Germany.

Almeida Lima, A. de 2002 Maracujá Produção. Embrapa Informação Tecnológica, p. 104 Brasília, Brazil.

Aras, P., De Oliveira, D. & Savoie, L. 1996 Effect of a honey bee (Hymenoptera : Apidae) gradient on the pollination and yield of lowbush blueberry. J. Econ. Entomol. 89, 1080-1083.

Armstrong, J. E. & Drummond, B. A. III 1986 Floral biology of Myristica fragrans Houtt. (Myristicaceae), the nutmeg of commerce. Biotropica 18, 32-38.

Asada, S. & Ono, M. 1996 Crop pollination by Japanese bumblebees, Bombus spp. (Hymenoptera: Apidae): tomato foraging behaviour and pollination efficiency. Appl. Entomol. Zool. 31, 581-586.

Asworth, L. & Galetto, L. 2001 Pollinators and reproductive success of the wild cucurbit Cucurbita maxima ssp. andreana (Cucurbitaceae). Plant Biology 3, 398-404.

Austin, P. T., Hewett, E. W., Noiton, D. A. & Plummer, J. A. 1996 Cross pollination of ‚Sundrop’ apricot (Prunus armeniaca L.) by honeybees. New Zeal. J. Crop Hort. 24, 287-294.

Awasthi, R. P. & Kumar, S. 1997 Kiwifruit. In Fruit crop pollination (ed. L. R. Verma & K. K. Jindal) pp. 227- 240. Kalyani Pub., Ludhiana, India.

Banga, O. 1961 Breeding Scorzonera hispanica L. by the polycross method. Euphytica 10, 49-58.

Batra, S. W. T. 1998 Hornfaced bees for apple pollination. Am. Bee J. 138, 361. 16

Bauwmeester, H. J. & Smid, H. G. 1995 Seed yield in caraway (Carum carvi). 1. Role of pollination. J Agric. Sci. 124, 235-244.

Bouwmeester, H. J., Smid, H. G. & Loman, E. 1995 Seed yield in caraway (Carum carvi). 2. role of assimilate availability. J. Agric. Sci. 124, 245-251.

Bell, M. C., Spooner-Hart, R. N. & Haigh, A. M. 2006 Pollination of greenhause tomatoes by the Australian bluebanded bee Amegilla (Zonamegilla) holmesi (Hymenoptera: Apidae). J. Econ. Entomol. 99, 437-442.

Benedek, P., Erdös, Z., Skóla, I., Nyéki, J. & Szalay, L. 2006 The effect of reduced bee pollination period to the fruit set of apricots. Acta Hort. 701, 723-726. Bhatia, R., Gupta, D., Chandel, J. S. & Sharma, N. K. 1995 Relative abundance of insect visitors on flowers of major subtropical fruits in Himachal Pradesh and their effect on fruit set. Indian J. Agr. S. 65, 907-912.

Bhattacharya, A. 2004 Flower visitors and fruit set of Anacardium occidentale. Ann. Bot. Fenn. 41, 385-392.

Bichee, S. L. & Sharma, M. 1988 Effect of different modes of pollination in sunflower Helianthus annuus L. (Compositae) sown on different dates by Trigona iridipennis Smith. Apiacta 23, 65-68.

Bixby, P. J. & Levin, D. A. 1996 Response to selection on autogamy in Phlox. Evolution 50, 892- 899.

Björkman, T. 1995a The effectiveness of heterostyly in preventing illegitimate pollination in dish-shaped flowers. Sex. Pl. Repro. 8, 143-146.

Björkman T. 1995b Role of honey bees (Hymenoptera: Apidae) in the pollination of buckwheat in eastern North America. J. Econ. Entomol. 88,1739-1745. Blanche, R. & Cunningham, S. A. 2005 Rain forest provides pollinating beetles for atemoya crops. J. Econ. Entomol. 98, 1193-1201.

Blanche, K. R., Hughes, M., Ludwig, J. A. & Cunningham, S. A. 2006a Do flower-tripping bees enhance yields in peanut varieties grown in north Queensland? Aust. J. Exp. Agr. in press.

Blanche, K. R., Ludwig, J. A & Cunningham, S. A. 2006b Proximity to rainforest enhances pollination and fruit set in macadamia and longan orchards in north Queensland, Australia. J. Appl. Ecol. in press.

Bolli, R. 1994 Revision of the genus Sambucus. Dissertationes Botanica 223, 1-227.

Bond, D. A. & Kirby, E. J. M. 1999 Anthophora plumipes (Hymenoptera: Anthophoridae) as a pollinator of broad bean (Vicia faba major) J. Api. Res. 38, 199-203. Bosch, J. 1994 The nesting behavior of the mason bee Osmia cornuta (Latr.) with special reference to its pollinating potential (Hymenoptera, Megachilidae). Apidologie 25, 84-93.

Bosch, J. & Blas, M. 1994 Foraging behavior and pollinating efficiency of Osmia cornuta and Apis mellifera on almond (Hymenoptera, Megachilidae and Apidae. Appl. Entomol. Zool. 29, 1-9.

Bosch, J. & Kemp, W. P. 1999 Exceptional cherry production in orchard pollinated with blue orchard bees. Bee World 80, 163-173.

Bosch, J., Kemp, W. P. & Trostle, G. E. 2006 Bee population returns and cherry yields in an orchard pollinated with Osmia lignaria (Hymenoptera: Megachilidae). J. Econ. Entomol. 99, 408-413.

Bouwmeester, H. J. & H. G. Smid. 1995 Seed yield in caraway (Carum carvi). 1. role of pollination. J. Agric. Sci. 124, 235-244.

Brancher, A. 1999 Estudo do fornecimento de fontes protéicas em substituição ao pólen para abelhas (Apis mellifera). Reunião Técnica de Pesquisa em Maracujazeiro 97.

Brantjes, N. B. M. 1981 Nectar and the pollination of bread fruit, Artocarpus altilis (Moraceaae) Acta Bot. Neerl. 30, 345-352.

Brown, O. A. & McNeil, J. N. 2006 Fruit production in cranberry (Ericaceae: Vaccinium macrocarpon): a bethedging strategy to optimize reproductive effort. Am. J. Bot. 93, 910-916.

Brown, C. M. 1980 Oat. In Hybridization of crop plants (ed. W. R. Fehr & H. H. Hadley) pp. 427-441. American Society of Agronomy & Crop Science Society of America, Madison, WI, USA.

Bruckner, C. H., Casali, V. W. D., de Moraes, C. F., Ragazzi, A. J., & da Silva, E. A. M. 1995 Selfincompatibility in passion fruit (Passiflora edulis Sims). Acta Hort. 370, 45-57.

Bürger, C. 2004 Effects of landscape structure on bee diversity and pollination at different spatial scales. PhD thesis. Institute of Agroecology, University of Göttingen, Germany.

Burton, G. W. 1980. Pearl millet. In Hybridization of crop plants (ed. W. R. Fehr & H. H. Hadley) pp. 457-469. American Society of Agronomy & Crop Science Society of America, Madison, WI, USA.

Calzoni, G. L. & Speranza, A. 1998 Insect controlled pollination in Japanese plum (Prunus salicina Lindl.). Sci. Hortic. 72, 227-237.

Camillo, E. 1996 Polinizacção do maracujá amarelo. Congresso Braileio de Apicoltura 11, 317-321.

Campbell, C.G. 1997 Buckwheat Fagopyrum esculentum Moench. Institute of Plant Genetics and crop Plant Research, Gatersleben / International Plant genetic Resoruces Institute, Rome, Italy. 95 pp.

Campbell, C. S., Greene, W. C. & Dickinson, T. A. 1991 Reproductive biology in Subfam. Maloideae (Rosaceae). Syst. Bot. 16, 333-349.

Can-Alonso, C., Fquezada-Euán, J. J. G., Xiu-Ancona, P., Moo-Valle, H., Valdovinos-Nunez, G. R., MedinaPeralta, S. 2005 Pollination of ‘criollo’ avocados (Persea americana) and the behaviour of associated bees in subtropical Mexico. J. Apic. Res. 44, 3-8.

Cane, J. H. 1997 Lifetime monetary value of individual pollinators: the bee Habropoda laboriosa at rabbiteye blueberry (Vaccinium ashei Reade). Acta Hort. 446, 67-70.

Cane, J. H. 2005 Pollination potential of the bee Osmia aglaia for cultivated red raspberries and blackberries (Rubus: Rosaceae). HortSci. 40, 1705-1708. Cane, J. H. & Payne, J. A. 1990 Native bee pollinates rabbiteye blueberry. Highlights of Agricultural Research 37, 4.

Cane, J. H. & Schiffhauer, D. 2001 Pollinator genetics and pollination: do honey bee colonies selected for pollen-hoarding field better pollinators of cranberry Vaccinium macrocarpon? Ecol. Entomol. 26, 117-123.

Cane, J. H. & Schiffhauer, D. 2003 Dose response relationships between pollination and fruiting refine pollinator comparisons for cranberry (Vaccinium macrocarpon [Ericaceae]). Am. J. Bot. 90, 1425-1432. Cane, J. H., Schiffhauer, D. & Kervin, L. J. 1996 Pollination, foraging, and nesting ecology of the leaf-cutting bee Megachile (Delomegachile) addenda (Hymenoptera: Megachilidae) on cranberry beds. Ann. Entomol. Soc. Am. 89, 361-367.

Canto-Aguilar, A. & Parra-Tabla, V. 2000 Importance of conserving alternative pollinators: assessing the pollination efficiency of the squash bee, Peponapis limitaris in Cucurbita moschata (Cucurbitaceae). J. Ins. Conserv. 4, 203-210.

Cardoso, A. I. I. 2003 Seed yield and quality in response to pollen load of squash cv. Piramoita. Bragantia 62, 47-52.

Carreck, N. L. & Williams, H. 1998 The economic value of bees in the UK. Bee World 79, 115-123.

Cavers, P. B. & Harper, J. L. 1964 Rumex obtusifolius L. and R. crispus L. J. Ecol. 52, 737-766.

Cauich, O., Quezada-Euan, J. J. G., Marcias-Marcias, J. O., Reyes-Oregel, V., Medina-Peralta, S. & ParraTabla, V. 2004 Behavior and pollination efficiency of Nannotrigona perilampoides (Hymenoptera: Meliponini) on greenhouse tomatoes (Lycopersicon esculentum) in stubropical Mexico. J. Econ. Entomol. 97, 475-481.

Chagnon, M., Gingras, J. & De Oliveira, D. 1991 Honey bee (Hymenoptera: Apidae) foraging behaviour and raspberry pollination. J. Econ. Entomol. 84, 457-460.

Chagnon, M., Gingras, J. & De Oliveira, D. 1993 Complementary aspects of strawberry pollination by honey and indigenous bees (Hymenoptera). J. Econ. Entomol. 86, 416-420.

Chacoff, N. P. 2007 Los ecosistemas naturales como fuente de polinizadores para cultivos en el pedemonte de las yungas. PhD Thesis. Universidad Nacional del Comahue. Argentina in press.

Chacoff, N. P. & Aizen, M. A. 2006 Edge effects on flower-visiting insects in grapefruit plantations bordering premontane subtropical forest. J. Appl. Ecol. 43, 18-27.

Chiari, W. C., de Alencar Arnaut de Toledo, V., Ruvolo-Takasusuki, M. C. C., Attencia, V. M., Costa, F. M., Kotaka, C. S., Sakaguti, E. S. & Magalhaes, H. R. 2005a Floral biology and behavior of Africanized honeybees Apis mellifera in soybean (Glycine max L. Merril). Braz. Arch. Biol.Technol. 48, 367-378.

Chiari, W. C., de Alencar Arnaut de Toledo, V., Ruvolo-Takasusuki, M. C. C., de Oliveira, A. J. B., Sakaguti, E. S., Attencia, V. M., costa, F. M. & Mitsui, M. H. 2005b Pollination of soybean (Glycine max L. Merril) by honey bees (Apis mellifera L.). Braz. Arch. Biol.Technol. 48, 31-36.

Corbet, S. A. & Willmer, P. 1980a Passiflora and Xylocopa: economic and evolutionary considerations. Acta Bot. Neerl. 29, 55.

Costa, G., Testolin, R. & Vizzotto, G. 1993 Kiwifruit pollination: an unbiased estimate of wind and bee contribution. New Zeal. J. Crop Hort. 21, 189-195.

Crane, E. 1991 Apis species of tropical Asia as pollinators and some rearing methods for them. Acta Hort. 288, 29-48.

Crossa-Raynaud, P. 1984 Quelques productions fruitières dépendant d'une pollinisation anémogame: noyer, noisetier, olivier, palmier-dattier, pistachier. In Pollinisation et productions végétales (ed. Pesson, P. & J. Louveaux). INRA, Paris, France.

Da Conceicao, E. S., Delabie, J. H. C. & De Costa Neto, A. 2004 The entomophily of the coconut tree in question: The evaluation of pollen transportation by ants (Hymenoptera: Formicidae) and bees (Hymenoptera: Apoidea) in inflorescence. Neotropical Entomol. 33, 679- 683.

Dag, A. & Kammer, Y. 2001 Comparison between the effectiveness of honey bee (Apis mellifera) and bumble bee (Bombus terrestris) as pollinators of greenhouse sweet pepper (Capsicum annuum) Am. Bee J. 141, 447-448.

Da Mota, M. O. S. & Nogueira-Couto, R. H. 2002 Entomophilous pollination in peach (Prunus persica L.). Braz. J. Vet. Res. Anim. Sci. 39, 124-128.

Da Silva, M. A. E., Bruckner, C. H. , Picanço, M. & Molina-Rugama, A. 1999 Número floral, clima, densidad poblacional de Xylocopa spp. (Hymenoptera: Anthophoridae) y polinización del maracuyá (Passiflora edulis f. flavicarpa). Rev. Biol. Trop. 47, 711-718.

Dag, A., Degani, C. & Gazit, S. 2001 In-hive pollen transfer in mango. Acta Hort. 561, 61-65.

Dag, A., Lior, E. & Afik, O. 2002 Pollination of confection sunflowers (Helianthus annuus L.) by honey bees (Apis mellifera). Am. Bee J. 142, 443- 445.

Dajue, L, Mündel, H.H. 1996 Safflower Carthamus tinctorius L. Institute of Plant Genetics and crop Plant Research, Gatersleben / International Plant genetic Resoruces Institute, Rome, Italy. 83 pp.

Davenport, T. L. & O'Neal, J. T. 2000 Flowering and fruit set of mamey sapote [Calocarpum sapota (jacq.) Merr.] cv. Magaña in south Florida. Scientia Hort. 83, 61-70.

Dedej, S. & Delaplane, K. S. 2003 Honey bee (Hymenoptera: Apidae) pollination of rabbiteye blueberry Vaccinium ashei var. 'Climax' is pollinator density-dependent. J. Econ. Entom. 96, 1215-1220.

Dedej, S. & Delaplane, K. S. 2004 Nectar-robbing carpenter bees reduce seed-setting capability of honey bees (Hymenoptera: Apidae) in rabbiteye blueberry, Vaccinium ashei, 'Climax'. Environ. Entomol. 33, 100-106.

DeFelice, M. S. 2004 Prickly pear cactus, Opuntia spp. - a spine-tingling tale. Weed Technol. 18, 869-877.

Degen, B., Caron, H., Bandou, E., Maggia, L., Chevallier, M-H., Leveau, A. & Kremer A. 2001 Fine-scale spatial genetic structure of eight tropical tree species analysed by RAPDs. Heredity 87, 497-507.

Degenhardt, J., Orth, A. I. & Guerra, M. P. et al. 2001 Flower morphology of Feijoa (Feijoa sellowiana) and it’s implications of pollination. Rev. Bras. Frutic. 23, 718-721.

De Grandi-Hoffman, G. 2001 The pollination of almonds. Am. Bee J. 141, 655-657.

De Grandi-Hoffman, G. & Martin, J. H. 1993 The size and distribution of the honey bee (Apis mellifera L.) cross-pollinating population on male sterile sunflowers (Helianthus annuus L.). J. Apic. Res. 32, 135-142.

De Grandi-Hoffman, G. & Watkins, J. C. 2000 The foraging activity of honey bees Apis mellifera and non-Apis bees on hybrid sunflowers (Helianthus annuus) and its influence on cross-pollination and seed set. J. Apic. Res. 39, 37-45.

De Holanda-Neto, J. P., Freitas, B. M., Bueno, D. M. & De Aranjo, Z. B. 2002 Low seed/ nut productivity in cashew (Anacardium occidentale): Effects of self- incompatibility and honey bee (Apis mellifera) foraging behaviour. J. Hort. Sci. Biotech. 77, 226-231.

Delaplane, K. S. & Mayer, D. F. 2000 Crop pollination by bees. CABI Publishing, New. York, USA.

De Lozano, N. B. 1986 Contribution to the study of the floral anatomy and pollination of the hog plum (Spondias mombin L.). Caldasia 15, 369-380.

De Marco Jr., P. & Coelho, F. M. 2004 Services performed by the ecosystem: forest remnants influence agricultural culture pollination and production. Biodivers. Conserv. 13, 1245-1255.

De Oliveira Cruz, D., Freitas, B. M., Da Silva, L. A., da Silva, E. M. S., Bomfim, I. G. A. 2005 Pollination efficiency of the stingless bee Melipona subnitida on greenhouse sweet pepper. Presq. Agropec. Bras. 40, 1197-1201.

De Oliveira, D., Gomes, A., Ilharco, F. A., Manteigas, A. M., Pinto, J. & Ramalho, J. 2001 Importance of insect pollinators for the production of the chestnut Castanea sativa. Acta Hort. 561, 269-273.

Devy, M. S. & Davidar, P. 2003 Pollination systems of trees in Kakachi, a mid-elevation wet evergreen forest of Western Ghats, India. Am. J. Bot. 90, 650-657 (supporting data <http://ajbsupp.botany.org/v90/>).

Derin, K. & Eti, S. 2001 Determination of pollen quality, quantity and effect of cross pollination on the fruit set and quality in the pomegranate. Turk. J. Agric. For. 25, 169-173.

Desjardins, È.-C & De Oliveira, D. 2006 Commercial bumblee bee Bombus impatiens (Hymenoptera: Apidae) as a pollinator in lowbush blueberry (Ericale : Ericaceae) fields. J. Econ. Entomol. 99, 443-449.

Dhileepan, K. 1994 Variation in popuations of the introduced pollinating weevil (Elaeidobius kamerunicus) (Coleptera; Curculionidae) and its impact on fruit set of oil palm (Elaeis guineensis) in India. Bull. Entomol. Res. 84, 477-485.

Diederichsen, A. 1996 Coriander Coriandrum sativum L. 82 pp. Institute of Plant Genetics and crop Plant Research, Gatersleben / International Plant genetic Resoruces Institute, Rome, Italy.

Dogterom, M. H. 1999 Pollination by four species of bees on highbush blueberry. Ph.D. Thesis. Simon Fraser Univ., Burnaby, B.C., Canada.

Dogterom, M. H., Winston, M. L. & Mukai, A. 2000 Effect of pollen load size and source (self, outcross) on seed and fruit production in highbush blueberry cv. 'Bluecrop' (Vaccinium corymbosum; Ericaceae). Am. J. Bot. 87, 1584-1591.

Doku, E.V. 1968 Flowering, pollination and pod formation in bambara groundnut (Voandzeia subterranea) in Ghana. Exp. Agr. 4, 41-48.

Dominguez Sanchez, D., Goulson, D., Serna Ramos, R. 2002 Stingless bees as alternative pollinators and their possible competition with Africanized bees in Tabasco, Mexico. Bees without frontiers: The Proceedings of the Sixth European Bee Conference IBRA, Cardiff, UK, 1-5 July 2002, pp. 128-133.

Dönmez, A. A. 2004 The genus Crataegus L. (Rosaceae) with special reference to hybridisation and biodiversity in Turkey. Turk. J. Bot. 28, 29-37.

Ducroquet, J. P. H. J. & Hickel, E. R. 1997 Birds as pollinators of feijoa (Acca sellowiana Bera). Acta Hort. 452, 37-40.

Dunthorn, M. 2004 Cryptic dioecy in Mammea (Clusiaceae). Plant Syst. Evol. 249, 191-196.

Du Toit, A. P. 1990 Pollination research: a missing link in subtropical fruit production. Acta Hort. 275, 239-243.

Du Toit, A. P. 1994 Pollination of avocados, mangos and litchis. Inligtingsbulletin Institut vir Tropiese en Subtropiese Gewasse 262, 7-8.

Eisikowitch, D., Dafni, A. & Ivri, Y. 1986 Reward-partitioning in Capparis spp. along ecological gradient. Oecologia 71, 47-50.

Ercan, N. & Onus, A. N. 2003 The effects of bumblebees (Bombus terrestris L.) on fruit quality and yield of pepper (Capsicum annuum L.) grown in an unheated greenhouse. Israel J. Plant Sci. 51, 275-283.

Erskine, W. & Muehlbauer, F. J. 1991 Allozyme and morphological variability, outcrossing rate and core collection formation in lentil germplasm. Theor. Appl. Genet. 83, 119-125.

Etoh, T. & Hong, C. J. 2001 RAPD markers for fertile garlic. Acta Hort. 555, 209-212.

Evans, E. C. & Spivak, M. 2006 Effects of honey bee (Hymenoptera: Apidae) and bumble bee (Hymeoptera: Apida) presence on cranberry (Ericales: Ericacaeae) pollination. J. Econ. Entomol. 99, 614-620.

Falque, M., Vincent, A., Vaissière, B. E. & Eskes, A. B. 1995 Effect of pollination intensity on fruit and seed set in Theobroma cacao L. Sex. Plant Reprod. 8, 354-360.

Falque, M., Lesdalons, C. & Eskes, A. B. 1996 Comparison of two cacao (Theobroma cacao L.) clones for the effect of pollination intensity on fruit set and seed content. Sex. Plant Reprod. 9, 221-227.

Falzari, L. M., Menary, R. C. & Dragar, V. A. 2005 Reducing fennel stand density increases pollen production, improving potential for pollination and subsequent oil yield. HortScience 40, 629-634.

FAOSTAT data 2005 Data available at http://faostat.fao.org; Agricultural data/Agricultural production/Crops primary. last accessed in July 2006.

Fourez, A. 1995 Impact of pollination by bees (Apis mellifera Linne) on the production of apples (CV Jonagold). Bel. J. Zool. 125, 135-141.

Franklin, G., Pius, P. K. & Ignacimuthu, S. 2000 Factors affecting in vitro flowering and fruiting of green pea (Pisum sativum L.) Euphytica 115, 65-74.

Free, J. B. 1993 Insect Pollination of Crops. Academic Press, London, UK.

Freitas, B. M. & De Oliveira Filho, J. H. 2003 Rational nesting box to carpenter bees (Xylocopa frontalis) in the pollination of passionfruit (Passiflora edulis). Cienc. Rural 33, 1135-1139.

Freitas, B. M. & Paxton, R. J. 1998 A comparison of two pollinators: the introduced honey bee Apis mellifera and an indigenous bee Centris tarsata on cashew Anacardium occidentale in its native range of NE Brazil. J. Appl. Biol. 35, 109-121.

Freitas, B. M., Paxton, R. J., de Holanda-Neto, J. P. 2002 Identifiying pollinators among an array of flower visitors, and the case of inadequate cashew pollination in NE Brazil (ed. P. G. Kevan & ImperatrizFonseca, V. L.), pp. 229-254. Ministry of Environment: Secretariat for Biodiversity and Forests, Brazil.

Frève, A., de Oliveira, D. & Gingras, J. 2001 Insect pollination and production in two old varieties of Damas Plums (Prunus domestica L.) Acta Hort. 561, 249-251.

Fuchs, R. & Müller, M. 2004 Pollination problems in styrain oil pumpkin plants: can bumblebees be an alternative to honeybees? Phton (Horn, Austria) 44, 155-165.

Galon, I., Gazit, S. & Podoler, H. 1982 Improvement of natural fruit set of Annona by increasing the population of pollinating insects. Alon Halontea 9, 611-614.

Garcia Neto, M., Couto, R. H. N., Malheiros, E. B. 1988 Polinização em Dolichos lab-lab. Ciência Zootécnica 3, 3-4.

Gazit, S. & Degani, C. 2002 Reproductive Biology. In The Avocado: Botany, Production and Uses. (ed. A. W. Whiley, B. Schaffer & B. N. Wolstenholme) pp. 101-133. CAB International, Wallingford.

Gazit, S., Galon, I. & Podoler, H. 1982 The role of nitidulid beetles in natural pollination of annona in Israel. J. Am. Soc. Hort. Sci 107, 849-852.

George, A. P., Nissen, R. J. & Campbell, J. A. 1992 Pollination and selection in Annona species (cherimoya, atemoya and sugar apple). Acta Hort. 321, 178-185.

George, A. P., Nissen, R. J., Ironside, D. A. & Anderson, P. 1989 Effects of nitidulid beetles on pollination and fruit set of Annona spp. hybrids. Sci. Hortic. 39, 289-299.

George, S. T., Pillai, K. R., Lim, K. H., Tham, S. & Zainal Abidin, M. 1994 Recent developments in assisted cross-pollination to enhance yield of durian clone D24. In Recent Developments in Durian Cultivation (ed. O. Mohamad, M. Zainal Abidin & M. O Shamsudin) pp. 63-70. MARDI. Serdang, Malysia. Gingras, D.,

Gingras, J. & De Olivera, D. 1999 Visits of honeybees (Hymenoptera: Apidae) and their effects on cucumber yields in the field. J. Econ. Entomol. 92, 435-438.

Gonzalez, M. V., Coque, M. & Herrero, M. 1998 Influence of pollination systems on fruit set and fruit quality in kiwifruit (Actinidia deliciosa). Ann. Appl. Biol. 132, 349- 355.

Goodman, R., Hepworth, G., Kaczynski, P., McKee, B., Clarke, S. & Bluett, C. 2001 Honeybee pollination of buckwheat (Fagopyrum esculentum Moench cv. Manor). Aust. J. Exp. Agr. 41, 1217-1221.

Gottsberger, G. 1999 Pollination and evolution in neotropical Annonaceae. Plant Species Biol. 14, 143-152.

Goubara, M. & Takasaki, T. 2004 Pollination effects of the sweat bee Lasioglossum villosulum trichopse (Hymenoptera : Halictidae) on genic male-sterile lettuce. Appl. Entomol. Zool. 39, 163-169.

Grant, V. & Hurd, P. D. 1979 Pollination of southwestern Opuntias. Plant. Syst. Evol. 133, 15-28.

Grau, A., Dueñas, R. O., Cabrera, C. N. & Hermann, M. 2003 Mashua Tropaeolum tuberosum Ruîz & Pav. Int. potato center, Lima, Peru, p.55. Plant genet. Resources Inst., Rome, Italy.

Greenleaf, S. A. & Kremen, C. 2006a Wild bee species increase tomato production and respond differently to surrounding land use in Northern California. Biol. Conserv. in press, available online. (doi:10.1016/j.biocon.2006.05.025)

Greenleaf, S. A. & Kremen, C. 2006b Wild bees enhance honey bees' pollination of hybrid sunflower. Proc. Nat. Acad. Sci. USA. 103, 13890-13895. (doi:10.1073/pnas.0600929103)

Grewal G. S., Singh G., & Kandoria J. L. 1990 Insect pollinators of pigeonpea (Cajanus cajan) around Ludhiana. Ind. J. Agric. Sci. 60, 227-228.

Griggs, W. H., Hartmann, H. T., Bradley, M. V., Iwakiri, B. T., Whisler, J. E. 1975 Olive pollination in California. Bul. Calif. Agr. Exp. Sta 869.

Gritton, E. T . 1980 Field pea. In Hybridization of crop plants. (ed. W. R. Fehr & H .H. Hadley) pp. 347-356. American Society of Agronomy & Crop Science Society fo America, Madison, WI, USA.

Gross, C. L. 1993 The reproductive ecology of Canavalia rosea (Fabaceae) on Anak Krakatau, Indonesia. Aust. J. Bot. 41, 591-599.

Hagedoorn, K., Gross, C. L., Sedgley, M. & Keller, M. A. 1996 Increased Tomato Yield Through Pollination by Native Australian Amegilla chlorocyanea (Hymenoptera: Anthophoridae). J. Econ. Entomol. 99, 828-833.

Hagerup, O. 1957 Wind autogamy in Arbutus. Bull. Jardin Botanique de l'Etat, Bruxelles 27, 41-47. Hamon, S. 1991 The reproductive biology of okra. 2. Self-fertilization kinetics in the cultivated okra (Abelmoschus esculentus), and consequences for breeding. Euphytica 53, 49-55.

Hasan, S. M. Z & Razak, A. R. 1992 Parthenocarpy in seedless breadfruit (Artocarpus incisus (Thunb. L.) Acta Hort. 321, 648-652.

Heard, T. A. 1993 Pollinator requirements and flowering patterns of Macadamia integrifolia. Aust. J. Bot. 41, 491-497.

Heard, T. A. 1994 Behaviour and pollinator efficiency of stingless bees and honey bees on macadamia flowers. J. Apic. Res. 33, 191-198.

Heard, T. A. 1999 The role of stingless bees in crop pollination. Ann. Rev. Entomol. 44, 183-206.

Heard, T. A., Vithanage, V. & Chacko, E. K. 1990 Pollination biology of cashew in the Northern Territory of Australia. Aust. J. Agric. Res. 41, 1101-1114.

Hedström, I. 1988 Pollen carriers and fruit development of Psidium guajava L. (Myrtaceae) in the neotropical region. Rev. Biol. Trop. 36, 551-553.

Heiser, C. B 1972 The relationships of the naranjilla, Solanum quitoense. Biotropica 4, 77-84.

Hennessy, E. F. F. 1991 Erythrinae (Fabacea) in Southern Africa. Bothalia 21, 1-25.

Hermann, M. 1997 Arracacha (Arracacia xanthorrhiza bancroft). In Andean roots and tubers: Ahipa, arracacha, maca and yacon (ed. H. Hermann & J. Heller) pp.75-172. Institute of Plant Genetics and crop Plant Research, Gatersleben / International Plant genetic Resoruces Institute, Rome, Italy.

Herrera, C. M., Herrera, J. & Espadaler, X. 1984 Nectar thievery by ants from southern Spanish insectpollinated flowers. Insectes Soc. 31, 142-154.

Higo, H. A., Rice, N. D., Winston, M. L. & Lewis, B. 2004 Honey bee (Hymeoptera: Apidae) distribution and potential for supplementary pollination in commercial tomato greenhouses during winter. J. Econ. Entomol. 97, 163-170.

Hokanson, K. & Hancock, J. 2000 Early-acting inbreeding depression in three species of Vaccinium (Ericaceae). Sex. Plant Reprod. 13, 145-150.

Hogendoorn, K., Steen, Z. & Schwarz, M. P. 2000 Native Australian carpenter bees as a potential alternative to introducing bumble bees for tomato pollination in greenhouses. J. Apic. Res. 39, 67-74.

Howard, H. W. & Lyon, A. G. 1952 Nasturtium officinale R. Br. (Rorippa nasturtium-aquaticum L. Hayek) J. Ecol. 40, 228-245.

Howpage, D., Spooner- Hart, R. N. & Vithanage, V. 2001 Influence of honey bee (Apis mellifera) on kiwifruit pollination and fruit quality under Australian conditions. New Zeal. J. Crop Hort. 29, 51-59.

Husin, A. & Abidin, M. Z. 1998 Durian. In Tropical and subtropical fruits (ed. P. E. Shaw, Jr., H. T Chan. & S. Nagy), pp. 261-289. AgScience, Auburndale, Florida.

Ibarra-Perez F. J., Barnhart D., Ehdaie B., Knio K. M., Waines J. G. 1999 Effects of insect tripping on seed yield of common beans. Crop Sci. 39, 428-433. I

sh- Am, G. & Eisikowitch, D. 1993 The behaviour of honey bees (Apis mellifera) visiting avocado (Persea americana) flowers and their contribution to its pollination. J. Apic. Res. 32, 175-186.

Ish- Am, G. & Eisikowitch, D. 1998a Low attractiveness of avocado (Persea americana Mill.) flowers to honeybees (Apis mellifera L.) limits fruit set in Israel. J. Hort. Sci. Biotech. 73, 195-204.

Ish- Am, G. & Eisikowitch, D. 1998b Mobility of honey bees during foraging in avocado orchards. Apidologie 29, 209-219.

Ish-Am, G., Barrientos-Priego, Fl, Castañeda-Vildozola, A. & Gazit, S. 1999 Avocado (Persea americana Mill) pollinators in its region of origin, Rev. Chapingo Serie Hortic. 5, 137-143.

Ivancic, A., Lebot, V., Roupsard, O., Garcia, J.Q. & Okpui, T. 2004 Thermogenic flowering of taro (Colocasia esculenta, Araceae). Can. J. Bot. 82, 1557-1565.

Jacob, V. J. 1980 Pollination, fruit setting and incompatibility in Cola nitida. Incompatibility Newslett. 12, 50-56.

James D., Ariyanayagam R. P. & Duncan E. J. 1989 Effects of tripping flowers of four early varieties of pigeonpea (Cajanus cajan L. Millsp.). J. Hort. Sci. 64, 243-247.

James, N. I. 1980 Sugarcane. In Hybridization of crop plants (ed. W. R. Fehr & H. H. Hadley) pp. 617- 629. American Society of Agronomy & Crop Science Society fo America, Madison, WI, USA.

Jarlan, A., De Oliveira, D. & Gingras, J. 1997a Pollination by Eristalis tenax (Diptera: syrphidae) and seed set of greenhouse sweet pepper. J. Econ. Entomol. 90, 1646-1649.

Jarlan, A., De Oliveira, D. & Gingras, J. 1997b Effects of Eristalis tenax (Diptera: syrphidae) pollination on characteristics of greenhouse sweet pepper fruits J. Econ. Entomol. 90, 1650-1654.

Javorek, S. K., Mackenzie, K. E. & Vander Kloet, S. P 2002 Comparative pollination effectiveness among bees (Hymenoptera: Apoidea) at lowbush blueberry (Ericaceae: Vaccinium angustifolium Ait.). Ann. Entomol. Soc. Am. 95, 345-351.

Jicinska, D. 1976 Autogamy in various species of the genus Rosa. Preslia 48, 225-229.

Jindal, K. K. & Sharma, G. 1997 Tropical fruits - mango, banana, papaya. In Fruit crop pollination (ed. L. R. Verma & K. K. Jindal), pp 279-297. Kalyani Pub., Ludhiana, India.

Jones, A. 1980. Sweet potato. In Hybridization of crop plants (ed. W.R. Fehr & H. H. Hadley) pp. 645-655. American Society of Agronomy & Crop Science Society of America, Madison, WI, USA.

Kakutani, T., Inoue, T., Tezuka, T. & Maeta, Y. 1993 Pollination of strawberry by the stingless bee, Trigona minangkabau, and the honey bee, Apis mellifera: an experimental study of fertilization efficiency. Res. Popul. Ecol. 35, 95-111.

Kamenetsky, R. & Rabinowitch, H. D. 2001 Floral development in bolting garlic. Sex Plant Reprod. 13, 235- 241.

Kanchanapoom, K. & Kanchanapoom, M. 1998. Mangosteen. In Tropical and subtropical fruits (ed. P. E. Shaw, Jr. H. T Chan. & S. Nagy) pp. 191-215. AGSCIENCE, INC., Auburndale, Florida, USA.

Kato, E. C. & Nogueira-Couto, R. H. 2002 Polinização em melão (Cucumis melo L.) dos tipos amarelo e rendilhado. Naturalia 27, 201-210.

Kaul, P., Sharma, N. & Koul, A. K. 1993 Pollination biology of Apiaceae. Curr. Sci. 65, 219-222.

Kawano, K. 1980. Cassava. In Hybridization of crop plants (ed. W. R. Fehr & H. H. Hadley) pp. 225-233. American Society of Agronomy & Crop Science Society of America, Madison, WI, USA.

Kausch-Blecken von Schmeling, W. 1992 Der Speierling (Sorbus domestica L.) Arterhaltung durch Nachzucht, Goltze Druck GmbH & Co., Göttingen Germany, p. 219.

Kelly, B. A, Hardy, O. J, & Bouvet, J. M. 2004 Temporal and spatial genetic structure in Vitellaria paradoxa (shea tree) in an agroforestry system in southern Mali. Mol. Ecol. 13, 1231-1240.

Kempler, C. & Kabaluk, T. 1996 Babaco (Carica pentagona Heilb.): A possible crop for the greenhouse. HortScience 31, 785-788.

Kevan, P. G., Eisikowitch, D., Ambrose, J. D. & Demp, J. R. 1990 Cryptic dioecy and insect pollintion in Rosa setigera Michx. (Rosaceae), a rare plant of Carolinian Canada. Biol. J. Linn. Soc. 40, 229-243.

Kevan, P. G. 2003 Pollination. In Encyclopedia of rose science. Vol. 2. (ed. A. V. Roberts, T. Debener & S. Gudin), pp. 456-460. Elsevier Academic Press, Oxford, United Kingdom.

Khan B. M., Shahid M., Chaudhry M. I. 1986 Effect of honey bee pollination on the fruit setting and yield of loquat. Pakistan J. For. 36, 73-77.

Kill, L. H. P. & da Costa, J. G. 2003 Floral biology and reproductive system of Annona squamosa L. (Annonaceae) in Petrolina-PE, Brazil. Cienc. Rural 33, 851-856.

Klein, A.-M., Steffan- Dewenter, I. & Tscharntke, T. 2003a Bee pollination and fruit set of Coffea arabica and C. canephora (Rubicacea). Am. J. Bot. 90, 153-157.

Klein, A.-M., Steffan- Dewenter, I. & Tscharntke, T. 2003b Pollination of Coffea canephora in relation to local and regional agroforestry management. J. Appl. Ecol. 40, 837-845. (doi:10.1046/j.1365- 2664.2003.00847.x)

Klein, A.-M., Steffan- Dewenter, I. & Tscharntke, T. 2003c Fruit set of highland coffee increases with the diversity of pollinating bees. P Proc. R. Soc. London B 270, 955-961. (doi:10.1098/rspb.2002.2306)

Knuth, P. 1908 Handbook of flower pollination, Vaolume II, Clarendon Press, Oxford.

Koelling, P. D., Kenworthy, W. J. & Caron, D. M. 1981 Pollination of male-sterile soybeans, Glycine max, in caged plots, Crop Sci. 21, 559-561.

Koltowski, Z., Jablonski, B., Szklanowska, K. & Pluta, S. 1997 The influence of pollinating insects on the yield of more important new black currant cultivars (Ribes nigrum L.) Pszczeln. Zesz. Nauk. 41, 19-31.

Koltowski, Z., Pluta, S., Jablonski, B. & Szklanowska, K. 1999 Pollination requirements of eight cutivars of black currant (Ribes nigrum L.). J. Hort. Sci. Biotech. 74, 472- 474.

Koul, P., Sharma, N. & Koul A. K. 1993 Pollination biology of the Apiaceae. Curr. Sci. 65, 219-222.

Krantz, G. W. & Poinar, G. O. 2004 Mites, nematodes and the multimillion dollar weevil. J. Nat. Hist. 38, 135- 141.

Kremen, C., Williams, N. M. & Thorp, R. W. 2002 Crop pollinaton from native bees at risk from agricultural intensification. Proc. Nat. Acad. Sci. USA 99, 16812-16816.

Kremen, C., Williams, N. M., Bugg, R. L., Fay, J. P. & Thorp, R. W. 2004 The area requirements of an ecosystem service: crop pollination by native bee communities in California. Ecol. Lett. 7, 1109-1119.

Kron, P., Husband, B. C., Kevan, P. G. & Belaoussoff, S. 2001 Factors affecting pollen dispersal in highdensity apple orchards. Hortscience 36, 1039-1046.

Lachenaud, P. 1994 Variations in the number of beans per pods in Theobroma cacao L. in the Ivory Coast. 1. the role of pollen. J. Hort. Sci. 69, 1123-1129.

Ladizinsky G., Braun D., Goshen D. & Muehlbauer F. J. 1984 The biological species of the genus Lens L. Bot. Gazette 145, 253-261.

Ladurner, E., Recla, L., Wolf, M., Zelger, R. & Burgio, G. 2004 Osmia cornuta (Hymenoptera Megachilidae) densities required for apple pollination: a cage study. J. Apicult. Res. 43, 118-122.

Lakshmi, K. & Mohana Rao, G. 1998 Plants for bees – Guava. Bee World 79, 135-137.

Langenberger, M. W. & Davis, A. R. 2002 Temporal changes in floral nectar production, reabsorption, and composition associated with dichogamy in annual caraway (Carum carvi; Apiaceae). Am. J. Bot. 89, 1588-1598.

Larter, E. N. & Gustafson, J. P. 1980 Triticale. In Hybridization of crop plants (ed. W. R. Fehr & H. H. Hadley) pp. 681-694. American Society of Agronomy & Crop Science Society of America, Madison, WI, USA.

Le Guen, J., Mesquida, J., Pierre, J. S., Morin, G., Taséi, J. N. & Carré, S. 1993 Efficacité pollinisatrice de différents traitements sur 2 lignées de féverole de printemps (Vicia faba L. var. equina Steudel), à des niveaux d'autofertilité différents, avec utilisation de diverses espèces de Bombus Latr. (Hymenoptera: Apidae). Apidologie 24, 129-145.

Lim, T. K & Luders, L. 1998 Durian flowering, pollination and incompatibility studies. Ann. Appl. Biol. 132, 151- 165.

Liu, X.H. & Ma, C.L. 2001 Production and research of longan in China. Acta Hort. 558, 73-82.

Lughada, E. N. & Proenca, C. 1996 A survey of the reproductive biology of the Myrtoideae (Myrtaceae). Ann. Missouri Bot. Gard. 83, 480-503.

Lumkin, D. 2005 Overcoming obstacles during 2005 almond pollination. Am. Bee J. 145, 290-294.

Maccagnani, B., Ladurner, E., Santi, F. & Burgio, G. 2003 Osmia cornuta (Hymenoptera, Megachilidae) as a pollinator of pear (Pyrus communis): fruit- and seed-set. Apidologie 34, 207-216. (doi:10.1051/apido:2003009)

Maeta, Y., Tezuka, T., Nadano, H. & Suzuki, K. 1992 Utilization of the Brazilian stingless bee, Nannotrigona testaceicornis, as a pollinator of strawberries. Honeybee Sci. 13, 71-78.

Malagodi-Braga, K. S. & Kleinert, A. M. P. 2004 Could Tetragonisca angustula Latreille (Apinae, Meliponini) be effective as strawberry pollinator in greenhouses? Aust. J. Agr. Res. 55, 771-773.

Malerbo-Souza, D. T. & Nogueira-Couto, R. H. 1997 Atrativo para as abelhas Apis mellifera e polinização em café (Coffea arabica L.) Mensagem Doce 44, 6-11.

Manino, A., Patetta, A. & Marletto, F. 1991 Investigations on chesnut pollination. Acta Hort. 288, 335-339.

Manning, R. & Boland, J. 2000 A preliminary investigation into honey bee (Apis mellifera) pollination of canola (Brassica napus cv. Karoo) in Western Australia. Aust. J. Exp. Agr. 40, 439-442.

Manrique, A. J. & Thimann, R. E. 2002 Coffee pollination with africanized honeybees in Venezuela. Interciencia 27, 414- 416.

Maroto, J. V., Miguel, A., Lopez-Galarza, S., San Bautista, A., Pascula, B., Alagarda, J. & Guardiola, J. L. 2005 Parthenocarpic fruit set in triploid watermelon induced by CPPU and 2,4-D applications. Plant Growth Reg. 45, 209-213.

Mars, M. & Marrakchi, M. 2004 Dynamique de floraison et régime de reproduction chez le grenadier (Punica granatum L.) en Tunisie. Fruits 59, 39-48. (doi:10.1051/fruits :2004005)

Maués, M. M. 2002 Reproductive phenology and pollination of the Brazil nut tree (Bertholletia excelsa Humb. & Bonpl. Lecythidaceae) in Eastern Amazonia. In Pollinating bees - the conservation link between agriculture and nature. (ed. P. Kevan & V. L. Imperatriz Fonseca) pp. 245-254. Ministry of Environment, Brasilia, Brazil.

Mayfield, M. M. 2005 The importance of nearby forest to known and potential pollinators of oil palm (Elaeis guineensis Jacq.; Arecaceae) in Southern Costa Rica. Econ. Bot. 59, 190–196.

McGregor, S. E. 1976 Insect pollination of cultivated crop plants. Washington (DC). US Departement of Agriculture, Agricultural Handbook 496. <http://gears.tucson.ars.ag.gov/book/index.html>

McLaren, G. F., Fraser, J. A., Grant, J. E. 1995 Pollination compatibility of apricots grown in central Otago, New Zealand. Acta Hort. 384, 385-390.

Mcphee K. E. 2003 Dry pea production and breeding - a mini-review. Food Agric. Environ. 1, 64-69.

Mehta, K. & Kashyap, A. S. 1997 Persimmon. In Fruit crop pollination. (ed. Verma, L. R. & Jindal, K. K.) pp. 205-211. Kalyani Pub., Ludhiana, Indiah.

Meisels, S. & Chiasson, H. 1997 Effectiveness of Bombus impatiens Cr. as pollinators of greenhouse sweet peppers (Capsicum annuum). Acta Hort. 437, 425-429.

Meléndez-Ramírez, V., Parra-Tabla, V., Kevan, P. G., Ramírez-Morillo, I., Harries, H., Fernández-Barrera, M. & Zizumbo-Villareal, D. 2004 Mixed mating strategies and pollination by insects and wind in coconut palm (Cocos nucifera L. (Arecaceae)): importance in production and selection. Agr. Forest Entomol. 6, 155-163.

Melgarejo, P., Legua, P., Martinez, M. & Martinez, J. J. 2000 Contribution to a better knowledge of the quality of pomegranante pollen (Punica granatum L.) Options Méditerr. A. 42, 115-121.

Mickelbart, M.V. 1996 Sapodilla: A potential crop for subtropical climates. In Progress in new crops. (ed. J. Janick). pp 439-446. ASHS Press, Alexandria, VA.

Millogo-Rasolodimby, J. 1989 Importance apicole du karité, Butyrospermum paradoxum (Gaertn. Hepper) et du néré, Parkia biglobosa (Jacq. Benth.) Rev. Française d'Apiculture 482, 72-74.

Miura, T. 1982 The hourly change of the pollinator association found in the Japanese persimmon, Diospyros kaki cultivar Saijo orchard during daytime. Bulletin of the Faculty of Agriculture, Shimane University, 166- 171.

Moncur, M. W. 1985 Floral ontogeny of the jackfruit, Artocarpus heterophyllus. Aust. J. Bot. 33, 585-593.

Monzón, V. H., Bosch, J. & Retana, J. 2004 Foraging behaviour and pollinating effectiveness of Osmia cornuta (Hymenoptera: Megachilidae) and Apis mellifera (Hymenoptera: Apidae) on “Comice” pear. Apidologie 35, 575- 585. (doi:10.1051/apido:2004055)

Morandin, L. A., Laverty, T. M. & Kevan, P. G. 2001 Bumble bee (Hymenoptera: Apidae) activity and pollination levels in commercial tomato greenhouses. J. Econ. Entomol. 94, 462-467.

Morandin, L. A. & Winston, M. L. 2005 Wild bee abundance and seed production in conventional organic, and genetically modified canola. Ecol. Appl. 15, 871-881.

Moreti, A. C. de C. C., da Silva, E. C. A. & Alves, M. L. T. M. F. et al. 1998 Observações sobre a poinização entomófila da cultura da soja (Glycine max Merril). Boletim da indústria Animal 55, 91-94.

Moreti, A. C. de C. C., da Silva, R. M. B., da Silva, E. C. A. et al. 1996 Aumento na produção de sementes de girassol (Helianthus annuus) Pela ação de insetos polinizadores. Sci. Agric. 53, 280-284.

Morey, D. D. & Barnett, R. D. 1980 Rye. In Hybridization of crop plants (ed. W. R. Fehr & H. H. Hadley) pp. 523-534. American Society of Agronomy & Crop Science Society of America, Madison, WI, USA.

Mori, S. A. & Prance, G. T. 1990 Taxonomy, ecology, and economic botany of the Brazil nut (Bertholletia excelsa Humb. & Bonpl.: Lecythidaceae). Adv. Econ. Bot. 8, 130-150.

Morton, J. F. 1987 Fruits of warm climates. Creative Resource Systems, Inc., Winterville, NC. Mutsaers, M. 1993 Natural pollination of banana and plantain at Onne. MusAfrica 2, 2-3.

Nadel, H. & Pena, J. E. 1994 Identity, behaviour, and efficacy of nitidulid beetles (Coleoptera: Nitidulidae) pollinating commercial Annona species in Florida. Environ. Entomol. 23, 878-886.

Nadgauda, R. S., John, C. K., Parasharami, V. A., Joshi, M. S. & Mascarenhas A. F. 1997 A comparison of in vitro with in vivo flowering in bamboo: Bambusa arundinacea. Plant Cell Tissue Organ Culture 48, 181- 188.

Németh, E., Bernath, J. & Petheo, F. 1999 Study on flowering dynamic and fertilization properties of caraway and fennel. Acta Hort. 502, 77-83.

Németh, E. & Székely, G. 2000 Floral biology of medicinal plants. I. Apiaceae species. International Journal of Horticultural Science 6, 133-136.

Nepi, M. & Paccini, E. 1993 Pollination, pollen viability, and pistil receptivity in Curcurbita pepo. Ann. Bot. 72, 527-536.

Njoroge, G. N., Gemmill, B., Bussmann, R., Newton, L. E. & Ngumi, V. W. 2004 Pollination ecology of Citrullus lanatus at Yatta, Kenya. Int. J. Trop. Insect Sci. 24, 73-77.

Nogueira-Couto, R. H., Pereira, J. M. S & De Jong, D. 1998 Pollination of Glycine wightii, a perennial soybean, by Africanized honey bees. J. Apicult. Res. 3, 289-291.

Norden, B. M. B. 1985 The comparative importance of small carpenter bees (Ceratina spp.) and other insects to pollination of muskmelon/cantaloupe (Cucumis melo L.) in Maryland. Ph.D. dissert., Univ. Maryland.

Nyéki, J. & Soltész, M. 2003 Pear (Pyrus communis L.). In Floral biology, pollination and fertilisation in temperate zone fruit species and grape (ed. P. Kozma, J. Nyéki, M. Soltész & Z. Szabó) pp. 317-331. Akadémiai Kiadó, Budapest, Hungary.

Nyéki, J., Szabó, Z. & Soltész, M. 2003a Sweet cherries (Prunus avium L.). In Floral biology, pollination and fertilisation in temperate zone fruit species and grape (ed. P. Kozma, J. Nyéki, M. Soltész & Z. Szabó) pp. 341-358. Akadémiai Kiadó, Budapest, Hungary.

Nyéki, J., Szabó, Z. & Soltész, M. 2003b Sour cherries (Prunus cerasus L.). In Floral biology, pollination and fertilisation in temperate zone fruit species and grape (ed. P. Kozma, J. Nyéki, M. Soltész & Z. Szabó ) pp. 359-382.Akadémiai Kiadó, Budapest, Hungary.

O’Malley, D. M., Buckley, D. P., Prance, G. T. & Bawa, K. W. 1988 Genetics of Brazil nut (Bertholletia excelsa Humb. & Bonpl.: Lecythidacea). 2. Mating system. Theoretical and Applied Genetics 76, 929-932.

Osei, J. K. 1995-1996 Fruit setting from natural pollination and the yield of nine-year-old cross-compatible Cola nitida clones. Ghana J. Agric. Sci. 28-29, 95-98

Patterson, K. J. 1990 Effects of pollination on fruit set, size, and quality in feijoa. New Zeal. J. Crop Hort. 18, 127-131.

Payne, J. A., Cane, J. H., Amis, A. A. & Lyrene, P. M. 1989 Fruit size, seed size, seed viability and pollination of rabbiteye blueberries (Vaccinium ashei Reade). Acta Hort. 241:38-43.

Pelletier, L., Brown, A., Otrysko, B. & McNeil, J. N. 2001 Entomophily of the cloudberry (Rubus chamaemorus). Entomol. Expt. Appl. 101, 219-224.

Peña, J. E., Castiñeiras, A., Bartelt, R. & Duncan, R. 1999 Effect of pheromone bait stations for sap beetles (Coleoptera: Nitidulidae) on Annona spp. fruit set. Florida Entomol. 82, 475-480.

Petanidou, T. & Vokou, D. 1990 Pollination and pollen energetics in Mediterranean ecosystems. Am. J. Bot. 77, 986-992.

Phipps, J. 2003 Hawthorns and medlars. 180 pp. Timber Press, Portland, OR, USA.

Pías, B. & Guitián, P. 2006 Breeding system and pollen limitation in the masting tree Sorbus aucuparia L. (Rosaceae) in the NW Iberian Peninsula. Acta Oecol. 29, 97-103.

Piatos, P. & Knight, R. J. 1975 Self-incompatibility in the sapodilla. Proc. Florida State Hort. Soc. 88, 464-465.

Pierre, J., Suso, M. J., Moreno, M. T., Esnault, R. & Le Guen, J. 1999 Diversité et efficacité de l'entomofaune pollinisatrice (Hymenoptera: Apidae) de la féverole (Vicia faba L.) sur 2 sites, en France et en Espagne. Ann. Soc. Entomol. Fr. 35 (suppl.), 312-318.

Pinzauti, M., Lazzarini, D. & Felicioli, A. 1997 Preliminary investigation of Osmia cornuta Latr. (Hymenoptera, Megachilidae) as a potential pollinator for blackberry (Rubus fruticosus L.) under confined environment. Acta Hort. 427, 329-333.

Plaisted, R. L. 1980 Potato. In Hybridization of crop plants (ed. W. R. Fehr & H. H. Hadley) pp. 483-494. American Society of Agronomy & Crop Science Society of America, Madison, WI, USA.

Pomper, K .W., Layne, D. R., Peterson, R. N. & Wolfe, D. 2003 The pawpaw regional variety trial: background and early data. HortTechnology 13, 412-417.

Pouvreau, A. 1984 Production de semences potagères In Pollinisation et productions végétales (ed. P. Pesson & J. Louveaux) pp 471-495. INRA, Paris, France.

Raccuia, S. A., Mainolfi, A., Mandolino, G. & Melilli, M. G. 2004 Genetic diversity in Cynara cardunculus revealed by AFLP markers: comparsion between cultivars and wild types from Sicily. Plant Breed. 123, 280-284.

Rana, H. S. & Divedi, M. P. 1997 Pomegranate. In Fruit crop pollination (ed. L. R Verma & K. K. Jindal) pp. 331-344. Kalyani Pub., Ludhiana, India.

Ragone, D. 1997 Breadfruit Artocarpus altilis (Parkinson) Fosberg. Promoting the conservation and use of underutilized and neglected crops. 10. IPK, Gatersleben, Germany / IPGRI, Rome, Italy. 77 p.

Ramirez, N. 2004 Ecology of pollination in a tropical Venezuelan savanna. Plant Ecol. 173, 171-189.

Rasmont, P., Regali, A., Ings, T. C., Lognay, G., Baudart, E., Marlier, M., Delcarte, E., Viville, P., Marot, C., Falmagne, P., Verhaeghe, J. C. & Chittka, L. 2005 Analysis of pollen and nectar of Arbutus unedo as a food source for Bombus terrestris (Hymenoptera: Apidae). J. Econ. Entomol. 98, 656-663.

Raspé, O. 1998 Biologie de la reproduction et variation genetique d'un arbre entomophile: Sorbus aucuparia L. (Rosaceae: Maloideae). PhD Thesis, Universite catho- lique de Louvain, Belgium

Raw, A. 2000 Foraging behavior of wild bees at hot pepper flowers (Capsicum annuum) and its possible influence on cross pollination. Ann. Bot. 85, 487- 492.

Ray, J. D., Kilen, T. C., Abel, C. A. & Paris, R. L. 2003 Soybean natural cross-pollination rates under field conditions. Environ. Biosafety Res. 2,133-138.

Reddi, E .U. B. 1989 Thrips pollination in sapodilla (Manilkara zapota). Proc. Indian Nat. Sci. Acad. B 55, 407- 410.

Reiter, R. 1947 The coloration of anther and corbicular pollen. Ohio J. Sci. 47, 137-152. Rhodes, J. 2002 Cotton pollination by honey bees. Aust. J. Exp. Agric. 42, 513-518.

Ricciardelli D’Albore, G. C. 1986 The pollinating insects of some Umbelliferae of agricultural and herbal interest (Angelica archangelica L., Carum carvi L., Petroselinum crispum A.W. Hill, Apium graveolens L., Pimpinella anisum L., Daucus carota L., Foeniculum vulgare Miller V. azoricum Thell.) Apidologie 17, 107-124.

Richards, A. J. 2001 Does low biodiversity resulting from modern agricultural practice affect crop pollination and yield? Ann. Bot. 88, 165-172.

Richards, A. J. 1990 Studies in Garcinia, dioecious tropical forest trees: the origin of the Mangosteen. Bot. J. Linn. Soc. 103, 301-308.

Ricketts, T. H. 2004 Tropical forest fragments enhance pollinator activity in nearby coffee crops. Conserv. Biol. 18, 1262-1271.

Ricketts, T. H., Daily, G. C., Ehrlich, P. R. & Michener, C. D. 2004 Economic value of tropical forest to coffee production. Proc. Nat. Acad. Sci. USA 101, 12579-12582.

Robertson, C. 1923 Flowers and insects XXII. Bot. Gaz. 75, 60-74. Rohrer, J. R., Robertson, K. R., Phipps, J. B. 1994 Floral morphology of Maloideae (Rosaceae) and its systematic relevance. Am. J. Bot. 81, 574-581.

Ronnie Coffman, W. & Herrera, R. M. 1980 Rice. In Hybridization of crop plants (ed. W. R. Fehr & H. H. Hadley) pp. 511-522. American Society of Agronomy & Crop Science Society of America, Madison, WI, USA.

Roubik, D. W. 1995 Pollination of cultivated plants in the tropics. Food and Agriculture Organization of the United Nations, Rome. Bull. 118. Italy.

Roubik, D. W. 2002a Feral African bees augment neotropical coffee yield. In The conservation link between agriculture and nature (ed. P. G. Kevan & Imperatriz-Fonseca, V. L.), pp. 255-266. Ministry of Environment: Secretariat for Biodiversity and Forests, Brazil.

Roubik, D. W. 2002b The value of bees to the coffee harvest. Nature 417, 708.

Russell, W. A. & Hallauer, A. R. 1980 Corn. In Hybridization of crop plants (ed. W. R. Fehr & H. H. Hadley) pp. 299-312. American Society of Agronomy & Crop Science Society of America, Madison, WI, USA.

Routley, M. B., Kron, P. & Husband, B. C. 2004 The consequences of clone size for paternal and maternal success in domestic apple (Malus domestica). Am. J. Bot. 91, 1326-1332.

Sakai, S., Kato, M. & Nagamasu, H. 2000 Artocarpus (Moraceae) - Gall midge pollination mutualism mediated by a male-flower parasitic fungus. Am. J. Bot. 87, 440-445

Salakpetch, S., Chandraparnik, S. & Hiranpradit, H. 1992 Pollen grains and pollination in Durian, Durio zibethinus Murr. Acta Hort. 321, 636-640.

Sampson, B. J. & Cane J. H. 2000 Pollination efficiencies of three bee (Hymenoptera: Apoidea) species visiting rabbiteye blueberry. J. Econ. Entomol. 93, 1726-1731.

Sampson, B. J., Danka, R. G. & Stringer, S. J. 2004 Nectar robbery by bees Xylocopa virginica and Apis mellifera contributes to the pollination of rabbiteye blueberry. J. Econ. Entomol. 97, 735-740.

Sampson, B., Noffsinger, S., Gupton, C. & Magee, J. 2001 Pollination biology of the muscadine grape. Hortscience 36, 120-124.

Sampson, B. J., Stringer, S. J., Cane, J. H. & Spiers, J. M. 2004 Screenhouse evaluations of a mason bee Osmia ribifloris (Hymenoptera: Megachilidae) as a pollinator of blueberries in the southeastern United States. Small Fruits Review 3, 381-392.

Sanford, M. T. 2003 Pollination of citrus by honey bees. FRAA092, Florida Cooperative Extension Service. Instiute of Food and Agricultural Sciences, University of Florida, Florida. available at <http://edis.ifas.ufl.edu/pdffiles/AA/AA09200.pdf>

Sanou H., Lovett P. N. & Bouvet J. M. 2005 Comparison of quantitative and molecular variation in agroforestry populations of the shea tree (Vitellaria paradoxa C.F. Gaertn) in Mali. Mol. Ecol. 14, 2601-2610.

Sargent, R. D. & Otto, S. P. 2004 A phylogenetic analysis of pollination mode and the evolution of dichogamy in angiosperms. Evol. Ecol. 6,1183-1199.

Sasikumar, B., Krishnamoorthy, B., Saji, D. V., Johnson, K. G, Peter, K. V. & Ravindran, P. N. 1999 Spice diversity and conservation of plants that yield major spices in India. Plant Genetic Resources Newsletter 118, 19-26.

Schertz, K. F. & Dalton, L. G. 1980 Sorghum. In Hybridization of crop plants (ed. W. R. Fehr & H. H. Hadley) pp. 577-588. American Society of Agronomy & Crop Science Society of America, Madison, WI, USA.

Schittenhelm, S., Gladis, T. & Rao, V. R. 1997 Efficiency of various insects in germplasm regeneration of carrot, onion and turnip rape accessions. Plant Breed. 116, 369-375.

Schroeder, C. A. 1953 The pollination of some subtropical fruit trees. Lasca Leaves 3, 39-41. Sealy, J. R. 1949 Arbutus unedo. J. Ecol. 37, 365-388.

Segnou, C. A., Fatokun, C. A., Akoroda, M. O. & Hahn, S. K. 1992 Studies on the reproductive biology of white yam (Dioscorea rotundata Poir). Euphytica 64, 197-203.

Sekita, N. 2001 Managing Osmia cornifrons to pollinate apples in Aomori Perfecture, Japan. Acta Hort. 561, 303-307.

Sekita, N. & Amada, Y. 1993 Use of Osmia cornifrons to pollinate apples in Aomori Prefecture, Japan. Japan Agricultural Research Quarterly 26, 264-270.

Sharma, H. K., Gupta, J. K. & Thakur, J. R. 2004 Effect of bee pollination and polliniser proportion on apple productivity. Acta Hort. 662, 451-454.

Sharma, G. & Jindal, K. K. 1997 Sub-tropical fruits - Citrus, Litchi, Ber, Guava, Sapota, Jamun, Loquat, Aonla, Fig. In Fruit crop pollination (ed. L. R.Verma & K. K. Jindal), pp. 298-330. Kalyani Pub., Ludhiana, India.

Shipp, J. L., Whitfield, G. H., Papadopoulos, A. P. 1994 Effectiveness of the bumble bee Bombus impatiens Cr. (Hymenoptera: Apidae), as a pollinator of greenhouse sweet pepper. Sci. Hortic. 57, 29-39.

Simmonds, N. W. 1965 The grain chenopods of the tropical American highlands. Econ. Bot. 19, 223-235.

Simmonds, N. W. 1971 The breeding system of Chenopodium quinoa. Male sterility. Heredity 27, 73-82.

Singh, R.P. 1997 Olive In Fruit crop pollination (ed. L. R.Verma & K. K. Jindal), pp. 212-224. Kalyani Pub., Ludhiana, India.

Slaa, E. J., Sánchez, C. L. A., Malagodi-Braga, K. S. & Hofstede, F. E. 2006 Stingless bees in applied pollination. Practice and perspectives. Apidologie 37, 293-315. (doi:10.1051/apido:2006022)

Smith, G. A. 1980 Sugarbeet. In Hybridization of crop plants (ed. W.R. Fehr & H. H. Hadley) pp. 601-616. American Society of Agronomy & Crop Science Society of America, Madison, WI, USA.

Soltész, M. 2003 Apple (Malus sylvestris (L.) Mill). In Floral biology, pollination and fertilisation in temperate zone fruit species and grape (ed. P. Kozma, J. Nyéki, M. Soltész & Z. Szabó) pp 237-316. Akadémiai Kiadó, Budapest, Hungary.

Soltész, M., Nyéki, J. & Szabó Z. 2003a Almond (Amygdalus communis L.). In Floral biology, pollination and fertilisation in temperate zone fruit species and grape (ed. P. Kozma, J. Nyéki, M. Soltész & Z. Szabó) pp. 435-449. Akadémiai Kiadó, Budapest, Hungary.

Soltész, M., Nyéki, J. & Szabó, Z. 2003b Ribes species. In Floral biology, pollination and fertilisation in temperate zone fruit species and grape (ed. P. Kozma, J. Nyéki, M., Soltész & Z. Szabó) pp. 499-513. Akadémiai Kiadó, Budapest, Hungary.

Somerville, D. C. 1999. Honeybees (Apis mellifera L.) increase yields of faba beans (Vicia faba L.) in New South Wales while maintaining adequate protein requirements from faba bean pollen. Austral. J. Exp. Agric. 39,1001-1005.

Spalik, K. 1996 Species boundaries, phylogenetic relationships, and ecological differentation in Anthriscus (Apiaceae). Plant Syst. Evol. 199, 17-32.

Sperens, U. 1996 Is fruit and seed production in Sorbus aucuparia L. (Rosaceae) pollen-limited? Ecoscience 3, 325-329.

Stanghellini, M. S., Ambrose, J. T. & Schultheis, J. R. 1998 Seed production in watermelon: a comparison between two commercially available pollinators. HortScience 33, 28-30.

Stanghellini, M. S., Ambrose, J. T. & Schultheis, J. R. 1997 The effects of honey bee and bumble bee pollination on fruit set and abortion of cucumber and watermelon. Am. Bee J. 137, 386-391.

Stanghellini, M. S., Ambrose, J. T. & Schultheis, J. R. 2002 Diurnal activity, floral visitation and pollen deposition by honey bees and bumble bees on field-grown cucumber and watermelon. J. Apic. Res. 41, 27-34.

Starling, T. M. 1980 Barley. In Hybridization of crop plants (ed. W. R. Fehr & H. H. Hadley) pp. 189-202. American Society of Agronomy & Crop Science Society of America, Madison, WI, USA.

Stern, R. A., Eisikowitch, D. & Dag, A. 2001 Sequential introduction of honeybee colonies and doubling their density increases cross-pollination, fruit-set and yield in 'Red Delicious' apple J. Hortic. Sci. Biotech. 76, 17-23.

Stern, R. A. & Gazit, S. 1996 Lychee pollination by the honeybee. J. Am. Soc. Hort. Sci. 120, 152-157

Stern, R. A., Goldway, M., Zisovich, A. H., Shafir, S. & Dag, A. 2004 Sequential introduction of honeybee colonies increases cross-pollination, fruit-set and yield of ‘Spadona’ pear (Pyrus communis L.). J. Hortic. Sci. Biotech. 79, 652- 658.

Stewart, A. M. 1989 Factors affecting pollinator effectiveness in Feijoa sellowiana. New Zeal. J. Crop Hort. 17, 145-154.

Stewart, A. M. 1984 Studies on the pollination biology of an introduced crop Feijoa sellowiana. New. Zeal. J. Ecol. 7, 203-204.

Stougaard, B. 1983 Pollination in Rosa multiflora. Tisskrift for planteavl. 87, 633-642.

Stubbs, C. S. & Drummond, F. A. 2001 Bombus impatiens (Hymenoptera: Apidae): An alternative to Apis mellifera (Hymenoptera: Apidae) for lowbush blueberry pollination. J. Econ. Entomol. 94, 609-616.

Stubbs, C. S. & Drummond, F. A. 1999 Pollination of lowbush blueberry by Anthophora pilipes villosula and Bombus impatiens (Hymenoptera: Anthophoridae and Apidae). J. Kan. Entomol. Soc. 72, 330-333.

Suso, M. J., Moreno, M. T., Mondragao-Rodrigues, F. & Cubero, J. I. 1996 Reproductive biology of Vicia faba: role of pollination conditions. Field Crops Res. 46, 81-91.

Szabó, Z. 2003. Plum (Prunus domestica L.) 2003 In Floral biology, pollination and fertilisation in temperate zone fruit species and grape (ed. P. Kozma, J. Nyéki, M., Soltész & Z. Szabó) pp. 383-410. Akadémiai Kiadó, Budapest, Hungary.

Szabó, Z., Nyéki, J. & Soltész, M. 2003a Apricot (Prunus armeniaca L.). In Floral biology, pollination and fertilisation in temperate zone fruit species and grape (ed. P. Kozma, J. Nyéki, M., Soltész & Z. Szabó) pp. 411-423. Akadémiai Kiadó, Budapest, Hungary.

Szabó, Z., Nyéki, J. & Soltész, M. 2003b Peaches (Prunus persica (L.) Batsch). In Floral biology, pollination and fertilisation in temperate zone fruit species and grape (ed. P. Kozma, J. Nyéki, M., Soltész & Z. Szabó) pp. 425-434. Akadémiai Kiadó, Budapest, Hungary.

Tandon, R., Manohara, T. N., Nijalingappa, B. H. M. & Shivana, K. R. 2001 Pollination and pollen-pistil interaction in oil palm, Elaeis guineensis. Ann. Bot. 87, 831-838.

Tayyar, R. I., Nguyen, J. H. T. & Holt, J. S. 2003 Genetic and morphological analysis of two novel nutsedge biotypes from California. Weed Sci. 51, 731-739.

Tchuenguem Fohouo, F. N., Mapongmetsem, P. M., Hentchoya Hemo, J. & Messi, J. 2005 Exploitation des fleurs de quatre plantes oleagineused par Apis mellifera angaundere (Cameroun): Bombax pentandrum, Vitellaria paradoxa, Lophira lanceolata et Dacryodes edulis. Procédés Biologiques et Alimentaires: <http://spip.cm.refer.org/pba1/article51.html>

Thakur, B. S. & Thakur K. S. 1997 Grapes. In Fruit crop pollination (eds. L.R. Verma LR & K.K. Jindal) pp. 241-249. Kalyani Pub., Ludhiana, India.

Thien, L. B., White, D. A. & Yatsu. L. Y. 1983 The reproductive biology of a relict – Illicium floridanum Ellis. Am. J. Bot. 70, 719-727.

Thomson, J. D. & Goodell, K. 2001 Pollen removal and deposition by honeybee and bumblebee visitors to apple and almond flowers. J. Appl. Ecol. 38, 1032-1044.

Torre Grossa, J. P., Vaissière, B. E, Rodet, G., Botella, L. & Cousin, M. 1994 Pollination needs of the selfcompatible almond cultivar ‘Lauranne’ Acta Hort. 373, 145-152.

Trognitz, B. R., Hermann, M. & Carrión, S. 1998 Germplasm conservation of oca (Oxalis tuberosa Mol.) through botanical seed. Seed formation under a system of polymorphic incompatibility. Euphytica 101, 133-141.

Trognitz, B. R., Carrión, S. & Hermann, M. 2000 Expression of stylar incompatibility in the Andean clonal tubar crop oca (Oxalis tuberosa Mol., Oxalidaceae). Sex. Plant Reprod. 13, 105-111.

Trognitz, B. R. & Hermann, M. 2001 Inheritance of tristyly in Oxalis tuberosa (Oxalidaceae). Heredity 86,564- 573.

Ueda, Y. & Akimoto, S. 2001 Cross- and self-compatibility in various species of the genus Rosa. J. Hort. Sci. Biotechnol. 76, 392-395.

Vaissière, B. E., Morison, N. & Subirana, M. 2004 Ineffectiveness of pollen dispensers to improve apricot pollination. Acta Hort. 701, 635-640.

Vaissière, B. E., Rodet, G., Cousin, M., Botella, L. & Grossa, J . P. T. 1996 Pollination effectiveness of honey bees (Hymenoptera: Apidae) in a kiwifruit orchard. J. Econ. Entomol. 89, 453- 461.

Valantin-Morison, M., Vaissière, B. E., Gary, C. & Robin, P. 2006 Source-sink balance affects reproductive development and fruit quality in cantaloupe melon (Cucumis melo L.) J. Hort. Sci. Biotechnol. 81, 105- 117.

Vaz, G. V., De Olivera, D. & Ohashi, O. S. 1998 Pollinator contribution to the production of cowpea on the Amazon. Hortscience 33, 1157-1159.

Vicens, N. & Bosch, J. 2000 Pollinating efficacy of Osmia cornuta and Apis mellifera (Hymenoptera: Megachilidae, Apidae) on 'red Delicious' apple. Environ. Entomol. 29, 235-240.

Vithanage, V. 1990 The role of the European honeybee (Apis mellifera L.) in avocado pollination, J. Hort. Sci. 65, 81-86.

Wallace, H. M. & Lee, L. S. 1999 Pollen source, fruit set and xenia in mandarins. J. Hortic. Sci. Biotech. 74, 82-86.

Wallace, H. M., Vithanage, V. & Exley, E. M. 1996 The effect of supplementary pollination on nut set of Macadamia (Proteaceae). Ann. Bot. 78, 765-773.

Watson, L. E., Bates, P. L., Evans, T. M., Unwin, M. M. & Estes, J. R. 2002 Molecular phylogeny of subtribe Artemisiinae (Asteraceae), including Artemisia and its allied and segregate genera. <http://www.biomedcentral.com/1471-2148/2/17>

Westerkamp, C. & Gottsberger, G. 2000 Diversity pays in crop pollination. Crop Sci. 40, 1209-1222.

Wei, S. G., Wang, R. Smirle, M. J. & Xu, H. L. 2002 Release of Osmia excavata and Osmia jacoti (Hymenoptera : Megachilidae) for apple pollination. Can. Entomol. 134, 369-380.

Weiss, J., Nerd, A., & Mizrahi, Y. 1993 Vegetative parthenocarpy in the cactus pear Opuntia ficus-indica (L.) Mill. Ann. Bot. 72, 521-526.

Westcott, L. & Nelson, D. 2001 Canola pollination: an update. Bee World 82,115-129.

Wieble, J., Chacko, E. K. & Downton, W. J. S. 1992 Mangosteen (Garcinia mangostana L.) A potential crop for tropical northern Australia. Acta Hort. 321,132-137.

Willmer, P. G., Bataw, A. A. M. & Hughes, J. P. 1994 The superiority of bumble bees to honeybees as pollinators – Insect visits to raspberry flowers. Ecol. Entomol. 19, 271-284.

Willson, M. F. & Schemske, D. W. 1980 Pollinator limitation, fruit production, and floral display in pawpaw (Asimina triloba). Bull. Torr. Bot. Club 107, 401-408.

Witter, S. & Blochtein, B. 2003 Effect of pollination by bees and other insects on the production of onion seeds. Presquisa Agropecuaria Brasileira 38, 1399-1407.

Wodehouse, R. P. 1931 Pollen grains in the identification and classification of plants. VI. Polygonaceae. Am. J. Bot. 18, 749-764.

Yaacob, O. & Subhadrabandhu, S. 1995 The production of economic fruits in South-east Asia. Oxford Univ. Press, Oxford, UK.

Yeboah Gyan, K. & Woodell, S. R. J. 1987 Analysis of insect pollen loads and pollination efficiency of some common insect visitors of four species of woody Rosaceae. Funct. Ecol. 1, 269-274.

Zebrowska, J. 1998 Influence of pollination modes on yield components on strawberry (Fragaria x ananassa Duch.). Plant Breed. 117, 255-260.

- 1. Noar Hill (red) surrounded by 3 concentric 300-m-wide areas (yellow lines) considered close, medium, and far from the reserve (yellow shading, rape fields; brown shading, field beans; triangles, sampling locations).


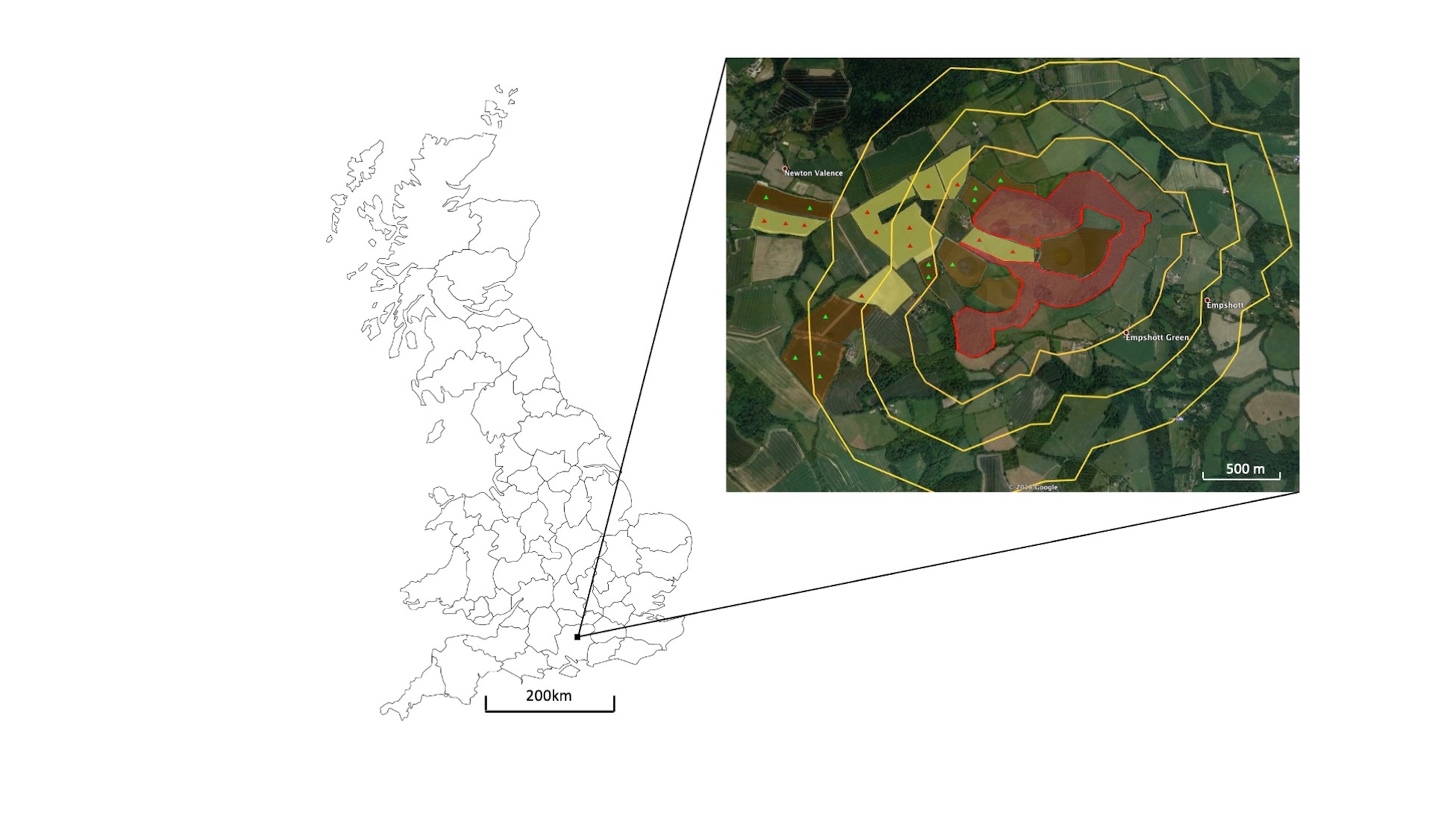


- 1. Questionnaire interviews template for local farmers at the Noar Hill reserve

| Farm Code | Crop 1 | Crop 2 | Crop 3 | Crop 4 | Crop 5 |
| --- | --- | --- | --- | --- | --- |
| What is the total size of the land you farm in the area (use local units of area if appropriate): |  |  |  |  |  |
| Which crops do you grow? |  |  |  |  |  |
| Unit of measurement for that crop |  |  |  |  |  |
| Maximum yield (in kg/ha or tonne/ha) achievable in the region for each of the crops you grow? |  |  |  |  |  |
| What are the production costs for each crop? |  |  |  |  |  |
| What is the total area (ha) of each crop, that you grow inside Noar Hill? |  |  |  |  |  |
| What is the total area (ha) of each crop that you grow in a 1km buffer from Noar Hill? |  |  |  |  |  |
| What is the farm gate price of the crops you grow in your farm |  |  |  |  |  |
